# Genetic diversity of variants involved in drug response among Tunisian and Italian populations toward personalized medicine

**DOI:** 10.1038/s41598-024-55239-7

**Published:** 2024-03-10

**Authors:** Haifa Jmel, Stefania Sarno, Cristina Giuliani, Wided Boukhalfa, Sonia Abdelhak, Donata Luiselli, Rym Kefi

**Affiliations:** 1 Laboratory of Biomedical Genomics and Oncogenetics, Institut Pasteur de Tunis, Tunis, Tunisia; 2 University of Tunis El Manar, Tunis, Tunisia; 3 Genetic Typing DNA Service Pasteur Institute, Institut Pasteur de Tunis, Tunis, Tunisia; 4 Laboratory of Molecular Anthropology & Centre for Genome Biology, Department of Biological, Geological and Environmental Sciences (BiGeA), University of Bologna, Bologna, Italy; 5 Laboratory of Ancient DNA (aDNALab), Department of Cultural Heritage (DBC), University of Bologna, Ravenna, Italy

**Keywords:** Computational biology and bioinformatics, Genetics, Biomarkers, Diseases, Endocrinology, Health care, Medical research, Molecular medicine, Risk factors

## Abstract

Adverse drug reactions (ADR) represent a significant contributor to morbidity and mortality, imposing a substantial financial burden. Genetic ancestry plays a crucial role in drug response. The aim of this study is to characterize the genetic variability of selected pharmacogenes involved with ADR in Tunisians and Italians, with a comparative analysis against global populations. A cohort of 135 healthy Tunisians and 737 Italians were genotyped using a SNP array. Variants located in 25 Very Important Pharmacogenes implicated in ADR were extracted from the genotyping data. Distribution analysis of common variants in Tunisian and Italian populations in comparison to 24 publicly available worldwide populations was performed using PLINK and R software. Results from Principle Component and ADMIXTURE analyses showed a high genetic similarity among Mediterranean populations, distinguishing them from Sub-Saharan African and Asian populations. The Fst comparative analysis identified 27 variants exhibiting significant differentiation between the studied populations. Among these variants, four SNPs rs622342, rs3846662, rs7294, rs5215 located in S*LC22A1*, *HMGCR, VKORC1 and KCNJ11 genes* respectively, are reported to be associated with ethnic variability in drug responses. In conclusion, correlating the frequencies of genotype risk variants with their associated ADRs would enhance drug outcomes and the implementation of personalized medicine in the studied populations.

## Introduction

The therapeutic failure and severe adverse drug reactions (ADR) remain one of the significant unmet medical challenges, representing major preventable causes of morbidity and mortality^1^. ADRs are the most frequent cause of all hospital admissions and rank as the sixth leading cause of death. Furthermore, ADRs increase healthcare costs due to extended hospitalization stay and supplementary clinical testing for severe cases^2,3^. ADR can be classified into two types:

*Type A reactions* represent dose-dependent and predictable reactions based on known pharmacological actions of the drug. Type A reactions are relatively common and include hypoglycemia induced by antidiabetic drugs and hemorrhagic reactions induced by anticoagulants, such as warfarin, acenocoumarol. These reactions represent 75% of the ADR, and are less likely to induce fatal consequences than type B. Despite their morbidity rate, ADR type A is not well recognized due to its progressive appearance^4^.

*Type B reactions* represent unpredictable pharmacological reactions and they are not necessarily dose-dependent. Type B skin reactions include skin disorders such as Stevens Johnson syndrome and toxic epidermal necrolysis. They represent serious reactions of skin hypersensitivity and liver damage^4,5^.

Growing evidence suggests the involvement of complex and independent factors in the onset of ADRs and drug effectiveness. The genetic background plays a crucial role in pharmacological reactions^6–11^. Over the last decades, pharmacogenetic studies identified several pharmacogenes and functional variants associated with drug response and helped in the identification of genes that can explain variation in drug response, known as pharmacogenes, as well as pharmacodynamics^10–15^. Variant–drug response associations have been established for many of these genes and this information can be translated into the clinical setting^16–18^. For example, approximately 18 percent of prescribed drugs carrying such pharmacogenomics labels in the USA^19^. Functional variants or risk alleles exhibit diverse distribution across ethnic groups, influencing the idiosyncratic response of drugs^2,20,21^. Around a fifth of newly approved drugs showed differences associated with interethnic variability^6^. In this specific instance, ibufenac employed in the treatment of rheumatoid arthritis, serves as an illustrative case. This drug was never approved in the U.S. and was withdrawn in the U.K. due to hepatotoxicity. However, ibufenac continues to be available in Japan, where hepatotoxicity is not considered a significant concern^22,23^. Pharmacogenetic stands out as an important determinant of inter-population variations in drug response^22^. This field focuses on identifying gene variants responsible for divergent drug responses, aiming to utilize them as predictive markers^24^. Inter-population frequencies variability in the frequencies of functional variants is common in the genes encoding for phase I drug metabolizing cytochrome P450s enzymes (*CYP2D6, CYP2C9, CYP2C19*), phase II enzymes catabolism metabolites (*UGT1A1, VKORC1*), drug transporters (*SLCO1B1, ABCG2, ABCB1*) and drug receptor (*P2RY12*)^25^.

Understanding ethnic contributions to drug efficacy and safety is crucial in the drug development process^6^. Establishing a registry containing pharmacogenetic data accessible to clinicians and the scientific community would be valuable for inferring population-level response rates and ADR risk for different medications. Such registers enable the identification of the populations requiring caution regarding the consumption of specific drugs^23,26^. A major challenge of performing this type of population level analysis is to remedy the paucity of data on ADR rates in different populations and determine the inherent mechanisms causing these significant proportions of ADR. These studies are of great interest in detecting the association between host genotype, ethnicity, and drug response. This could help formulate specific country-level guidelines on drug response, which are enormously useful and cost-effective in clinical practice^7,27^. While facing these challenges the past decade has witnessed a surge in pharmacogenetic studies due to the rapid increase in human genome data and the development of high-throughput genotyping technologies. Consequently, ADR occurrences have significantly decreased^18,28^.

However, few previous studies have focused on exploring pharmacogenomic diversity of Mediterranean populations.

In this context, our aim is (1) to explore the diversity of variants located in pharmacogenes involved in drug biotransformation and ADR among individuals from the Tunisian and Italian populations and (2) to compare our findings with the variability observed among worldwide populations. We choose the Tunisian and Italian population because the Tunisian population is characterized by high genetic heterogeneity^29^. This could be due to several historical events such as migration and invasion^30,31^. Previous studies have revealed the genetic mosaicism of the Tunisian population, encompassing different ethnic groups, especially of European ancestry^29,30,32^. Given the geographical proximity of Tunisia to Italy and the historical events between these populations, we suggest the presence of a common genetic background between them. This could contribute to a better understanding of the pharmacogenetic response to some drugs and thus to the development of tailored therapeutic strategies for these populations.

## Results

### Population structure at pharmacogenomics loci

Based on an extensive data mining search and PharmGKB interrogation, we selected 25 pharmacogenes implicated in drug response modulation and ADR (Table 1). A total of 148 variants located on the 25 pharmacogenes have been identified in the Tunisian population and kept after quality control steps for subsequent analysis. Only 138 variants have been identified as shared among Tunisian and Italian populations. After applying the threshold criteria of quality control, we have kept out 116 shared variants. To study the genetic diversity of these variants, we compared the genotypic data of the shared variants to those of all 1000 genomes project populations.

**Table 1:** Basic Information about the selected pharmacogenes.

Drug class	Drug name	VIP	Chr	VIP variant	Toxicity level	Phenotype	Population	Refs.
Anticoagulant	Clopidogrel (Palvix)	*ABCB1*	7	rs1054564 rs2032582	2A	Cardiovascular complications Coronary artery disease	North African European	^34,35^
					3			
		*CYP2C19*	10	rs1057910 rs4244285	1A 1A	Acute coronary syndrome coronary artery disease/Myocardial infarction	Mixed population	^32,36^
		*P2RY12*	3	rs6785930	4	Neurological events	European	^37,38^
	Warfarin Acenocoumarol	*CYP2C9*	10	rs1057910	1A	Hemorrhagic events	Asian	^39^
		*VKORC1*	16	rs9923231 rs7294	1B 1A	Hemorrhagic Venous thrombosis	African American _Asian	^39,40^
Antidiabetic	Biguanide/Metformin	*SLC22A1*	6	rs628031	3	Gastrointestinal toxicity	European	^41^
	Biguanide/Metformin	SLC22A2	6	rs12943590	3	Gastrointestinal toxicity and Hypoglycemia		^38^
						Nephrotoxicity		
	Biguanide/Metformin	*SLC47A1*	17	rs2289669	3	Efficacy*	Chinese	^42^
	Biguanide/Metformin	*SLC47A2*	*17*	rs12943590	3	Efficacy**	Chinese	
								^43^
	Biguanide/Metformin	*AT M*	11	rs11212617	2B	Efficacy: Great reduction of Fasting Plasma Glucose	Caucasian	^44^
							Chinese	^43^
	TZD/Pioglitazone	*PPARG*	3	rs1801282	3	Oedema	European	^45^
	TZD/Rosiglitazone	*PGC-1alpha*	5	rs1801282	3	Efficacy	Mixed population	^46^
	TZD/Troglitazone	*RETN*	19	rs1862513		lower fasting blood glucose	Caucasian and Asian	^47^
		*Leptin LEPR (G)*	1	rs1137101	3	Hyperglycemia and T2D susceptibility	Chinese	^48^
	Sulfonylurea	*TNFalpha*	2	rs1800629	3	Hyperglycemia	Egyptian	^49^
		*KCNJ11*	11	rs5219	2A	Severe hypoglycemia		^50,51^
						Microvascular Neuropathic compilation		
		*ABCC8*	11	rs757110	3	Anaphylactic shock Serum sickness-like syndrome	Espagnol	^51–53^
						Systemic allergic vasculitis	German	
							Chinese	
		*KCNQ1*	11	rs2237892, rs2237895	3	Efficacy in term of postprandial plasma glucose level	Chinese population	^54^
		*TCF7L2*	10	rs12255372	3	Efficacy	European	^55^
		*CYP2C9*	10	rs1057910	1A	Hypoglycemic attacks	American	^56^
				rs1799853				
Lipid-lowering	*Fluvastatin*	*SLCO1B1*	12	rs4149056	1	Myopathy	European	^57^
	Lovastatin	*ABCB1*	7	rs1128503	3	Hypercholesterolemia	European	^6,58^
	Atorvastatin	*ABCB1*	7	rs2032582	2A	Hypercholesterolemia	European	^58^
	Pravastatin	*ABCA1*	9	rs12003906		Myocardial infarction	European	^60^
		*CYP2C9*	10	rs1057910	1A	Epilepsy	Asian and European	^61,62^
		*PPARA*	22	rs4253728	3	Cardiovascular complication	American	^63^
		*SLCO1B1*	12	rs4149056	1A	Myopathy	European	^64^
		*HMGCR*	5	rs17238540	4	Cardiovascular complication	African and European	^38^
		*CYP3A5*	7	rs776746	3	Hypercholesterolemia and Myalgia	European	^65^

The table describes the selected pharmacogenes and variants implicated in the ADR of several anticoagulants, antidiabetics, lipid lowering drugs, FINS: fasting insulin, glycated hemoglobin A1 (HbA1C), HOMA-IS: homeostasis model assessment-insulin resistance.

*Higher reduction in Hba1c reduction, FINS and HOMA-IR.

**Higher Hba1c reduction by elevating the circulating concentration of metformin. According to clinical guidelines, toxicity level of evidence is classified as follows: 1A, 1B: high toxicity, 2A, 2B: moderate toxicity, 3: low toxicity, 4: lack of research supporting the association of the variation with toxicity.

In order to assess the degree of similarity in genetic structure between the different populations, we calculated pairwise Fst values to evaluate the magnitude of differentiation among them. Pairwise Fst values between the Tunisian and the Italian populations ranged from 0.0014 to 0.004 (Supplementary Table 2). Comparing Tunisian and Italian to other populations, the lowest level of differentiation was observed between the Tunisian and European populations, whereas the highest divergence was observed with Sub Saharan African populations.

The MAF of highly selected actionable pharmacogene variants among TUN and ITA populations (rs622342, rs7294, rs5215 and rs3846662) were compared with EUR and AFR populations (Table 2). Our results revealed that rs622342 located in *SLC22A1* showed no significant differences between the TUN population and the EUR (N_ITA, C_ITA, S_ITA, TSI, and IBS) and AFR (YRI, LWK, ESN, GWD, MSL) populations (p > 0.05/4 × 14). No significant differences were found between TUN and central, southern Italian, and AFR (YRI, ESN, and MSL) populations regarding the rs5215 variant located in *KCNJ11*. However, the MAF of rs5215 showed significant differences between the TUN population and northern Italian and SARD populations. In addition, The MAF of the rs7294 variant located in the *VKORC1* gene was significantly different between the TUN, EUR, and AFR subgroups (p < 0.05/4 × 14). Regarding the rs3846662 variant located in the *HMGCR* gene, we found significant differences between TUN and AFR (YRI, LWK, ESN, GWD, MSL) populations.

**Table 2:** Frequency comparison of actionable pharmacogene variants shared between Tunisian and Italian populations.

Gene	PGx-Variant	PGx Variants	SARD	N_ITA	C_ITA	S_ITA	TSI	IBS	CEU	GBR	FIN	YRI	LWK	ESN	GWD	MSL	Relevant Drug
*SLC22A1*	rs622342	C	0.031	0.116	0.116	0.116	0.116	0.002	0.031	0.062	0.031	0.376	0.731	0.284	0.731	0.284	Metformin
*VKORC1*	rs7294	C	**1.42E-08**	**4.59E-10**	**4.59E-10**	**4.59E-10**	**4.59E-10**	**1.30E-06**	**1.42E-08**	**2.67E-09**	**1.42E-08**	**2.20E-16**	**2.20E-16**	**2.20E-16**	**2.20E-16**	**2.20E-16**	Warfarin, Acenocoumarol
*KCNJ11*	rs5215	C	**0.0002**	**0.0007**	0.0016	0.0015	**0.0027**	**1.26E-05**	**0.0001**	0.001	**4.31E-05**	1	**5.79E-01**	1	**5.79E-01**	1	Biguanide , Metformin
*HMGCR*	rs3846662	A	0.043	0.67	0.887	1	0.57	0.393	0.47	0.777	0.776	**3.80E-15**	**4.02E-16**	**4.02E-16**	**2.92E-14**	**3.80E-15**	Paravastatin

The table shows the MAF comparison of highly selected actionable pharmacogene variants (rs622342, rs5215rs7294, rs622342 and rs3846662) linked to ADR among TUN and ITA populations, obtained after Bonferroni correction. Variant with significant differences (p-values < 0.05/4 × 14) are shown in boldface.

**Figure 1:**
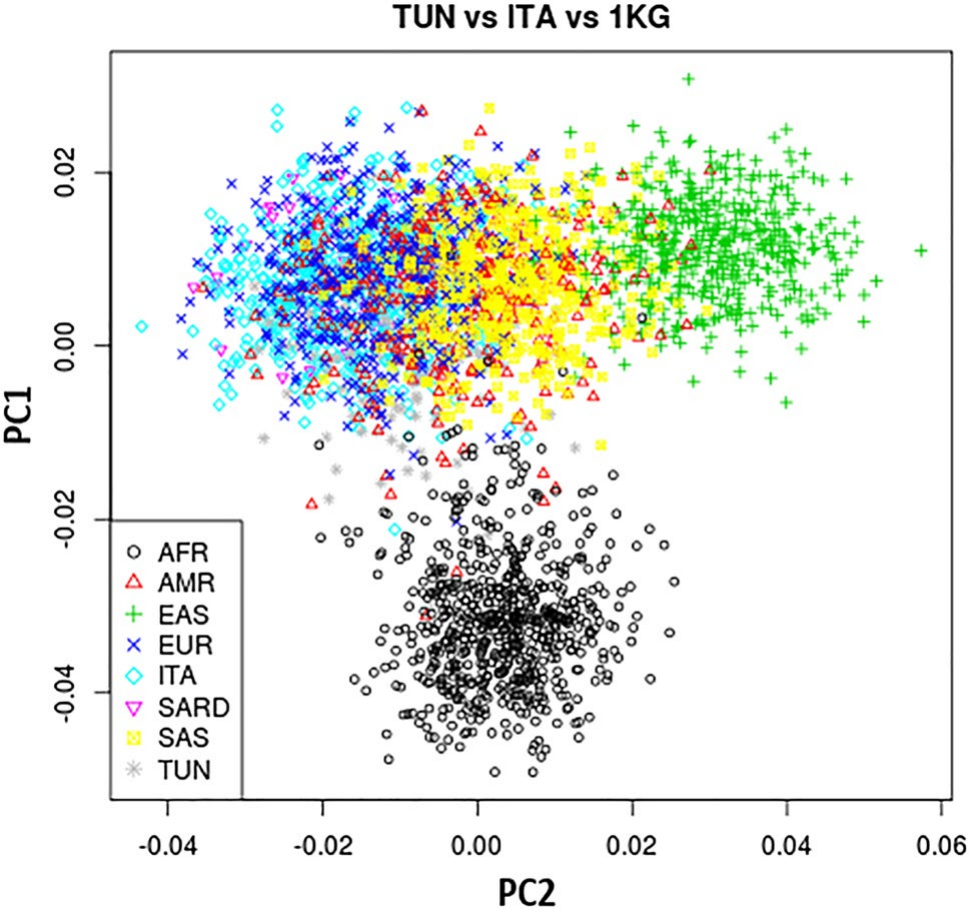
Principal Component Analysis Plot of the Tunisian “TUN” subpopulation, Italian (ITA) population and the 1000 genome project populations originating from Sub-Saharan Africa (AFR), America (AMR), East Asian (EAS), EUR (Europe), Sardinia (SARD), South Asian (SAS).

**Figure 2:**
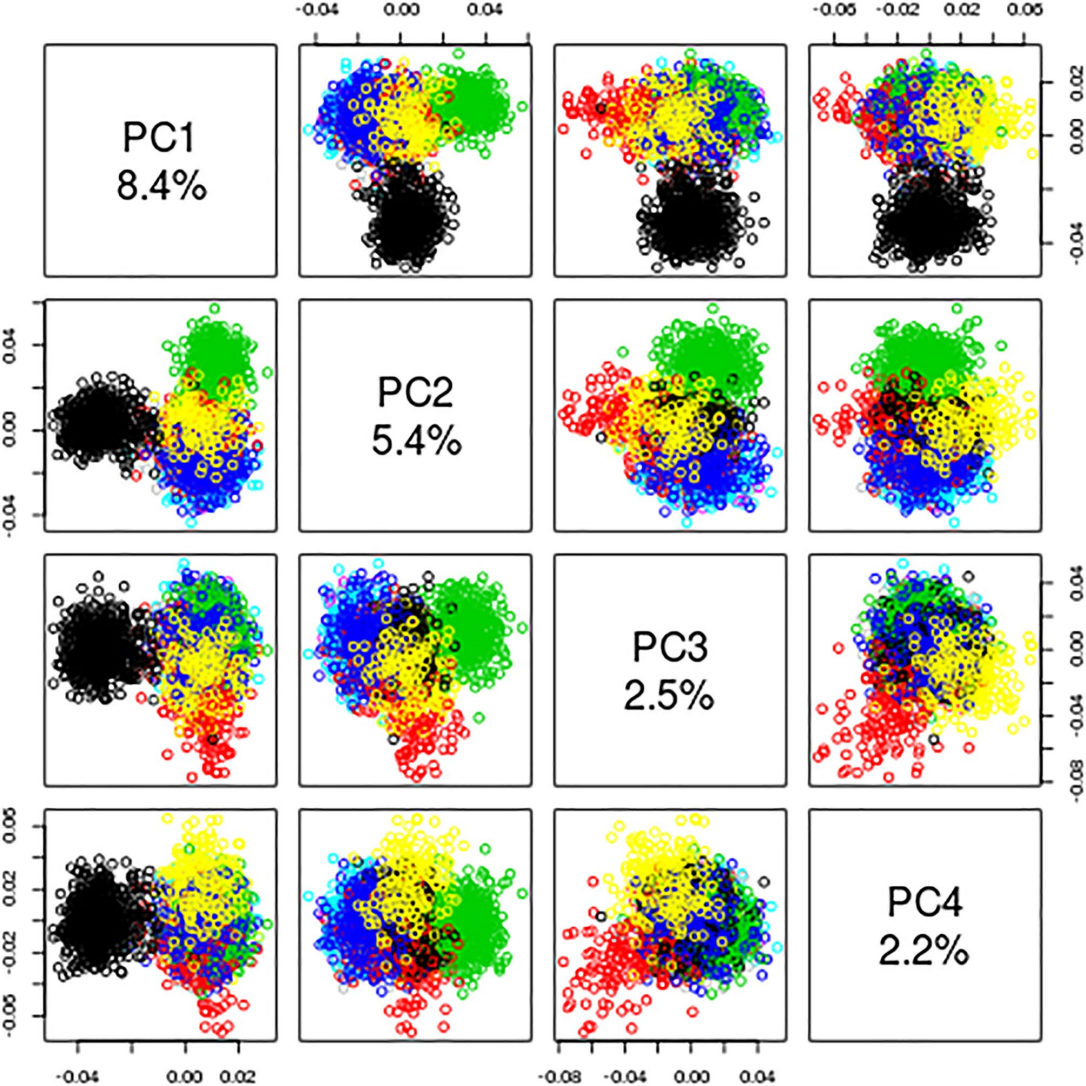
*Scatter Plot* analysis of the PCA representative of the Tunisian and Italian populations compared to the populations of the 1000 Genomes project. The result of the *Scatter Plot* shows that PCA (Fig. 1) has the best estimated orientation of 8.4%. This is the highest percentage between the four prisms showing all possibilities of the PCA orientations.

PCA revealed that the Tunisian population is clustered with the Italian population (Central, North and the South), European (EUR) and American (AMR) populations. A greatest divergence was observed with the East Asian (EAS) and the African (AFR) populations (Fig. 1). The scatter plot shows that the PCA has the best percent of explained variance for PC1, estimated at 8.4%. This is the highest percentage among the four prisms displaying all the orientation possibilities of the PCA (Fig. 2).

**Figure 3:**
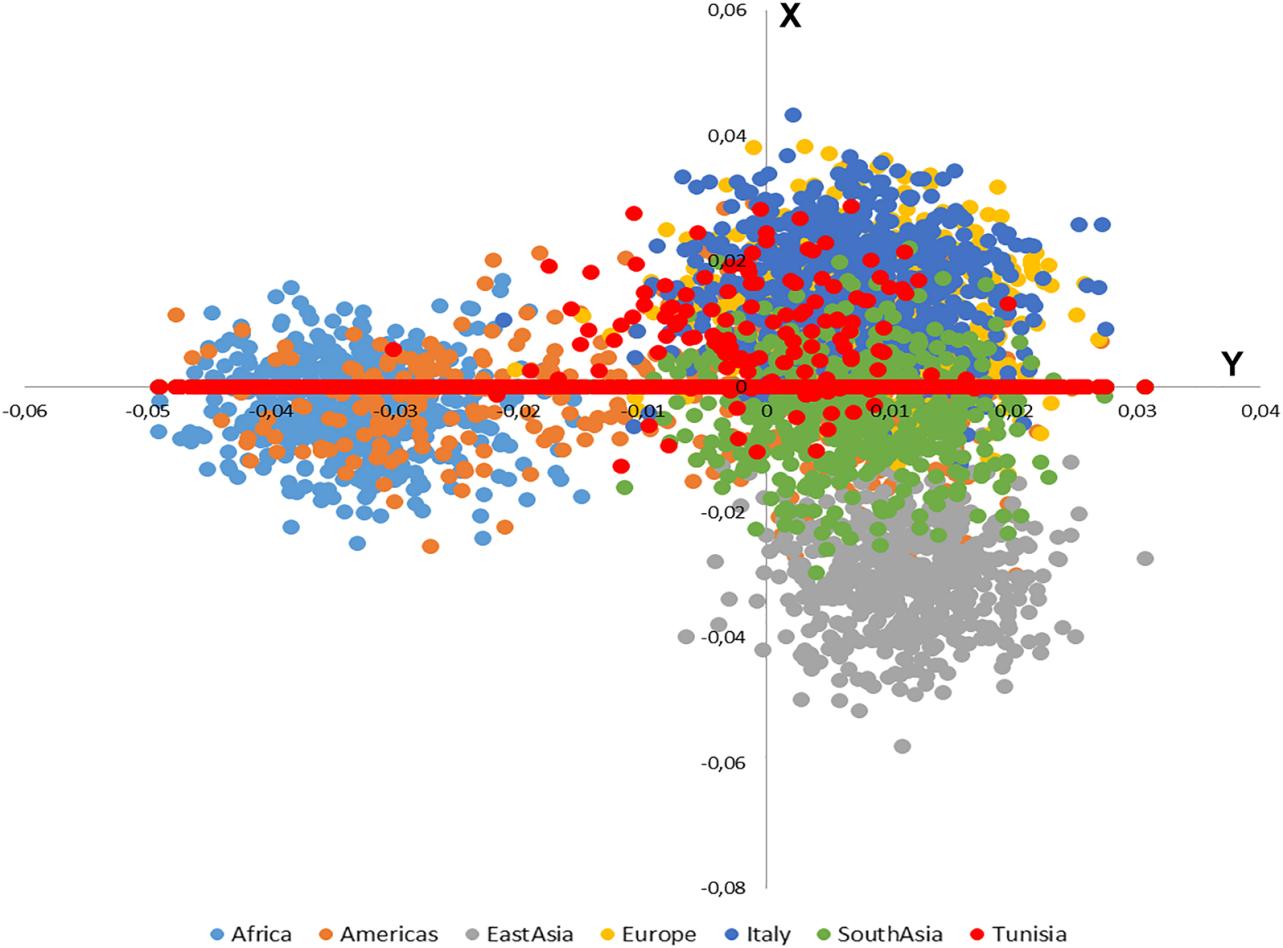
Comparison between the Tunisian and Italian populations with all the other European populations (combined in a single entity) and Sub-Saharan Africa populations of the 1000 Genomes project.

**Figure 4:**
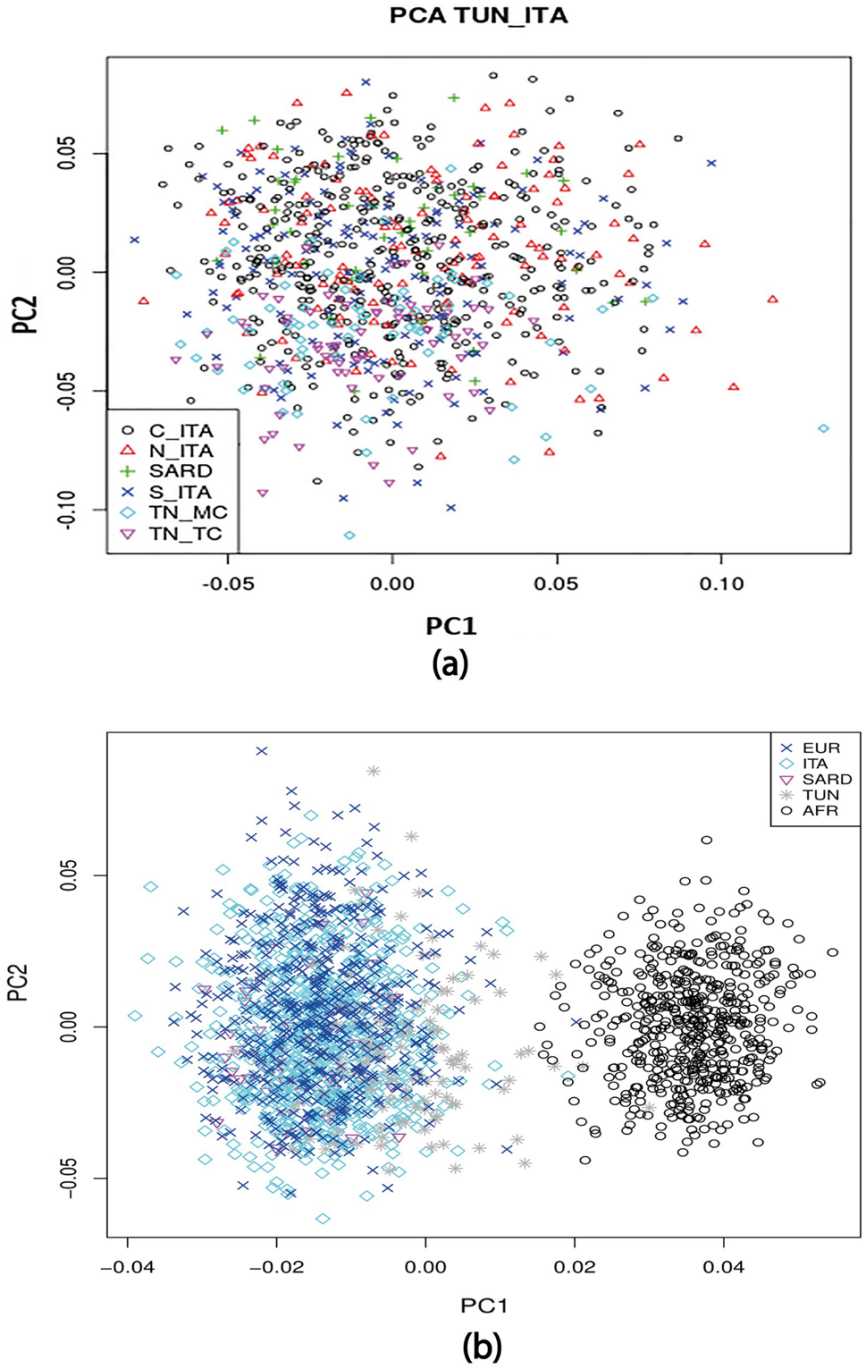
Principal Component Analysis plots of different population combinations. C_ITA: Italians from the Center, N_ITA: Italians from the North, Italian from Sardinia, S_ITA: Italians from the South, TN_MC: Tunisians originated from the coastal city of Monastir, TN_TC: Tunisians originated from the Capital Tunis, AFR: Sub-Saharan African populations.

The comparison of the different studied populations clustered in four continents (African, European, American and Asian) shows a high level of similarity between Tunisian population and the Italian population. On the other hand, the African was distinguished from the European and the North American continents, projected on the opposed factorial axis (Fig. 3). The PCA regrouping Tunisian and Italian population sub-groups reveal the genetic similarity between these two populations (Fig. 4).

**Figure 5:**
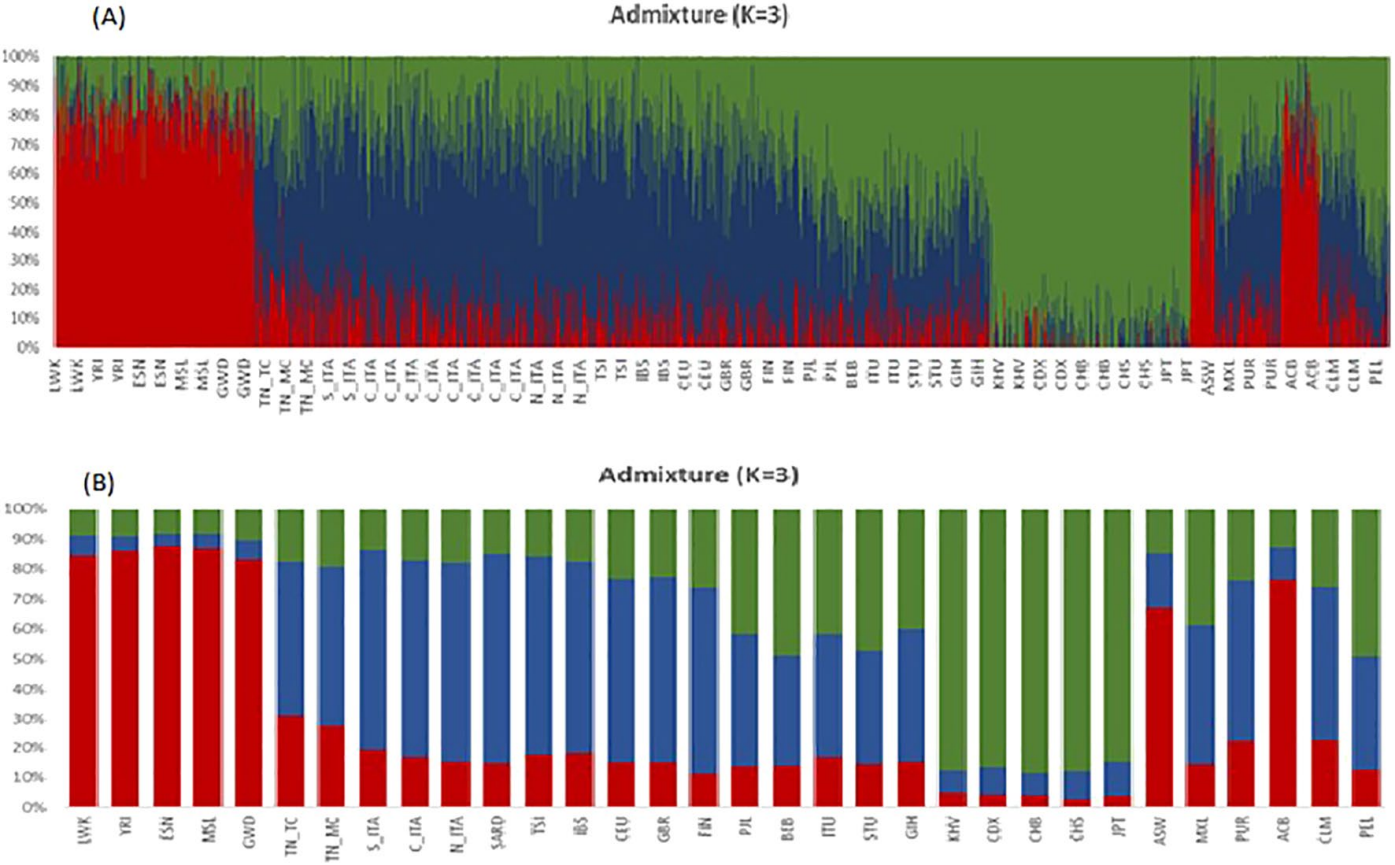
ADMIXTURE individual bar plots (K = 3). (A) Admixture plot_ 27 populations, 116 variants studied—displaying results for runs with highest likelihood out of 27 runs in each cluster K3 to 10. Black vertical lines identify the population boundaries. The height extent of each color within an individual’s color bar corresponds to the estimated membership of the individual in one of the clusters; each cluster is assigned a separate color. The bars with multiple colors can be interpreted as genetic admixture or as relative probabilities of belonging to the different clusters. The graphic of ADMIXTURE analysis demonstrates three components with the predominance of African, European components at the number of ancestor K = 3. (B) Individual Admixture Proportions: individual are represented by a single vertical line broken into three colored segments, with lengths proportional to each three inferred clusters; red indicates African ancestry proportion, blue indicates European ancestry proportion and green indicates East Asian proportion. The coordinate indicates the proportion unit.

**Figure 6:**
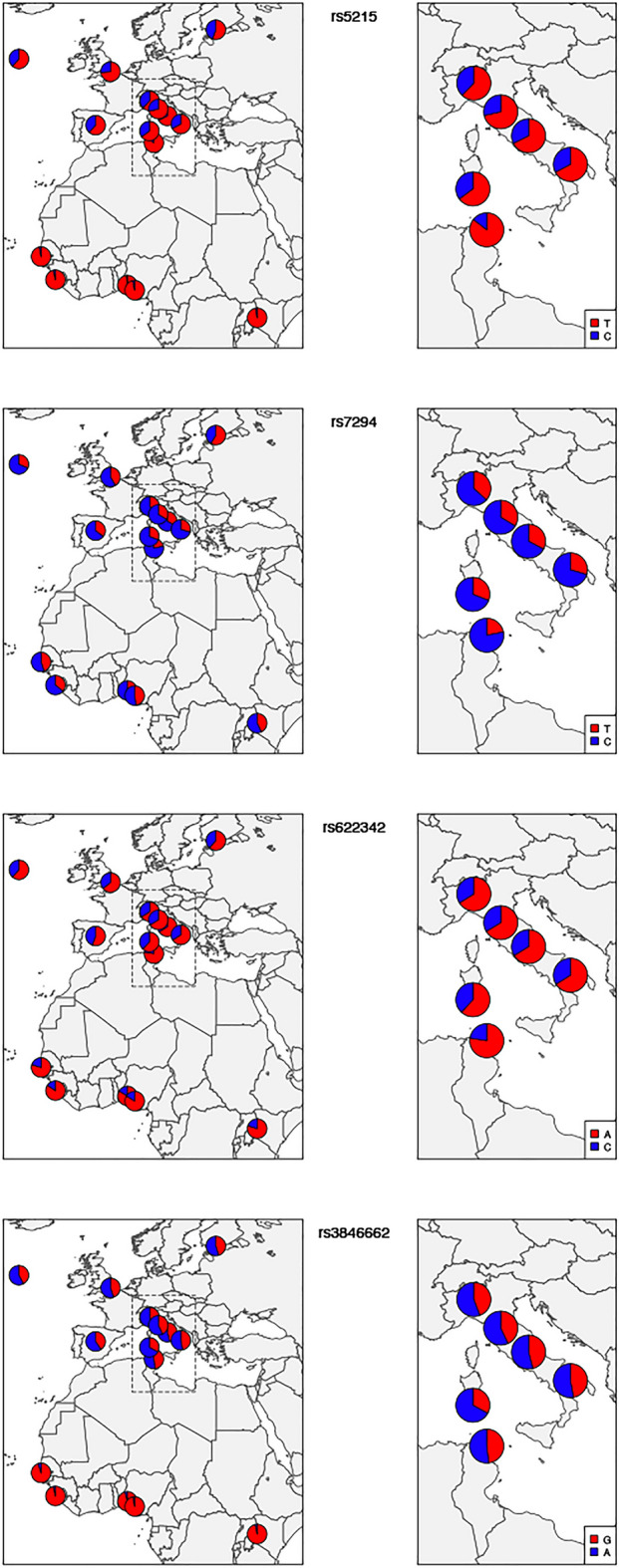
Distribution of allele frequencies of clinically relevant variants in Tunisian, Italian, European, African and South Asian populations. The graph shows an inter-population diversity of the frequency distribution of the dominant and recessive alleles of the variants rs622342, rs3846662, rs7294, rs5215 located respectively in the “pharmacogenes” *SLC22A1*, *HMGCR*, *VKORC1*, *KCNJ11*. Red and blue colors indicate allele frequencies. We generated geographical map using R software [v.3.2.4] (https://www.rproject.org/), by using the packages rworldmap (URL: https://cran.r-project.org/web/packages/rworldmap/) and mapplots (URL: https://cran.r-project.org/web/packages/mapplots/index.html).

**Figure 7:**
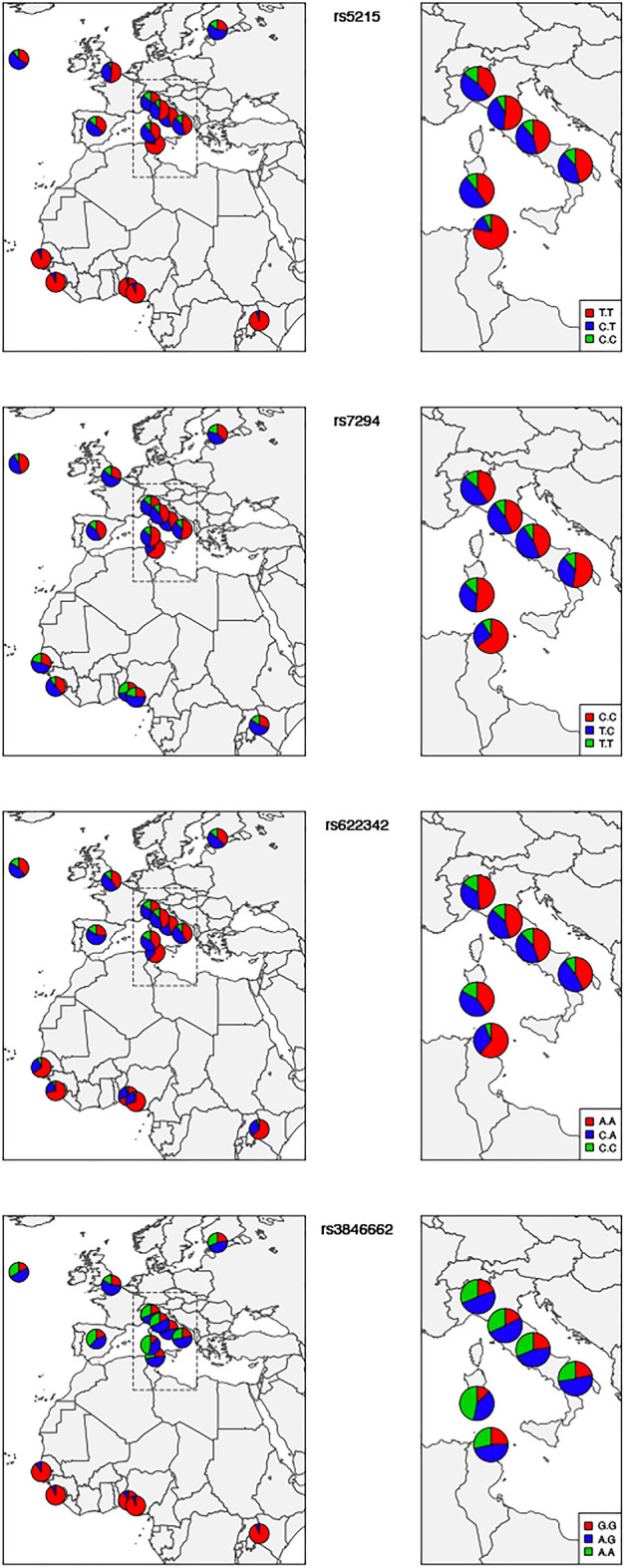
Distribution of genotypic frequencies of clinically relevant variants in Tunisian, Italian, European, African and South Asian populations. The graph shows an inter-population diversity of the genotype frequency distribution of the variants rs622342, rs3846662, rs7294, rs5215 located respectively in the “pharmacogenes” *SLC22A1, HMGCR*, *VKORC1*, *KCNJ11*. The red color indicates the frequency of wild homozygous, the blue color; the frequency of heterozygous and the color green; the frequency of recessive homozygous. We generated geographical map using R software [v.3.2.4] (https://www.rproject.org/), by using the packages rworldmap (URL: https://cran.r-project.org/web/packages/rworldmap/) and mapplots (URL: https://cran.r-project.org/web/packages/mapplots/index.html).

Next, ADMIXTURE analysis was employed to infer ancestral population groups. The K = 3 was identified as the most likely number of genetic clusters and corresponding to the three ancestral continental population groups; European (S_ITA, C_ITA, SARD, TSI, IBS, CEU, GBR, FIN), Asian (CHB, CHS, CDX) and Sub-Saharan African (LWK, YRI, MSL, GWD, TN_TC, TN_MC). The ADMIXTURE analysis graphic reveals the predominance of African and European components to this number of ancestor K ancestries (Fig. 5). The findings indicate that the Tunisian population, in terms of the studied pharmacogenes, were more similar to the European and particularly Mediterranean populations (average frequency of 45%). This result corroborated the patterns seen on PCA (Fig. 1). The evidence of Fst, PCA, and ADMIXTURE analyses shows that there is a high similarity among Mediterranean populations which are genetically divergent from the Sub-Saharan Africa populations. Then after the description of the general pattern of variation including all the SNPs we performed a Fst comparative analyses to identify the most divergent SNPs. We identified 26 variants with high levels of differentiation between Tunisian, Italian and other studied populations (Table 3). Among these variants, there are four SNPs rs622342, rs3846662, rs7294, rs5215 located respectively in *SLC22A1*, *HMGCR*, *VKORC1*, *KCNJ11*, and reported as associated with ethnic variability in drug response. The other 22 variants are not yet described to be associated with any pharmacogenetic variability (Table3). Moreover, the Fst comparison analyses show that the variant rs5215 located on *KCNJ11* is the most differentiated variant between the Tunisian and Italian populations. This variant is associated with the resistance to oral antidiabetics (Metformin, Glucamide, Sulfamides.) (Supplementary Fig. 1). The variant rs7294 located on *VKORC1* associated with warfarin dose requirement, shows a large differentiation between Tunisian and British populations from England and Scotland (GBR). However, this variant reveals a similarity among Tunisian, Italian and all the other studied populations (Table 3) (Supplementary Fig. 1). The variant rs622342 located on *SLC22A1* associated with the efficacy of metformin shows a significant differentiation between Tunisian and the Iberian (IBS) populations and notable similarity with Italian population (Table 3) (Supplementary Fig. 1). The rs3846662 variant located on *HMGCR,* associated with the reduced Pravastatin efficacy and smaller LDL cholesterol reduction, displays substantial genetic differences between the studied populations. Furthermore, these results were confirmed by genotype and allelic distributions of the four high selected VIP variants (p < 0.05), using the worldwide representative map (Figs. 6, 7). The genotype frequencies comparison shows that GG and AG genotypes of the variant rs3846662 located in *HMGCR* gene, associated with the reduced effect of pravastatin, represent high frequencies in the Sub-Saharan Africa population compared to other studied populations (Fig. 7). The frequency of GG, AG genotypes of rs3846662 variant in the Tunisian population are 24.81% vs 46.62%., these frequencies are very similar to those observed in the Italian populations which are 23.08% vs 45.66 in the Center, 19.85% vs 48.85% in the North, 21.79% vs 50.64% in the South and 12.77 vs 40.43 in Sardinia (Supplementary Table 4). These frequencies are moderately low compared to those of Sub-Saharan Africa which has a GG frequency equal to 95% (Supplementary Table 4) (Fig. 7).

**Table 3:** Description of VIP variants through the comparison of their Fst values.

	CEU	FIN	GBR	IBS	TSI	N_ITA	C_ITA	S_ITA	SARD	GWD	MSL	LWK	ESN	YRI
rs11023996		*												
rs11196212			*											
rs7294			*											
rs11196224			**											
rs622342				*										
rs10832785				*										
rs11603988				*										
rs10889503					**	*				**	**	**	*	*
rs887241						*								
rs316013								*						
rs3777406									*					
rs7931930									*					
rs234852								*	*					
rs12654264						**	**	**	**					
rs2228100						**	**	**	**					
rs1116714	*	*	*											
rs10064799	*	*			*		*							
rs7757997	*				*		*	*						
rs5215	**	**		**	*	*	*	*						
rs11656096	**	**	**	**	**	*	*		*					
rs1555485	*	*	*	*	*									
rs4916014										**	**	*	*	**
rs11208591										*	*	**	**	*
rs6007919										*	*	*	**	*
rs4915675										*	*	*	*	**
rs3846662										*	*	*	*	*

The statistical test of Chi2 was used to compare the Fst values of the variant: Highly selected variants with statistical significance differentiation (p < 0.05) are with *. Variants with high statistical significance differentiation (p < 0.01) are in **. * for p < 0.05 and ** for p < 0.01.

The distribution of the genotypes for the variant rs7294 (C > T) located in *VKORC1*gene is as follows. The genotypes TT and CT associated with a high dose of warfarin are for the Tunisian population of 64.84% and 26.56%. These frequencies align closely with those observed in the Italian population with values of 44.03% vs 46.52% in the Center, 40.46% vs 45.8% in the North and 52.56% vs 35.90% in the South (Supplementary Table 4). The frequency of the recessive homozygotes TT is 8% in the Tunisian studied population, resembling that in the Italian population (Center 9.45%, North 13.7%, South 11.5%) (Supplementary Table 4). In the Tunisian population, the frequency of the rs7294-T allele is 21.88%. Similarly, in the Italian population, the frequency varies across regions, with values of 36.64% in the North, 32.71% in the Center, and 49% in the South (see Supplementary Table 3) (see Fig. 6). Comparatively, these frequencies are notably lower in both the Tunisian and Italian populations when contrasted with Sub-Saharan African populations, where the rs7294-T allele frequencies are 51.39% in YRI, 42.93% in LWK, and 45.13% in GWD (see Supplementary Table 3) (see Fig. 6).

## Discussion

The distribution of genetic polymorphisms located in pharmacogenes involved in drug metabolism and transport are major determinants of treatment efficacy and adverse reactions. Biogeographical ancestry is an important factor leading to large diversity in drug effectiveness or adverse reactions. In the present study, we showed that some Very Important Pharmacogenes ‘VIP’ variants which involved in weight loss drugs, lipid-lowering, antihypertensive and oral antidiabetics agents, exhibited a great genetic variation among the studied populations. This variability impacts directly on the delivery of individualized medicine.

### Genetic landscape of the selected pharmacogenes

The PCA, pairwise Fst values and ADMIXTURE analyses reveal a high similarity among Mediterranean populations concerning the studied pharmacogenes. In addition, these analyses exhibit a great genetic divergence between Mediterranean populations and Sub-Saharan Africa and Asian populations.

The different combinations of PCA show that Tunisian population is genetically related to the European and especially to the Italian population. Furthermore, the shift suggests slight genetic differences but the lack of distinct clusters suggests common ancestral links (Fig. 4). These observations were also reported for mitochondrial DNA, Y chromosomal and autosomal markers and interpreted as influences from different migration events^30,31,33–36^. Obviously, differences in admixture history exert an important impact in the allelic and genotypic distribution of variants at the population level^36^. In the present study, four polymorphisms characterized as clinically relevant VIP variants were selected, based on previous pharmacogenomics research. These four polymorphisms (rs622342, rs3846662, rs7294 and rs5215) have been studied with a low level of clinical evidence in PharmGKB. As explained in this database, the low score may be based on a single study annotated in PharmGKB, or there may be several studies that failed to replicate the association. Therefore, further association studies should be performed to investigate these variants and to determine the population specificity in order to personalize treatments. Investigating the prevalence of pharmacogene risk variants in target groups, together with actual drug consumption data, will allow better therapeutic decisions^37^.

### Genetic variability of the selected pharmacogenes

Our genetic distribution study showed that TT and CT genotypes of rs7294 (C > T) located in the *VKORC1* gene, associated with a high dose of warfarin requirement, were more common in the GBR population than in other populations. This finding implies that the British population has poor warfarin metabolism, which raises the risk of a bleeding event. Our result was consistent with the study of Jones et al., 2005, who discovered that poor clinical outcomes were associated with suboptimal anticoagulation in British patients. New measures must be implemented to improve maintenance anticoagulation in British patients with nonvalvular atrial fibrillation^38^.

Our result showed that the genotype frequency of GG and GA of the rs3846662 (A > G) variant located on *HMGCR* associated with the reduced effect of pravastatin was higher in Sub-Saharan Africa populations than in other studied populations. This data suggest that Sub-Saharan Africans were resistant to this treatment. This outcome was in agreement with those of Medina et al. 2008 which highlighted that GG and GA of the variant rs3846662 were associated with decreased induction of full-length transcripts of HMGCR and increased expression of the spliced HMGCRv_1 transcript as compared with AA genotype. The author of this study explained that the variant rs3846662 genotype by itself was not significantly associated with statin response^39^. The HMGCRv_1 was suggested as a marker for statin therapy efficacity^40^. However, it is important to note that rs3846662 is not the only determined of statin response^41^.

The comparison of the genotype and minor allelic distribution revealed that the VIP variants in Tunisian population were genetically similar to Italian and other European populations and divergent from Sub-Saharan Africa and Asian populations. Nonetheless, our study has shown some exceptions. The rs5215 (C > T) variant located in *KCJN11* gene showed the highest significant differentiation between Tunisian and Italian populations (Table 3). This variant was associated with the resistance to oral antidiabetic drugs such as (glimepiride, gliclazide, gliquidone, glipizide, glibenclamide). The reported variant was in perfect high LD with the variants rs5219 in both Tunisian and Italian populations. Multiple studies have revealed the association of rs5219 to the resistance of sulfamide-inducing hypoglycemia, hypoglycemic coma, hypersensitivity, hepatotoxicity, drug-induced erythema multiforme and photodermatitis^42^. Type 2 diabetes (T2D) patients with the CC genotype of rs5219 treated with metformin and sulfonamides showed a decreased likelihood of treatment failure and a higher HbA1c concentration than patients with TT genotype. We can speculate that the C allele of rs5215 is more represented in the Italian population and is also associated with the resistance to the two main widely administered oral antidiabetics, metformin and sulfamide^43^. This result is in agreement with the study of Sesti et al. which reported the failure of the combination of sulfonylurea with metformin, rather than the failure of sulfonylurea treatment itself. The study included 525 Italian patients and showed that carriers of the C allele of rs5219 had a significantly higher probability of secondary sulfonylurea failure. The patients were treated with a combination of sulfonylurea as the first-choice drug and metformin as an add-on drug^44^. The defective C allele is more prevalent in Italian and European populations than in the Tunisian and Sub-Saharan Africa populations. We hypothesize that Europeans have a higher risk of treatment failure when combining sulfonylurea and metformin than African individuals.

Furthermore, rs622342 (A > C) located in *SLC22A1* gene is associated with inter-individual metformin response variability. Our results showed that the risk allele “C” of rs622342 (SLC22A1) associated with Metformine intolerance showed significant differences between Tunsian, European and African populations. This variant has been reported by previous study as potentially associated with glycemic response to metformin in European and African populations. However, no metformin intolerance has been shown. This evidence may provide novel insight into gene-oriented personalized medicine for diabetes^45^.

The carriers of CC and the CA genotype of rs622342 were associated with poorer response to metformin, as measured by a smaller reduction in HbA1c levels, compared with AA patients^46,47^. Our finding revealed that carriers of the CC and CA genotypes were more frequent in Italian and European populations than in Tunisian populations. The Tunisian population appeared to be a better responder to oral antidiabetics. This information could be clinically relevant predicting the glucose-lowering effects of metformin in different ethnicities before the start of therapy.

The rs3846662 variant was associated with the variation in the production of the HMGCR isoform, correlated with reduced sensitivity to statins. As a result, it may play a significant role in the interindividual variations in LDL, apolipoprotein B, and triglyceride concentrations during statin therapy. More precisely, the GG and AG rs3846662 variant decreased induction of full-length transcripts of HMGCR and increased expression of the spliced HMGCRv_1 transcript as compared with AA genotype^39^. Our analysis revealed a high frequency of the GG and AG genotypes of rs3846662 in Sub-Saharan African populations compared with the other studied populations. These differences may affect the effectiveness of simvastatin and decrease the risk of myopathy^48^. Taking these pieces of evidence into consideration, we hypothesize that Mediterranean and European populations are good responders to simvastatin as the treatment of choice for LDL decreases. In contrast, Sub-Saharan Africans are simvastatin-resistant, which explains the increased rate of cardiovascular disease in this group. This finding is consistent with the CAP study in which African-Americans had a weaker statin LDL-C response than Caucasians. However, our results contradict the finding of Akinyemi Oni-Orisan et al. who found that participants in East Asian, Hispanic, and African populations responded to statin medication with larger percentage reductions in LDL-C than participants in European populations^50^. This outcome might be the result of the synergic effect of variants linked to the statin response variability.

The fourth SNP is rs7294, located on the *VKORC1* gene and is an essential anticoagulant cofactor in vitamin K metabolism. The rs7294 TT and CT carriers required a higher warfarin dose. Our analysis showed that Tunisian, Italian, and other European (except Spanish) populations presented similar frequencies of these genotypes. However, Sub-Saharan Africans have a lower frequency of these genotypes. These could explain the differences in warfarin dose administration among populations. This result is well illustrated in (Fig. 7). Our results are consistent with the study by Yang et al., who showed that Sub-Saharan African populations require a low dose of warfarin to achieve the therapeutic effect compared with Caucasian and Asian populations^51^. We mention that in Tunisia, since 2016, warfarin is no longer adopted in the therapy of thromboembolic complications. The anticoagulant acenocoumarol has become a clinician’s therapeutic choice for the treatment of hypertension complications. Indeed, patient carrying TT and CT for the variant rs7294 may require higher doses of acenocoumarol than the CC wild-type genotype^52^. Previous studies have shown that the “T'' recessive allele is the defective allele, associated with resistance to acenocoumarol. On the other hand, the “C” allele is associated with the efficacy of acenocoumarol. Our results showed a low prevalence of the risk “T” allele of the variant rs7294 in the Tunisian and Italian populations compared to Sub-Saharan Africa populations. Consequently, Tunisian patients carrying the ancestral allele "C " in double or single copy, may require a low dose of acenocoumarol to achieve the curative effect^32^. Based on these findings, we can hypothesize that the ancestral "C" allele is a protective allele for the Tunisian patients against hemorrhagic complications caused by an overdose of acenocoumarol. Our results oppose those of Ajmi et al. who declared the protective effect of rs7294 "T" allele against bleeding caused by acenocoumarol treatment^53^. This study was conducted on 246 Tunisian patients who originated from the Sahel region. This discrepancy could be due to Tunisian high genetic heterogeneity^30,54,55^. Thus, the T allele follows a south/north gradient from Africa to Europe, via Tunisia. This result highlights the need for genotyping Tunisian, Italian and other European patients for the variant rs7294 located on *VKORC1* gene in order to optimize the dose of acenocoumarol in to avoid the bleeding events in this susceptible populations.

The correlation of the genotype with the response to the drug and the knowledge of the frequencies of the risk allele associated with adverse drug effects in the different populations, improve the therapeutic results of the drugs. This has an important impact in the implementation of personalized medicine^56^. Our results show that the Tunisian population has a complex demographic history of migrations and gene flow involving the pharmacogenes, within Africa, Europe, and the Middle East. This genetic heterogeneity could pave the way for these populations to benefit from precision medicine. These studies provide several opportunities for detecting the association of host genotypes and ancestry in order to formulate specific country-level guidelines on drug reactions which could be useful and cost effective in clinical practice.

The study of the genetic distribution of variants in genes known to influence the inter-population variability in drug response could be greatly advantageous and helpful to implement a personalized medicine. It limits the additional costs of hospitalization of patients following side effects of drugs. Furthermore, assessing ADRs is critical for determining risk factors and maximizing the benefits of drug therapy. More information about prescribed drugs and their side effects will aid in reducing ADRs and ensuring patient safety.

## Populations and methods

### Selection of the pharmacogenes

A subset of very important pharmacogenes (VIP) involved in the ADR of many drugs comprising weight loss drugs, lipid-lowering, antihypertensive and oral antidiabetics agents, were extracted from the PharmGKB database (http://www.pharmgkb.org), which provides an overview of significant genes involved in drug metabolism or response. VIP genes were chosen after a through review of several sources, including the FDA (Food and Drug Administration) biomarker list, FDA-approved drug labels with pharmacogenetic information, and Clinical Pharmacogenetic Implementation Consortium (CPIC) nominations (https://cpicpgx.org.). A gene was also considered as VIP, if it was associated with a large number of variant annotations and a high level of clinical annotations. In addition, an extensive bibliographic search was used to select VIP variant comprising genetic polymorphisms located on VIP and having clinical evidence level association with ADR according to the PharmGKB database (Table 1).

### Genotyping data and quality control

Genotyping data was generated in a previously published Affymetrix Chip 6.0 genotyping array^57^ that investigated 135 healthy Tunisian individuals (participants were from the Capital Tunis and the coastal city of Monastir). The present study was designed and performed in accordance with relevant guidelines and regulations and according to ethical principles for medical research involving human subjects stated by the WMA Declaration of Helsinki. It was approved by the Ethics Committee of the Institut Pasteur (Tunis, Tunisia-Registration numbers IRB00005445, FWA00010074, and PV09/06, IRB# 0000000044). All participants provided written informed consent. Similarly, we used Illumina Chip genotyping data from 737 healthy Italian individuals originating from Northern, Central, Southern Italy and Sardinia. Genomics data was obtained from all participants within the framework of the study by Boattini et al.^58^ (protocol n. 85/2009/U/Tess approved by the Bologna S.Orsola-Malpighi University Hospital ethics committee). In addition, we used public genotyping data to perform comparisons. Indeed, the 1000 Genomes project populations were investigated including 2504 individuals from East Asia; [Han Chinese in Beijing, China (CHB), the Chinese population of metropolitan Denver, Colorado, USA (CHD), the Chinese Dai in Xishuangbanna, China (CDX) and Japanese in Tokyo, Japan; (JPT)], South Asia; [Gujarati INS from Houston, Texas (GIH),Punjabi from Lahore, Pakistan (PJL)], Bengali from Bangladesh (BEB), SL Tamils from the UK (STU), Indian Telugu from the UK (ITU)], Africa; [Gambian in Western Divisions in the Gambia (GWD), African Caribbean (ACB), African Ancestry in SW USA (ASW), Esan in Nigeria (ESN), Mende in Sierra Leone [MSL], Luhya in Webuye, Kenya (LWK)] and Europe; [Utah residents with Northern and Western European ancestry (CEU)],Tuscany (TSI) in Italy, Finnish in Finland (FIN), British in England and Scotland (GBR), Iberian Population in Spain (IBS)] (Supplementary Table 1).

Data were extracted from 1000 Genomes phase 3 release (http://www.1000genomes.org).

From the genotyping data, we extracted all variants located in the chromosomal region of the selected pharmacogenes. The genotyping data of the 135 Tunisians was merged with the genotyping data of the 737 healthy Italians, to identify the shared common variants between the two populations, using the software PLINK v2.

Variants were excluded if they were deviating from the Hardy–Weinberg equilibrium (HWE) (p-value < 10^–2^) and minor allele frequency (MAF) < 5 × 10^–2^. Individuals with missing genotype and genotyping quality less than 95%, were not included in the study.

### Statistical analysis

To assess the variance in pharmacogenes variants across different populations, we calculated Wright’s fixation index (Fst). The pairwise comparison of Fst values among Tunisian, Italian, and 1000 Genome project populations was carried out using the *Hierfstat* package in R. The χ^2^ statistical analysis test was used to compare the prevalence of risk alleles between the Tunisian, Italian and worldwide studied populations. The Bonforroni adjustment was applied to the significance level set at the 5% p-value threshold by the number of loci studied and the number of populations.

### Principal component analysis

The merge of genotyping data of all investigated populations was thinned with the PLINK software to perform the data pruning. We excluded variants in strong LD within a sliding window of 50 variants advanced by 10 SNPs at the same time, to infer cryptic population structure from genomic data. Cryptic population structure defines a population structure that is difficult to detect using visible characters but may be significant in genetic terms^59^. It is important to shape the false matches due to the probabilistic assignment of population. The pruned genotypic data was used to perform Principal Component Analysis “PCA”, based on Identity-By-Descent measures using the *SNPrelate* R package^60^. The *Scatter Plot* analysis was designed to study the correlation between the important variants. Allele frequency variation and conventional metrics such as fixation index (Fst) were conducted to have a robust population differentiation parameter^61^. The fixation index represents the percentage of total genetic variation at a given locus that differs between populations. This index is influenced by the frequency of minor alleles (MAF) and the size of the population sample^61^. The Manhattan plot analysis was conducted to depict a pairwise comparison of Fst values to evaluate the differentiation of variant frequencies between studied populations.

### Genetic structure

We estimated the admixture proportions by applying the unsupervised clustering algorithm implemented in the software ADMIXTURE on the pruned dataset^62^. We performed a series of admixture runs from K = 2 through 10 and we used the cross-validation (CV) error to identify the best predictive model. Next, we examined the variants with the highest level of differentiation between Tunisian, Italian and the other populations and drew the map representing the genetic distribution of the selected variants with R script.

## Conclusion

Finally, the present findings represent the first set of shared pharmacogenetic data for the Tunisian and Italian populations. These findings provide a valuable basis for further functional pharmacogenetic research, which is of great use in patient treatment in these populations. In addition, this study highlights the importance of the Tunisian-Italian clinical expertise exchange in disease management and personalized medicine implementation in the Mediterranean region. Our study revealed several shared VIP variants implicated in ADR between Tunisian and Italian populations. Thus, the identification of novel associated pharmacogenes in one population has a high probability of being replicated in other populations. The exchange of genetic and clinical expertise between these populations will greatly help to decrease treatment failure and minimize medication and hospitalization costs. Correlation between genotype and allelic frequencies of risk variants and their associated ADR would therefore improve drug outcomes and have a significant impact on the adoption of personalized medicine in people around the world. The establishment of a data bank or a registry including the pharmacogenetic data of specific populations, available to clinicians and the scientific community, is very useful. These registers allow the identification of populations which must be prudent concerning the consumption of certain drugs.

### Supplementary Information

Supplementary Information.

## Data Availability

The data generated and analyzed during the current study are available in the European Variation Archive (EVA) repository at EMBL-EBI under accession number PRJEB61743 (https://www.ebi.ac.uk/eva/?evastudy=PRJEB61743).
