# Supplementary material for: Genetic diversity of variants involved in drug response among Tunisian and Italian populations toward personalized medicine
Source: Sci Rep. 2024 Mar 10;14:5842. doi: 10.1038/s41598-024-55239-7 (PMC10925599; doi:10.1038/s41598-024-55239-7)
Supplement: Supplementary file 1 — Supplementary Information. [file 41598_2024_55239_MOESM1_ESM.pdf]

Genetic Diversity of variants involved in drug response among Tunisian and Italian populations toward personalized medicine

Authors: [1,2,3]Haifa Jmel, [4]Stefania Sarno, [4]Cristina Giuliani, [1,2]Wided Boukhalfa, [1,2]Sonia Abdelhak, [5]Donata Luiselli, [1,2,3]Rym Kefi*.

Affiliations

[1]Laboratory of Biomedical Genomics and Oncogenetics, Institut Pasteur de Tunis, Tunis, Tunisia.

[2]University of Tunis El Manar, Tunis, Tunisia.

[3]Genetic typing DNA Service Pasteur Institute, Institut Pasteur de Tunis, Tunis, Tunisia.

[4]Laboratory of Molecular Anthropology & Centre for Genome Biology, Department of Biological, Geological and Environmental Sciences (BiGeA), University of Bologna, Bologna, Italy.

[5]Laboratory of Ancient DNA (aDNALab), Department of Cultural Heritage (DBC), University of Bologna, Ravenna, Italy

*Corresponding Author; rym.kefi@pasteur.utm.tn, haifa.jmel@pasteur.utm.tn

Supplementary Table 1

Population	Population name	Superpopulation code	Individual's number
ACB	African Caribbean	AFR	96
ASW	African Ancestry SW	AFR	61
BEB	Bengali	SAS	86
C_ITA	Central Italian	EUR	403
CDX	Dai Chinese	EAS	93
CEU	CEPH	EUR	99
CHB	Han Chinese	EAS	103
CHS	Southern Han Chinese	EAS	105
CLM	Colombian	AMR	94
ESN	Esan	AFR	99
FIN	Finnish	EUR	99
GBR	British	EUR	91
GIH	Gujarati	SAS	103
GWD	Gambian Mandinka	AFR	113
IBS	Iberian,Spanish	EUR	107
ITU	Telugu	SAS	102
JPT	Japanese	EAS	104
KHV	Kinh Vietnamese	EAS	99
LWK	Luhya	AFR	99
MSL	Mende,Mende	AFR	85
MXL	Mexican Ancestry	AMR	64
N_ITA	North Italian	EUR	131
PEL	Peruvian	AMR	85
PJL	Punjabi	SAS	96
PUR	Puerto Rican	AMR	104
S_ITA	South Italian	EUR	156

Population	Population name	Superpopulation code	Individual's number
SARD	Sardinian	EUR	47
STU	Tamil	SAS	102
TN_MC	Tunisian from Monastir	TUN	71
TN_TC	Tunisian from Costal of	TUN	64
TSI	Toscani	EUR	107
YRI	Yoruba	AFR	108
**Total**			3376

Supplementary Table 1: Studied Populations
The table presents the studied populations. AFR: Sub-Saharan Africa, AMR: America, EAS: East Asiatic, EUR: Europe, SAS: South Asiatic

Supplementary Table 2: Fst Comparison among studied populations

Fst Comparison among Tunisian an Sardinian populations

Chr	Localisation	rs ID	Ho	Hs	Ht	Dst	Htp	Dstp	Fst	Fstp	Fis	Dest
1	65381861	rs12563017	0,1394	0,1775	0,1767	-0,0008	0,1760	-0,0016	**-0,0044**	**-0,0089**	0,2147	-0,0019
1	65389835	rs10889503	0,3143		0,3648	0,0030	0,3678	0,0059	**0,0081**	**0,0161**	0,1313	0,0093
1	65421058	rs4916014	0,3323	0,4200	0,4188	-0,0012	0,4176	-0,0024	**-0,0029**	**-0,0058**	0,2089	-0,0042
1	65427476	rs4915675		0,3688	0,3672	-0,0016	0,3657	-0,0031	**-0,0043**	**-0,0086**	0,1726	-0,0050
1	65516055	rs6588109	0,3128	0,3632	0,3665	0,0033	0,3697	0,0065	**0,0089**	**0,0176**	0,1387	0,0102
1	65557876	rs6699671	0,2253	0,2157	0,2180	0,0023	0,2203	0,0046	**0,0105**	**0,0207**	-0,0443	0,0058
1	65583858	rs11208591	0,4889	0,4930	0,4929	-0,0001	0,4929	-0,0002	**-0,0002**	**-0,0003**	0,0084	-0,0003
1	65619880	rs10789171	0,3596	0,3478	0,3469	-0,0009	0,3459	-0,0019	**-0,0027**	**-0,0055**	-0,0339	-0,0029
1	65658412	rs6677316	0,4325	0,3884	0,3872	-0,0012	0,3861	-0,0023	**-0,0030**	**-0,0061**	-0,1135	-0,0038
3	12286720	rs9850825	0,5107	0,4837	0,4918	0,0082	0,5000	0,0163	**0,0166**	**0,0327**	-0,0560	0,0317
3	12302462	rs9878908	0,1528	0,1725	0,1719	-0,0007	0,1712	-0,0014	**-0,0040**	**-0,0080**	0,1143	-0,0017
3	12393125	rs1801282	0,0810	0,0841	0,0848	0,0007	0,0856	0,0015	**0,0087**	**0,0173**	0,0372	0,0016
3	12402474	rs1373641	0,4405	0,4360	0,4449	0,0090	0,4539	0,0179	**0,0201**	**0,0395**	-0,0104	0,0318
3	12475088	rs7626560	0,3503	0,3333	0,3344	0,0011	0,3355	0,0022	**0,0033**	**0,0066**	-0,0511	0,0033
3	151007310	rs9863983	0,2120	0,2227	0,2257	0,0030	0,2287	0,0060	**0,0134**	**0,0264**	0,0478	0,0078
3	151041513	rs3971191	0,1152	0,1080	0,1090	0,0010	0,1099	0,0019	**0,0088**	**0,0174**	-0,0670	0,0021
3	151053898	rs7644001	0,4478	0,4723	0,4739	0,0016	0,4755	0,0032	**0,0034**	**0,0068**	0,0520	0,0061
3	151090963	rs9859538	0,4847	0,4896	0,5003	0,0107	0,5111	0,0215	**0,0215**	**0,0420**	0,0100	0,0421
3	151112568	rs3732768	0,2719	0,3374	0,3358	-0,0016	0,3343	-0,0031	**-0,0046**	**-0,0093**	0,1942	-0,0047
3	151128895	rs10935844	0,4472	0,4997	0,4978	-0,0019	0,4959	-0,0038	**-0,0038**	**-0,0076**	0,1050	-0,0075
3	151147968	rs6772196	0,1152	0,1267	0,1290	0,0023	0,1312	0,0045	**0,0175**	**0,0343**	0,0904	0,0052
5	51405600	rs12655411	0,4689	0,4169	0,4161	-0,0007	0,4154	-0,0015	**-0,0018**	**-0,0036**	-0,1248	-0,0025
5	51431680	rs10064799	0,4494	0,4400	0,4423	0,0024	0,4447	0,0048	**0,0054**	**0,0107**	-0,0215	0,0085
5	51505665	rs6865397	0,4663	0,4782	0,4782	0,0000	0,4782	0,0000	**0,0000**	**-0,0001**	0,0249	0,0000
5	51572584	rs4572960	0,4535	0,4672	0,4671	-0,0002	0,4669	-0,0003	**-0,0004**	**-0,0007**	0,0294	-0,0006
5	74616843	rs10474433	0,4452	0,4466	0,4549	0,0084	0,4633	0,0168	**0,0184**	**0,0362**	0,0030	0,0303
5	74620912	rs6878576	0,2555	0,2769	0,2772	0,0003	0,2775	0,0005	**0,0009**	**0,0019**	0,0775	0,0007
5	74625487	rs7703051	0,4036	0,4289	0,4273	-0,0016	0,4257	-0,0033	**-0,0038**	**-0,0077**	0,0590	-0,0057
5	74648603	rs12654264	0,4014	0,4313	0,5017	0,0703	0,5720	0,1407	**0,1402**	**0,2459**	0,0694	0,2474
5	74651084	rs3846662	0,4352	0,4744	0,4840	0,0096	0,4936	0,0192	**0,0199**	**0,0390**	0,0826	0,0366
5	74655726	rs3846663	0,3924	0,4305	0,4288	-0,0017	0,4271	-0,0034	**-0,0039**	**-0,0079**	0,0884	-0,0059
5	74656175	rs5909	0,1240	0,1303	0,1298	-0,0004	0,1294	-0,0009	**-0,0033**	**-0,0066**	0,0483	-0,0010
6	160496055	rs3777406	0,3333	0,3342	0,3568	0,0226	0,3794	0,0452	**0,0634**	**0,1192**	0,0027	0,0679
6	160517481	rs1803989	0,1358	0,1323	0,1335	0,0012	0,1347	0,0025	**0,0092**	**0,0183**	-0,0270	0,0028
6	160528057	rs7753051	0,3779	0,4018	0,4021	0,0004	0,4025	0,0007	**0,0009**	**0,0019**	0,0593	0,0012
6	160572866	rs622342	0,3769	0,4140	0,4249	0,0108	0,4357	0,0216	**0,0255**	**0,0497**	0,0897	0,0369
6	160578860	rs1564348	0,2559	0,2778	0,2768	-0,0010	0,2758	-0,0021	**-0,0037**	**-0,0075**	0,0789	-0,0029
6	160581374	rs651164	0,4790	0,4489	0,4545	0,0055	0,4600	0,0111	**0,0122**	**0,0241**	-0,0671	0,0201
6	160582340	rs9456505	0,1284	0,1271	0,1271	0,0000	0,1271	0,0000	**-0,0001**	**-0,0003**	-0,0098	0,0000
6	160635886	rs10945656	0,1950	0,2117	0,2112	-0,0005	0,2108	-0,0010	**-0,0023**	**-0,0046**	0,0792	-0,0012
6	160637239	rs596881	0,2005	0,2428	0,2442	0,0015	0,2457	0,0029	**0,0060**	**0,0119**	0,1741	0,0038
6	160672625	rs316013	0,3866	0,3697	0,3750	0,0053	0,3803	0,0106	**0,0141**	**0,0278**	-0,0458	0,0168
6	160681393	rs3127573	0,1911	0,1871	0,1864	-0,0007	0,1857	-0,0013	**-0,0035**	**-0,0071**	-0,0214	-0,0016
6	160682897	rs7757997	0,1435	0,1401	0,1463	0,0062	0,1525	0,0124	**0,0425**	**0,0815**	-0,0245	0,0144
6	160687866	rs316030	0,4888	0,4227	0,4229	0,0002	0,4231	0,0005	**0,0006**	**0,0011**	-0,1566	0,0008
6	160699534	rs2619276	0,3390	0,3116	0,3108	-0,0008	0,3100	-0,0016	**-0,0026**	**-0,0051**	-0,0879	-0,0023
7	87103670	rs2888611	0,3611	0,3362	0,3419	0,0057	0,3476	0,0113	**0,0166**	**0,0326**	-0,0739	0,0171
7	87154646	rs10225473	0,2883	0,3143	0,3195	0,0052	0,3247	0,0104	**0,0163**	**0,0321**	0,0825	0,0152
7	87163016	rs11760837	0,2611	0,2949	0,3034	0,0085	0,3119	0,0171	**0,0281**	**0,0547**	0,1145	0,0242
7	87179143	rs2235033	0,5141	0,4953	0,4951	-0,0002	0,4950	-0,0003	**-0,0003**	**-0,0007**	-0,0380	-0,0007
7	87179809	rs2229109	0,1041	0,1233	0,1245	0,0012	0,1257	0,0024	**0,0097**	**0,0193**	0,1555	0,0028
7	87180198	rs10276036	0,4878	0,4721	0,4709	-0,0012	0,4696	-0,0025	**-0,0026**	**-0,0053**	-0,0333	-0,0047
7	87183354	rs1922240	0,4377	0,4329	0,4328	0,0000	0,4328	-0,0001	**-0,0001**	**-0,0002**	-0,0111	-0,0001
7	87201482	rs10260862	0,3781	0,3982	0,4005	0,0023	0,4028	0,0046	**0,0058**	**0,0114**	0,0507	0,0077
7	87278760	rs10267099	0,3930	0,3822	0,3808	-0,0013	0,3795	-0,0027	**-0,0035**	**-0,0070**	-0,0283	-0,0043
7	99207876	rs7792939	0,1165	0,1422	0,1417	-0,0005	0,1413	-0,0009	**-0,0032**	**-0,0064**	0,1808	-0,0011
10	96581094	rs10786172	0,3406	0,3879	0,3869	-0,0010	0,3858	-0,0021	**-0,0027**	**-0,0054**	0,1220	-0,0034
10	114711983	rs7094463	0,4985	0,4854	0,4874	0,0020	0,4894	0,0040	**0,0041**	**0,0081**	-0,0270	0,0077
10	114732906	rs7901275	0,4580	0,4912	0,4921	0,0010	0,4931	0,0020	**0,0020**	**0,0040**	0,0674	0,0038
10	114754088	rs7901695	0,4553	0,4630	0,4620	-0,0010	0,4610	-0,0020	**-0,0021**	**-0,0043**	0,0165	-0,0037
10	114756041	rs4506565	0,4598	0,4668	0,4655	-0,0013	0,4642	-0,0026	**-0,0028**	**-0,0056**	0,0151	-0,0049
10	114767771	rs4132670	0,4674	0,4802	0,4811	0,0009	0,4821	0,0018	**0,0019**	**0,0038**	0,0266	0,0035
10	114788815	rs12243326	0,4458	0,4639	0,4622	-0,0017	0,4605	-0,0034	**-0,0037**	**-0,0075**	0,0392	-0,0064
10	114821249	rs11196212	0,4680	0,4880	0,5015	0,0135	0,5150	0,0271	**0,0270**	**0,0525**	0,0409	0,0528
10	114855397	rs11196224	0,4115	0,4462	0,4615	0,0153	0,4768	0,0305	**0,0331**	**0,0640**	0,0779	0,0551

Chr	Localisation	rs ID	Ho	Hs	Ht	Dst	Htp	Dstp	Fst	Fstp	Fis	Dest
10	114859463	rs7085532	0,4447	0,4555	0,4665	0,0109	0,4774	0,0219	**0,0235**	**0,0459**	0,0238	0,0402
10	114898093	rs3814573	0,4609	0,4839	0,4824	-0,0015	0,4808	-0,0031	**-0,0032**	**-0,0064**	0,0476	-0,0060
10	114912534	rs1555485	0,1950	0,1767	0,1829	0,0062	0,1891	0,0124	**0,0338**	**0,0654**	-0,1036	0,0150
11	2528003	rs11023096	0,2870	0,3113	0,3101	-0,0012	0,3089	-0,0024	**-0,0039**	**-0,0078**	0,0781	-0,0035
11	2528233	rs4929992	0,4542	0,4926	0,4909	-0,0017	0,4892	-0,0034	**-0,0035**	**-0,0070**	0,0780	-0,0067
11	2550730	rs179429	0,3184	0,3440	0,3526	0,0086	0,3612	0,0173	**0,0245**	**0,0478**	0,0744	0,0263
11	2553703	rs179435	0,3622	0,4255	0,4240	-0,0015	0,4225	-0,0030	**-0,0036**	**-0,0072**	0,1488	-0,0053
11	2595287	rs2283171	0,4303	0,4509	0,4500	-0,0009	0,4491	-0,0018	**-0,0020**	**-0,0039**	0,0455	-0,0032
11	2617782	rs1116714	0,3338	0,3377	0,3522	0,0145	0,3667	0,0290	**0,0412**	**0,0792**	0,0116	0,0439
11	2633152	rs10766212	0,5065	0,5034	0,5017	-0,0017	0,5000	-0,0034	**-0,0034**	**-0,0069**	-0,0061	-0,0069
11	2635797	rs2106467	0,4882	0,4916	0,4935	0,0019	0,4954	0,0039	**0,0039**	**0,0078**	0,0069	0,0076
11	2673575	rs6578283	0,5241	0,4681	0,4672	-0,0008	0,4664	-0,0017	**-0,0018**	**-0,0036**	-0,1197	-0,0031
11	2750703	rs170786	0,4498	0,4557	0,4557	0,0001	0,4558	0,0001	**0,0001**	**0,0003**	0,0129	0,0002
11	2776448	rs11023996	0,1504	0,1597	0,1598	0,0001	0,1599	0,0002	**0,0007**	**0,0015**	0,0582	0,0003
11	2782648	rs548566	0,2786	0,2750	0,2740	-0,0010	0,2730	-0,0019	**-0,0035**	**-0,0071**	-0,0132	-0,0027
11	2821065	rs163171	0,3715	0,3743	0,3731	-0,0012	0,3718	-0,0025	**-0,0033**	**-0,0066**	0,0075	-0,0039
11	2837625	rs233446	0,3214	0,3660	0,3703	0,0043	0,3746	0,0087	**0,0117**	**0,0232**	0,1219	0,0137
11	2850782	rs234852	0,3277	0,3591	0,3884	0,0294	0,4178	0,0587	**0,0756**	**0,1405**	0,0874	0,0916
11	2895800	rs3987740	0,4771	0,4590	0,4575	-0,0016	0,4559	-0,0032	**-0,0035**	**-0,0069**	-0,0393	-0,0058
11	17393644	rs12791318	0,3378	0,3139	0,3158	0,0019	0,3177	0,0038	**0,0060**	**0,0119**	-0,0760	0,0055
11	17405333	rs10832785	0,3946	0,4533	0,4714	0,0181	0,4894	0,0362	**0,0384**	**0,0739**	0,1294	0,0662
11	17408025	rs2285676	0,4303	0,4618	0,4749	0,0131	0,4879	0,0261	**0,0275**	**0,0536**	0,0682	0,0486
11	17408630	rs5215	0,3174	0,3551	0,3747	0,0196	0,3944	0,0393	**0,0524**	**0,0996**	0,1061	0,0609
11	17408831	rs1800467	0,0858	0,0818	0,0824	0,0006	0,0830	0,0012	**0,0071**	**0,0141**	-0,0485	0,0013
11	17438890	rs2074315	0,2335	0,2441	0,2479	0,0038	0,2517	0,0076	**0,0153**	**0,0301**	0,0434	0,0100
11	17441828	rs4757517	0,3073	0,3349	0,3400	0,0051	0,3450	0,0101	**0,0149**	**0,0293**	0,0824	0,0152
11	17496516	rs1048099	0,4066	0,4510	0,4535	0,0026	0,4561	0,0052	**0,0057**	**0,0113**	0,0983	0,0094
11	17510419	rs11603988	0,1420	0,1327	0,1323	-0,0003	0,1320	-0,0007	**-0,0026**	**-0,0053**	-0,0699	-0,0008
11	17510565	rs4757527	0,3525	0,3993	0,4000	0,0007	0,4007	0,0014	**0,0018**	**0,0035**	0,1171	0,0023
11	17530484	rs7104083	0,4317	0,4946	0,4936	-0,0010	0,4926	-0,0020	**-0,0021**	**-0,0041**	0,1271	-0,0040
11	17532597	rs1076311	0,4452	0,4936	0,4937	0,0001	0,4939	0,0003	**0,0003**	**0,0005**	0,0981	0,0005
11	17542649	rs2041032	0,4871	0,5019	0,5014	-0,0005	0,5009	-0,0010	**-0,0010**	**-0,0020**	0,0294	-0,0020
11	108097333	rs228591	0,3845	0,4022	0,4122	0,0100	0,4223	0,0200	**0,0243**	**0,0475**	0,0440	0,0335
11	108268286	rs7931930	0,3977	0,4251	0,4507	0,0256	0,4763	0,0511	**0,0567**	**0,1074**	0,0646	0,0890
11	108283161	rs11212617	0,3689	0,4124	0,4229	0,0106	0,4335	0,0212	**0,0250**	**0,0488**	0,1055	0,0360
16	31102321	rs7294	0,3137	0,3876	0,3899	0,0024	0,3923	0,0047	**0,0060**	**0,0120**	0,1907	0,0077
17	19447016	rs2440155	0,2869	0,2755	0,2774	0,0019	0,2794	0,0039	**0,0070**	**0,0139**	-0,0416	0,0054
17	19459537	rs2244280	0,2749	0,3112	0,3108	-0,0004	0,3104	-0,0007	**-0,0012**	**-0,0024**	0,1165	-0,0011
17	19484951	rs2453594	0,2687	0,3160	0,3150	-0,0010	0,3141	-0,0019	**-0,0031**	**-0,0062**	0,1496	-0,0028
17	19622643	rs11656096	0,2254	0,3218	0,3445	0,0227	0,3672	0,0454	**0,0658**	**0,1235**	0,2996	0,0669
17	19642952	rs2228100	0,3631	0,4212	0,4972	0,0760	0,5732	0,1520	**0,1528**	**0,2651**	0,1380	0,2626
17	19645938	rs887241	0,4164	0,4665	0,4790	0,0124	0,4914	0,0248	**0,0259**	**0,0505**	0,1074	0,0465
22	42152988	rs17377643	0,4276	0,4072	0,4074	0,0002	0,4076	0,0003	**0,0004**	**0,0008**	-0,0500	0,0006
22	42178441	rs126092	0,3441	0,4114	0,4103	-0,0011	0,4092	-0,0022	**-0,0026**	**-0,0053**	0,1636	-0,0037
22	46235677	rs1023470	0,3434	0,3501	0,3535	0,0034	0,3569	0,0068	**0,0096**	**0,0190**	0,0190	0,0105
22	46238069	rs8141212	0,5030	0,4806	0,4842	0,0035	0,4877	0,0071	**0,0073**	**0,0145**	-0,0466	0,0137
22	46525794	rs6007919	0,3703	0,4149	0,4171	0,0022	0,4194	0,0044	**0,0053**	**0,0106**	0,1076	0,0076
22	46629479	rs4253776	0,2850	0,2959	0,2951	-0,0008	0,2943	-0,0016	**-0,0027**	**-0,0054**	0,0368	-0,0022
22	46637254	rs9626814	0,2736	0,2828	0,2820	-0,0008	0,2812	-0,0016	**-0,0028**	**-0,0056**	0,0326	-0,0022
22	46643774	rs16995069	0,1033	0,1107	0,1118	0,0011	0,1129	0,0021	**0,0095**	**0,0188**	0,0668	0,0024
22	46670394	rs6007761	0,2600	0,2765	0,2755	-0,0010	0,2745	-0,0020	**-0,0037**	**-0,0074**	0,0600	-0,0028

**$overall**	**Ho**	**Hs**	**Ht**	**Dst**	**Htp**	**Dstp**	**Fst**	**Fstp**	**Fis**	**Dest**
0,3446	0,3602	0,3647	0,0044	0,3691	0,0089	0,0122	**0,0240**	**0,0433**	0,0139	

$FST 0,0237

$FIS 0,0631

Supplementary Table 2 next

Fst Comparison among Tunisian an North Italian populations

Chr	Localisation	rs ID	Ho	Hs	Ht	Dst	Htp	Dstp	Fst	Fstp	Fis	Dest
1	65381861	rs12563017	0,1200	0,1671	0,1668	-0,0003	0,1665	-0,0006	**-0,0018**	**-0,0036**	0,2820	-0,0007
1	65389835	rs10889503	0,2837		0,3276	0,0121	0,3397	0,0242	**0,0369**	**0,0713**	0,1006	0,0354
1	65421058	rs4916014	0,3163	0,3815	0,3866	0,0050	0,3916	0,0100	**0,0130**	**0,0256**	0,1709	0,0162
1	65427476	rs4915675		0,3395	0,3402	0,0006	0,3408	0,0013	**0,0019**	**0,0038**	0,0772	0,0020
1	65516055	rs6588109	0,3569	0,3897	0,3900	0,0004	0,3904	0,0008	**0,0010**	**0,0019**	0,0840	0,0012
1	65557876	rs6699671	0,2402	0,2272	0,2287	0,0016	0,2303	0,0031	**0,0069**	**0,0136**	-0,0572	0,0041
1	65583858	rs11208591	0,5204	0,4927	0,4931	0,0004	0,4934	0,0008	**0,0008**	**0,0016**	-0,0563	0,0016
1	65619880	rs10789171	0,3453	0,3521	0,3520	-0,0001	0,3518	-0,0002	**-0,0003**	**-0,0007**	0,0193	-0,0004
1	65658412	rs6677316	0,4046	0,4033	0,4028	-0,0005	0,4022	-0,0010	**-0,0013**	**-0,0025**	-0,0033	-0,0017
3	12286720	rs9850825	0,5021	0,4806	0,4850	0,0043	0,4893	0,0087	**0,0089**	**0,0177**	-0,0447	0,0167
3	12302462	rs9878908	0,2196	0,2277	0,2304	0,0026	0,2330	0,0053	**0,0114**	**0,0226**	0,0358	0,0068
3	12393125	rs1801282	0,1437	0,1464	0,1464	0,0000	0,1465	0,0000	**0,0001**	**0,0002**	0,0188	0,0000
3	12402474	rs1373641	0,4104	0,4269	0,4322	0,0053	0,4375	0,0106	**0,0123**	**0,0243**	0,0386	0,0186
3	12475088	rs7626560	0,3008	0,2969	0,2964	-0,0005	0,2958	-0,0011	**-0,0018**	**-0,0036**	-0,0131	-0,0015
3	151007310	rs9863983	0,2758	0,2915	0,2909	-0,0006	0,2904	-0,0011	**-0,0020**	**-0,0039**	0,0540	-0,0016
3	151041513	rs3971191	0,1940	0,1872	0,1877	0,0006	0,1883	0,0011	**0,0030**	**0,0060**	-0,0366	0,0014
3	151053898	rs7644001	0,4599	0,4769	0,4778	0,0009	0,4788	0,0019	**0,0020**	**0,0039**	0,0357	0,0036
3	151090963	rs9859538	0,4652	0,4879	0,4995	0,0116	0,5111	0,0232	**0,0233**	**0,0455**	0,0465	0,0454
3	151112568	rs3732768	0,2893	0,3079	0,3082	0,0002	0,3084	0,0005	**0,0008**	**0,0016**	0,0606	0,0007
3	151128895	rs10935844	0,5191	0,4989	0,4980	-0,0009	0,4971	-0,0018	**-0,0018**	**-0,0037**	-0,0404	-0,0036
3	151147968	rs6772196	0,1444	0,1595	0,1598	0,0004	0,1602	0,0007	**0,0023**	**0,0046**	0,0944	0,0009
5	51405600	rs12655411	0,4257	0,4389	0,4384	-0,0005	0,4378	-0,0011	**-0,0012**	**-0,0025**	0,0301	-0,0019
5	51431680	rs10064799	0,4681	0,4501	0,4602	0,0101	0,4702	0,0202	**0,0219**	**0,0429**	-0,0400	0,0367
5	51505665	rs6865397	0,5185	0,4919	0,4911	-0,0007	0,4904	-0,0015	**-0,0015**	**-0,0030**	-0,0542	-0,0029
5	51572584	rs4572960	0,5461	0,4802	0,4794	-0,0008	0,4786	-0,0016	**-0,0016**	**-0,0033**	-0,1372	-0,0030
5	74616843	rs10474433	0,5056	0,4771	0,4776	0,0005	0,4781	0,0010	**0,0010**	**0,0020**	-0,0598	0,0018
5	74620912	rs6878576	0,2630	0,2632	0,2632	0,0000	0,2632	0,0000	**0,0000**	**0,0000**	0,0007	0,0000
5	74625487	rs7703051	0,4420	0,4527	0,4556	0,0029	0,4585	0,0058	**0,0064**	**0,0128**	0,0237	0,0107
5	74648603	rs12654264	0,4397	0,4550	0,4976	0,0426	0,5402	0,0851	**0,0856**	**0,1576**	0,0337	0,1562
5	74651084	rs3846662	0,4774	0,4983	0,4981	-0,0002	0,4978	-0,0005	**-0,0005**	**-0,0010**	0,0421	-0,0010
5	74655726	rs3846663	0,4308	0,4542	0,4567	0,0026	0,4593	0,0052	**0,0056**	**0,0112**	0,0515	0,0094
5	74656175	rs5909	0,1327	0,1565	0,1564	-0,0001	0,1563	-0,0003	**-0,0009**	**-0,0018**	0,1525	-0,0003
6	160496055	rs3777406	0,2405	0,2651	0,2672	0,0021	0,2692	0,0042	**0,0078**	**0,0154**	0,0929	0,0057
6	160517481	rs1803989	0,1315	0,1418	0,1426	0,0008	0,1434	0,0017	**0,0058**	**0,0116**	0,0727	0,0019
6	160528057	rs7753051	0,3225	0,3676	0,3668	-0,0008	0,3659	-0,0016	**-0,0022**	**-0,0045**	0,1227	-0,0026
6	160572866	rs622342	0,3410	0,4001	0,4057	0,0055	0,4112	0,0111	**0,0136**	**0,0269**	0,1477	0,0185
6	160578860	rs1564348	0,2572	0,2693	0,2690	-0,0003	0,2687	-0,0006	**-0,0012**	**-0,0023**	0,0449	-0,0008
6	160581374	rs651164	0,4312	0,4551	0,4593	0,0042	0,4634	0,0083	**0,0091**	**0,0180**	0,0525	0,0153
6	160582340	rs9456505	0,1401	0,1370	0,1370	-0,0001	0,1369	-0,0002	**-0,0007**	**-0,0014**	-0,0221	-0,0002
6	160635886	rs10945656	0,1976	0,2261	0,2256	-0,0005	0,2251	-0,0009	**-0,0020**	**-0,0041**	0,1257	-0,0012
6	160637239	rs596881	0,1803	0,2072	0,2130	0,0058	0,2188	0,0117	**0,0274**	**0,0533**	0,1299	0,0147
6	160672625	rs316013	0,3729	0,3705	0,3760	0,0055	0,3815	0,0110	**0,0146**	**0,0288**	-0,0065	0,0175
6	160681393	rs3127573	0,1725	0,1899	0,1895	-0,0004	0,1892	-0,0007	**-0,0019**	**-0,0038**	0,0918	-0,0009
6	160682897	rs7757997	0,1604	0,1555	0,1601	0,0046	0,1647	0,0092	**0,0288**	**0,0559**	-0,0312	0,0109
6	160687866	rs316030	0,4421	0,4358	0,4403	0,0045	0,4448	0,0091	**0,0103**	**0,0204**	-0,0144	0,0161
6	160699534	rs2619276	0,3588	0,3530	0,3538	0,0008	0,3546	0,0015	**0,0022**	**0,0043**	-0,0163	0,0024
7	87103670	rs2888611	0,3427	0,3484	0,3524	0,0040	0,3564	0,0080	**0,0114**	**0,0225**	0,0164	0,0123
7	87154646	rs10225473	0,2288	0,2439	0,2434	-0,0005	0,2429	-0,0010	**-0,0021**	**-0,0041**	0,0620	-0,0013
7	87163016	rs11760837	0,2054	0,2273	0,2272	-0,0001	0,2272	-0,0001	**-0,0003**	**-0,0006**	0,0963	-0,0002
7	87179143	rs2235033	0,5001	0,5006	0,5009	0,0002	0,5011	0,0005	**0,0005**	**0,0010**	0,0012	0,0010
7	87179809	rs2229109	0,0945	0,0967	0,0969	0,0002	0,0971	0,0004	**0,0021**	**0,0042**	0,0227	0,0004
7	87180198	rs10276036	0,5057	0,4885	0,4905	0,0020	0,4925	0,0039	**0,0040**	**0,0079**	-0,0351	0,0076
7	87183354	rs1922240	0,4509	0,4388	0,4387	-0,0001	0,4385	-0,0003	**-0,0003**	**-0,0006**	-0,0276	-0,0005
7	87201482	rs10260862	0,3103	0,3384	0,3382	-0,0002	0,3380	-0,0004	**-0,0005**	**-0,0011**	0,0829	-0,0006
7	87278760	rs10267099	0,3809	0,3811	0,3804	-0,0007	0,3797	-0,0014	**-0,0019**	**-0,0038**	0,0006	-0,0023
7	99207876	rs7792939	0,1671	0,1767	0,1783	0,0016	0,1799	0,0032	**0,0088**	**0,0175**	0,0543	0,0038
10	96581094	rs10786172	0,3536	0,3784	0,3777	-0,0006	0,3771	-0,0013	**-0,0017**	**-0,0034**	0,0656	-0,0021
10	114711983	rs7094463	0,4637	0,4904	0,4911	0,0007	0,4917	0,0013	**0,0014**	**0,0027**	0,0544	0,0026
10	114732906	rs7901275	0,4645	0,4894	0,4914	0,0020	0,4934	0,0040	**0,0041**	**0,0081**	0,0510	0,0078
10	114754088	rs7901695	0,4481	0,4658	0,4652	-0,0006	0,4645	-0,0013	**-0,0014**	**-0,0028**	0,0381	-0,0024
10	114756041	rs4506565	0,4419	0,4664	0,4658	-0,0006	0,4651	-0,0013	**-0,0014**	**-0,0027**	0,0526	-0,0024
10	114767771	rs4132670	0,4427	0,4756	0,4781	0,0026	0,4807	0,0051	**0,0053**	**0,0106**	0,0690	0,0097
10	114788815	rs12243326	0,4173	0,4532	0,4526	-0,0006	0,4520	-0,0012	**-0,0014**	**-0,0027**	0,0794	-0,0022
10	114821249	rs11196212	0,4760	0,4915	0,4973	0,0058	0,5031	0,0115	**0,0116**	**0,0230**	0,0315	0,0227
10	114855397	rs11196224	0,4847	0,4711	0,4772	0,0061	0,4833	0,0123	**0,0128**	**0,0254**	-0,0290	0,0232

Supplementary Table 2 next

Chr	Localisation	rs ID	Ho	Hs	Ht	Dst	Htp	Dstp	Fst	Fstp	Fis	Dest
10	114859463	rs7085532	0,4451	0,4356	0,4370	0,0014	0,4385	0,0029	**0,0033**	**0,0066**	-0,0219	0,0051
10	114898093	rs3814573	0,4261	0,4585	0,4595	0,0010	0,4605	0,0019	**0,0021**	**0,0042**	0,0706	0,0035
10	114912534	rs1555485	0,1538	0,1578	0,1616	0,0038	0,1653	0,0075	**0,0233**	**0,0455**	0,0256	0,0089
11	2528003	rs11023096	0,2564	0,2819	0,2822	0,0003	0,2825	0,0006	**0,0010**	**0,0020**	0,0907	0,0008
11	2528233	rs4929992	0,4827	0,4938	0,4995	0,0057	0,5052	0,0114	**0,0114**	**0,0226**	0,0225	0,0226
11	2550730	rs179429	0,3024	0,3057	0,3072	0,0015	0,3087	0,0030	**0,0049**	**0,0098**	0,0106	0,0043
11	2553703	rs179435	0,3683	0,3842	0,3853	0,0011	0,3864	0,0022	**0,0028**	**0,0057**	0,0414	0,0036
11	2595287	rs2283171	0,4139	0,4362	0,4380	0,0018	0,4398	0,0036	**0,0041**	**0,0082**	0,0513	0,0064
11	2617782	rs1116714	0,3367	0,3547	0,3655	0,0108	0,3763	0,0216	**0,0295**	**0,0574**	0,0506	0,0335
11	2633152	rs10766212	0,4987	0,4974	0,4997	0,0022	0,5019	0,0045	**0,0045**	**0,0090**	-0,0025	0,0089
11	2635797	rs2106467	0,4894	0,4850	0,4847	-0,0003	0,4844	-0,0006	**-0,0006**	**-0,0012**	-0,0090	-0,0011
11	2673575	rs6578283	0,4735	0,4751	0,4742	-0,0009	0,4733	-0,0018	**-0,0019**	**-0,0038**	0,0034	-0,0034
11	2750703	rs170786	0,4660	0,4488	0,4506	0,0018	0,4524	0,0036	**0,0040**	**0,0080**	-0,0384	0,0066
11	2776448	rs11023996	0,1394	0,1685	0,1693	0,0009	0,1702	0,0018	**0,0052**	**0,0103**	0,1724	0,0021
11	2782648	rs548566	0,2755	0,2921	0,2917	-0,0004	0,2913	-0,0008	**-0,0013**	**-0,0027**	0,0568	-0,0011
11	2821065	rs163171	0,3381	0,3615	0,3608	-0,0007	0,3601	-0,0015	**-0,0020**	**-0,0041**	0,0647	-0,0023
11	2837625	rs233446	0,3267	0,3474	0,3492	0,0017	0,3509	0,0034	**0,0049**	**0,0098**	0,0596	0,0053
11	2850782	rs234852	0,3726	0,4450	0,4486	0,0037	0,4523	0,0073	**0,0081**	**0,0161**	0,1627	0,0132
11	2895800	rs3987740	0,4592	0,4432	0,4433	0,0001	0,4435	0,0002	**0,0002**	**0,0005**	-0,0360	0,0004
11	17393644	rs12791318	0,2929	0,3104	0,3123	0,0019	0,3142	0,0039	**0,0062**	**0,0123**	0,0562	0,0056
11	17405333	rs10832785	0,4534	0,4691	0,4803	0,0112	0,4915	0,0224	**0,0233**	**0,0455**	0,0335	0,0422
11	17408025	rs2285676	0,4793	0,4700	0,4798	0,0097	0,4895	0,0195	**0,0203**	**0,0398**	-0,0197	0,0368
11	17408630	rs5215	0,3056	0,3611	0,3873	0,0261	0,4134	0,0523	**0,0675**	**0,1264**	0,1539	0,0818
11	17408831	rs1800467	0,0906	0,1052	0,1073	0,0022	0,1095	0,0044	**0,0203**	**0,0398**	0,1381	0,0049
11	17438890	rs2074315	0,3286	0,3493	0,3508	0,0015	0,3523	0,0030	**0,0043**	**0,0085**	0,0592	0,0046
11	17441828	rs4757517	0,4172	0,4082	0,4077	-0,0005	0,4072	-0,0009	**-0,0012**	**-0,0023**	-0,0222	-0,0016
11	17496516	rs1048099	0,4807	0,4884	0,4896	0,0013	0,4909	0,0025	**0,0026**	**0,0051**	0,0157	0,0049
11	17510419	rs11603988	0,2422	0,2099	0,2132	0,0034	0,2166	0,0067	**0,0157**	**0,0310**	-0,1541	0,0085
11	17510565	rs4757527	0,3259	0,3689	0,3681	-0,0008	0,3673	-0,0015	**-0,0021**	**-0,0042**	0,1164	-0,0025
11	17530484	rs7104083	0,4870	0,4938	0,4949	0,0011	0,4960	0,0022	**0,0022**	**0,0045**	0,0138	0,0044
11	17532597	rs1076311	0,4859	0,4922	0,4943	0,0020	0,4963	0,0041	**0,0041**	**0,0082**	0,0128	0,0080
11	17542649	rs2041032	0,4357	0,4990	0,4979	-0,0011	0,4968	-0,0021	**-0,0021**	**-0,0043**	0,1268	-0,0042
11	108097333	rs228591	0,4395	0,4578	0,4572	-0,0006	0,4566	-0,0012	**-0,0013**	**-0,0026**	0,0400	-0,0022
11	108268286	rs7931930	0,4633	0,4745	0,4804	0,0059	0,4863	0,0117	**0,0122**	**0,0241**	0,0237	0,0223
11	108283161	rs11212617	0,4345	0,4617	0,4616	-0,0001	0,4615	-0,0003	**-0,0003**	**-0,0006**	0,0590	-0,0005
16	31102321	rs7294	0,3618	0,4048	0,4148	0,0100	0,4249	0,0201	**0,0242**	**0,0473**	0,1061	0,0337
17	19447016	rs2440155	0,2890	0,2980	0,2983	0,0004	0,2987	0,0007	**0,0012**	**0,0024**	0,0300	0,0010
17	19459537	rs2244280	0,2748	0,3160	0,3159	-0,0001	0,3157	-0,0003	**-0,0004**	**-0,0009**	0,1302	-0,0004
17	19484951	rs2453594	0,2793	0,3280	0,3273	-0,0007	0,3266	-0,0015	**-0,0023**	**-0,0046**	0,1486	-0,0022
17	19622643	rs11656096	0,3161	0,3291	0,3595	0,0303	0,3898	0,0607	**0,0844**	**0,1556**	0,0395	0,0904
17	19642952	rs2228100	0,3838	0,4287	0,4981	0,0693	0,5674	0,1386	**0,1392**	**0,2443**	0,1049	0,2427
17	19645938	rs887241	0,3937	0,4490	0,4693	0,0204	0,4897	0,0407	**0,0434**	**0,0831**	0,1231	0,0739
22	42152988	rs17377643	0,4553	0,4614	0,4677	0,0063	0,4740	0,0127	**0,0136**	**0,0268**	0,0132	0,0236
22	42178441	rs126092	0,3121	0,3565	0,3646	0,0081	0,3727	0,0163	**0,0223**	**0,0436**	0,1244	0,0253
22	46235677	rs1023470	0,2926	0,3211	0,3213	0,0002	0,3215	0,0004	**0,0006**	**0,0012**	0,0889	0,0006
22	46238069	rs8141212	0,4584	0,4757	0,4774	0,0017	0,4791	0,0033	**0,0035**	**0,0069**	0,0364	0,0063
22	46525794	rs6007919	0,3878	0,4334	0,4334	0,0001	0,4335	0,0001	**0,0001**	**0,0003**	0,1052	0,0002
22	46629479	rs4253776	0,2102	0,2443	0,2449	0,0006	0,2455	0,0013	**0,0026**	**0,0053**	0,1393	0,0017
22	46637254	rs9626814	0,1843	0,2350	0,2354	0,0004	0,2357	0,0007	**0,0015**	**0,0030**	0,2157	0,0009
22	46643774	rs16995069	0,1249	0,1301	0,1304	0,0003	0,1307	0,0006	**0,0022**	**0,0044**	0,0401	0,0007
22	46670394	rs6007761	0,2233	0,2767	0,2761	-0,0006	0,2756	-0,0011	**-0,0021**	**-0,0042**	0,1929	-0,0016

	Ho	Hs	Ht	Dst	Htp	Dstp	Fst	Fstp	Fis	Dest
**$overall**	0,3495	0,3642	0,3677	0,0034	0,3711	0,0069	**0,0093**	**0,0185**	0,0406	0,0108

$FST 0,0185

$FIS 0,0398

Supplementary Table 2 next

Fst Comparison among Tunisian and Central Italian populations

Chr	Localisation	rs ID	Ho	Hs	Ht	Dst	Htp	Dstp	Fst	Fstp	Fis	Dest
1	65381861	rs12563017	0,1379	0,1872	0,1870	-0,0003	0,1867	-0,0005	**-0,0014**	**-0,0028**	0,2633	-0,0006
1	65389835	rs10889503	0,3131		0,3577	0,0051	0,3628	0,0102	**0,0143**	**0,0282**	0,1121	0,0158
1	65421058	rs4916014	0,3467	0,4181	0,4181	0,0000	0,4181	-0,0001	**-0,0001**	**-0,0002**	0,1708	-0,0001
1	65427476	rs4915675		0,3688	0,3683	-0,0005	0,3678	-0,0010	**-0,0014**	**-0,0027**	0,0939	-0,0016
1	65516055	rs6588109	0,3837	0,4015	0,4012	-0,0002	0,4010	-0,0005	**-0,0006**	**-0,0012**	0,0443	-0,0008
1	65557876	rs6699671	0,2404	0,2271	0,2288	0,0017	0,2305	0,0034	**0,0074**	**0,0147**	-0,0586	0,0044
1	65583858	rs11208591	0,5267	0,4858	0,4885	0,0027	0,4912	0,0054	**0,0055**	**0,0110**	-0,0842	0,0105
1	65619880	rs10789171	0,3106	0,3322	0,3317	-0,0005	0,3313	-0,0009	**-0,0014**	**-0,0028**	0,0650	-0,0014
1	65658412	rs6677316	0,3771	0,3756	0,3757	0,0001	0,3758	0,0002	**0,0003**	**0,0005**	-0,0041	0,0003
3	12286720	rs9850825	0,4605	0,4815	0,4922	0,0106	0,5028	0,0213	**0,0216**	**0,0423**	0,0437	0,0410
3	12302462	rs9878908	0,1925	0,2101	0,2112	0,0011	0,2124	0,0023	**0,0053**	**0,0106**	0,0838	0,0029
3	12393125	rs1801282	0,1416	0,1444	0,1444	0,0001	0,1445	0,0001	**0,0004**	**0,0009**	0,0193	0,0001
3	12402474	rs1373641	0,4135	0,4274	0,4335	0,0061	0,4397	0,0122	**0,0141**	**0,0278**	0,0327	0,0214
3	12475088	rs7626560	0,3194	0,3148	0,3151	0,0003	0,3153	0,0005	**0,0008**	**0,0016**	-0,0145	0,0007
3	151007310	rs9863983	0,2708	0,2890	0,2887	-0,0004	0,2883	-0,0008	**-0,0013**	**-0,0027**	0,0630	-0,0011
3	151041513	rs3971191	0,1950	0,1911	0,1920	0,0009	0,1929	0,0019	**0,0048**	**0,0096**	-0,0205	0,0023
3	151053898	rs7644001	0,4321	0,4648	0,4690	0,0042	0,4732	0,0084	**0,0089**	**0,0177**	0,0705	0,0156
3	151090963	rs9859538	0,4733	0,4913	0,5001	0,0088	0,5089	0,0176	**0,0176**	**0,0346**	0,0367	0,0346
3	151112568	rs3732768	0,2857	0,3047	0,3054	0,0007	0,3060	0,0013	**0,0021**	**0,0043**	0,0625	0,0019
3	151128895	rs10935844	0,5063	0,4985	0,4979	-0,0006	0,4973	-0,0012	**-0,0012**	**-0,0025**	-0,0158	-0,0025
3	151147968	rs6772196	0,1454	0,1577	0,1583	0,0005	0,1588	0,0011	**0,0035**	**0,0069**	0,0785	0,0013
5	51405600	rs12655411	0,4607	0,4469	0,4479	0,0009	0,4488	0,0018	**0,0021**	**0,0041**	-0,0307	0,0033
5	51431680	rs10064799	0,4609	0,4515	0,4645	0,0129	0,4774	0,0258	**0,0278**	**0,0541**	-0,0209	0,0471
5	51505665	rs6865397	0,4953	0,4940	0,4943	0,0004	0,4947	0,0008	**0,0008**	**0,0016**	-0,0027	0,0015
5	51572584	rs4572960	0,4976	0,4874	0,4881	0,0006	0,4887	0,0012	**0,0013**	**0,0025**	-0,0208	0,0024
5	74616843	rs10474433	0,4783	0,4820	0,4818	-0,0002	0,4817	-0,0003	**-0,0003**	**-0,0007**	0,0077	-0,0006
5	74620912	rs6878576	0,2480	0,2559	0,2558	-0,0001	0,2557	-0,0002	**-0,0004**	**-0,0009**	0,0309	-0,0003
5	74625487	rs7703051	0,4273	0,4496	0,4519	0,0023	0,4543	0,0046	**0,0051**	**0,0102**	0,0497	0,0084
5	74648603	rs12654264	0,4238	0,4517	0,4982	0,0465	0,5448	0,0931	**0,0934**	**0,1709**	0,0617	0,1698
5	74651084	rs3846662	0,4614	0,4993	0,4989	-0,0004	0,4985	-0,0009	**-0,0009**	**-0,0017**	0,0760	-0,0017
5	74655726	rs3846663	0,4186	0,4511	0,4531	0,0020	0,4551	0,0040	**0,0045**	**0,0089**	0,0721	0,0074
5	74656175	rs5909	0,1594	0,1613	0,1615	0,0001	0,1616	0,0003	**0,0009**	**0,0018**	0,0119	0,0004
6	160496055	rs3777406	0,2705	0,2744	0,2779	0,0036	0,2815	0,0072	**0,0129**	**0,0255**	0,0142	0,0099
6	160517481	rs1803989	0,1293	0,1256	0,1275	0,0019	0,1295	0,0039	**0,0153**	**0,0301**	-0,0293	0,0045
6	160528057	rs7753051	0,3523	0,3687	0,3682	-0,0005	0,3677	-0,0011	**-0,0015**	**-0,0030**	0,0446	-0,0017
6	160572866	rs622342	0,3791	0,3991	0,4049	0,0058	0,4107	0,0116	**0,0143**	**0,0281**	0,0501	0,0192
6	160578860	rs1564348	0,2617	0,2681	0,2680	-0,0001	0,2679	-0,0002	**-0,0003**	**-0,0007**	0,0237	-0,0003
6	160581374	rs651164	0,4517	0,4582	0,4617	0,0035	0,4653	0,0071	**0,0077**	**0,0153**	0,0142	0,0131
6	160582340	rs9456505	0,1347	0,1387	0,1387	-0,0001	0,1386	-0,0001	**-0,0004**	**-0,0008**	0,0288	-0,0001
6	160635886	rs10945656	0,1845	0,1888	0,1899	0,0012	0,1911	0,0023	**0,0061**	**0,0121**	0,0227	0,0029
6	160637239	rs596881	0,2099	0,2359	0,2386	0,0027	0,2412	0,0053	**0,0112**	**0,0221**	0,1101	0,0070
6	160672625	rs316013	0,3876	0,3738	0,3789	0,0051	0,3840	0,0101	**0,0134**	**0,0264**	-0,0369	0,0162
6	160681393	rs3127573	0,1529	0,1610	0,1611	0,0001	0,1612	0,0002	**0,0006**	**0,0011**	0,0505	0,0002
6	160682897	rs7757997	0,1656	0,1624	0,1664	0,0040	0,1704	0,0079	**0,0238**	**0,0464**	-0,0197	0,0094
6	160687866	rs316030	0,4448	0,4357	0,4407	0,0050	0,4456	0,0100	**0,0113**	**0,0224**	-0,0210	0,0177
6	160699534	rs2619276	0,3709	0,3528	0,3538	0,0010	0,3548	0,0021	**0,0029**	**0,0058**	-0,0514	0,0032
7	87103670	rs2888611	0,3330	0,3377	0,3436	0,0059	0,3495	0,0118	**0,0171**	**0,0337**	0,0138	0,0178
7	87154646	rs10225473	0,2360	0,2356	0,2353	-0,0002	0,2351	-0,0005	**-0,0010**	**-0,0020**	-0,0018	-0,0006
7	87163016	rs11760837	0,2063	0,2217	0,2216	-0,0001	0,2216	-0,0002	**-0,0003**	**-0,0007**	0,0695	-0,0002
7	87179143	rs2235033	0,5112	0,4999	0,5006	0,0007	0,5012	0,0014	**0,0014**	**0,0027**	-0,0226	0,0027
7	87179809	rs2229109	0,0842	0,0963	0,0965	0,0002	0,0968	0,0005	**0,0025**	**0,0050**	0,1253	0,0005
7	87180198	rs10276036	0,5019	0,4870	0,4885	0,0015	0,4899	0,0029	**0,0030**	**0,0059**	-0,0306	0,0057
7	87183354	rs1922240	0,4289	0,4452	0,4448	-0,0004	0,4444	-0,0008	**-0,0009**	**-0,0018**	0,0365	-0,0015
7	87201482	rs10260862	0,3216	0,3302	0,3308	0,0006	0,3314	0,0011	**0,0017**	**0,0034**	0,0261	0,0017
7	87278760	rs10267099	0,3739	0,3630	0,3631	0,0001	0,3631	0,0001	**0,0002**	**0,0003**	-0,0302	0,0002
7	99207876	rs7792939	0,1817	0,1814	0,1835	0,0021	0,1857	0,0043	**0,0117**	**0,0231**	-0,0015	0,0052
10	96581094	rs10786172	0,3850	0,4098	0,4137	0,0039	0,4176	0,0078	**0,0094**	**0,0187**	0,0604	0,0132
10	114711983	rs7094463	0,5230	0,4919	0,4923	0,0004	0,4928	0,0009	**0,0009**	**0,0018**	-0,0633	0,0017
10	114732906	rs7901275	0,4796	0,4873	0,4884	0,0011	0,4895	0,0023	**0,0023**	**0,0046**	0,0158	0,0044
10	114754088	rs7901695	0,4221	0,4739	0,4733	-0,0006	0,4727	-0,0012	**-0,0013**	**-0,0025**	0,1093	-0,0023
10	114756041	rs4506565	0,4146	0,4747	0,4741	-0,0006	0,4735	-0,0013	**-0,0013**	**-0,0027**	0,1266	-0,0024
10	114767771	rs4132670	0,4179	0,4853	0,4855	0,0003	0,4858	0,0006	**0,0006**	**0,0012**	0,1389	0,0011
10	114788815	rs12243326	0,3975	0,4633	0,4627	-0,0006	0,4621	-0,0012	**-0,0013**	**-0,0026**	0,1421	-0,0023
10	114821249	rs11196212	0,4435	0,4911	0,4944	0,0033	0,4978	0,0067	**0,0068**	**0,0134**	0,0970	0,0131
10	114855397	rs11196224	0,4616	0,4711	0,4773	0,0062	0,4835	0,0124	**0,0130**	**0,0256**	0,0202	0,0234

Supplementary Table 2 next

Chr	Localisation	rs ID	Ho	Hs	Ht	Dst	Htp	Dstp	Fst	Fstp	Fis	Dest
10	114859463	rs7085532	0,4115	0,4242	0,4242	0,0000	0,4242	0,0000	**0,0000**	**0,0000**	0,0300	0,0000
10	114898093	rs3814573	0,4383	0,4715	0,4709	-0,0005	0,4704	-0,0011	**-0,0012**	**-0,0023**	0,0704	-0,0021
10	114912534	rs1555485	0,1508	0,1496	0,1525	0,0030	0,1555	0,0059	**0,0195**	**0,0382**	-0,0084	0,0070
11	2528003	rs11023096	0,3006	0,3087	0,3083	-0,0004	0,3079	-0,0008	**-0,0013**	**-0,0026**	0,0262	-0,0011
11	2528233	rs4929992	0,4907	0,4941	0,4974	0,0033	0,5006	0,0065	**0,0066**	**0,0131**	0,0068	0,0129
11	2550730	rs179429	0,2720	0,2943	0,2949	0,0006	0,2956	0,0013	**0,0022**	**0,0043**	0,0756	0,0018
11	2553703	rs179435	0,3520	0,3921	0,3926	0,0005	0,3931	0,0009	**0,0012**	**0,0023**	0,1023	0,0015
11	2595287	rs2283171	0,4171	0,4463	0,4468	0,0005	0,4473	0,0010	**0,0011**	**0,0022**	0,0653	0,0018
11	2617782	rs1116714	0,3580	0,3661	0,3746	0,0084	0,3830	0,0169	**0,0225**	**0,0440**	0,0221	0,0266
11	2633152	rs10766212	0,4689	0,4978	0,4997	0,0020	0,5017	0,0039	**0,0039**	**0,0078**	0,0581	0,0078
11	2635797	rs2106467	0,4642	0,4842	0,4841	-0,0001	0,4840	-0,0002	**-0,0002**	**-0,0003**	0,0413	-0,0003
11	2673575	rs6578283	0,4726	0,4702	0,4698	-0,0003	0,4695	-0,0007	**-0,0007**	**-0,0014**	-0,0051	-0,0013
11	2750703	rs170786	0,4480	0,4442	0,4471	0,0029	0,4500	0,0057	**0,0064**	**0,0127**	-0,0084	0,0103
11	2776448	rs11023996	0,1408	0,1512	0,1514	0,0002	0,1516	0,0004	**0,0013**	**0,0027**	0,0690	0,0005
11	2782648	rs548566	0,2559	0,2802	0,2798	-0,0004	0,2794	-0,0008	**-0,0014**	**-0,0028**	0,0869	-0,0011
11	2821065	rs163171	0,3242	0,3455	0,3455	0,0001	0,3456	0,0001	**0,0002**	**0,0004**	0,0617	0,0002
11	2837625	rs233446	0,3390	0,3518	0,3546	0,0027	0,3573	0,0055	**0,0077**	**0,0153**	0,0366	0,0084
11	2850782	rs234852	0,4065	0,4384	0,4438	0,0054	0,4492	0,0108	**0,0122**	**0,0241**	0,0727	0,0193
11	2895800	rs3987740	0,4282	0,4508	0,4504	-0,0004	0,4500	-0,0007	**-0,0008**	**-0,0017**	0,0500	-0,0014
11	17393644	rs12791318	0,2830	0,3078	0,3097	0,0019	0,3116	0,0037	**0,0060**	**0,0120**	0,0808	0,0054
11	17405333	rs10832785	0,4328	0,4819	0,4877	0,0058	0,4935	0,0116	**0,0119**	**0,0236**	0,1017	0,0225
11	17408025	rs2285676	0,4448	0,4828	0,4874	0,0046	0,4921	0,0093	**0,0095**	**0,0189**	0,0787	0,0180
11	17408630	rs5215	0,2849	0,3444	0,3598	0,0153	0,3751	0,0306	**0,0426**	**0,0817**	0,1729	0,0467
11	17408831	rs1800467	0,0791	0,0822	0,0830	0,0008	0,0838	0,0016	**0,0096**	**0,0189**	0,0370	0,0017
11	17438890	rs2074315	0,3103	0,3280	0,3279	-0,0001	0,3278	-0,0002	**-0,0003**	**-0,0006**	0,0540	-0,0003
11	17441828	rs4757517	0,3933	0,3977	0,3972	-0,0005	0,3967	-0,0010	**-0,0013**	**-0,0025**	0,0112	-0,0017
11	17496516	rs1048099	0,4833	0,4791	0,4785	-0,0006	0,4779	-0,0012	**-0,0012**	**-0,0024**	-0,0089	-0,0022
11	17510419	rs11603988	0,2096	0,2027	0,2053	0,0026	0,2079	0,0052	**0,0127**	**0,0252**	-0,0342	0,0066
11	17510565	rs4757527	0,3588	0,3866	0,3870	0,0004	0,3874	0,0008	**0,0010**	**0,0019**	0,0719	0,0012
11	17530484	rs7104083	0,4838	0,4925	0,4934	0,0008	0,4942	0,0016	**0,0016**	**0,0033**	0,0178	0,0032
11	17532597	rs1076311	0,4915	0,4911	0,4928	0,0017	0,4945	0,0034	**0,0034**	**0,0068**	-0,0008	0,0066
11	17542649	rs2041032	0,4599	0,4989	0,4983	-0,0006	0,4977	-0,0011	**-0,0012**	**-0,0023**	0,0781	-0,0023
11	108097333	rs228591	0,4275	0,4506	0,4510	0,0004	0,4514	0,0008	**0,0009**	**0,0017**	0,0513	0,0014
11	108268286	rs7931930	0,4540	0,4672	0,4759	0,0087	0,4846	0,0173	**0,0182**	**0,0358**	0,0284	0,0325
11	108283161	rs11212617	0,4252	0,4544	0,4556	0,0012	0,4568	0,0023	**0,0026**	**0,0051**	0,0644	0,0043
16	31102321	rs7294	0,3654	0,3921	0,3974	0,0053	0,4028	0,0107	**0,0134**	**0,0265**	0,0681	0,0175
17	19447016	rs2440155	0,2759	0,2734	0,2761	0,0026	0,2787	0,0053	**0,0096**	**0,0190**	-0,0090	0,0073
17	19459537	rs2244280	0,2715	0,2995	0,3008	0,0012	0,3020	0,0024	**0,0040**	**0,0080**	0,0936	0,0034
17	19484951	rs2453594	0,2793	0,3096	0,3098	0,0001	0,3099	0,0003	**0,0004**	**0,0009**	0,0979	0,0004
17	19622643	rs11656096	0,2695	0,2942	0,3061	0,0119	0,3180	0,0238	**0,0389**	**0,0749**	0,0839	0,0337
17	19642952	rs2228100	0,4088	0,4338	0,4986	0,0648	0,5635	0,1297	**0,1300**	**0,2302**	0,0576	0,2290
17	19645938	rs887241	0,4531	0,4792	0,4863	0,0071	0,4934	0,0142	**0,0146**	**0,0287**	0,0545	0,0272
22	42152988	rs17377643	0,4474	0,4568	0,4608	0,0040	0,4648	0,0080	**0,0087**	**0,0172**	0,0205	0,0147
22	42178441	rs126092	0,3208	0,3866	0,3894	0,0028	0,3922	0,0056	**0,0072**	**0,0143**	0,1701	0,0092
22	46235677	rs1023470	0,3167	0,3395	0,3421	0,0026	0,3447	0,0051	**0,0075**	**0,0149**	0,0674	0,0078
22	46238069	rs8141212	0,4951	0,4675	0,4674	0,0000	0,4674	-0,0001	**-0,0001**	**-0,0002**	-0,0591	-0,0002
22	46525794	rs6007919	0,4060	0,4458	0,4452	-0,0006	0,4446	-0,0012	**-0,0013**	**-0,0026**	0,0892	-0,0021
22	46629479	rs4253776	0,2054	0,2279	0,2300	0,0020	0,2320	0,0041	**0,0089**	**0,0176**	0,0989	0,0053
22	46637254	rs9626814	0,1746	0,2125	0,2146	0,0021	0,2168	0,0043	**0,0100**	**0,0198**	0,1782	0,0055
22	46643774	rs16995069	0,1484	0,1546	0,1544	-0,0002	0,1542	-0,0004	**-0,0012**	**-0,0025**	0,0402	-0,0005
22	46670394	rs6007761	0,2101	0,2563	0,2560	-0,0003	0,2557	-0,0006	**-0,0012**	**-0,0023**	0,1802	-0,0008

$overall	Ho	Hs	Ht	Dst	Htp	Dstp	Fst	Fstp	Fis	Dest
	0,3480	0,3636	0,3666	0,0029	0,3695	0,0058	**0,0079**	**0,0158**	0,0431	0,0092

$FST 0,0158

$FIS 0,0205

Supplementary Table 2 next

Fst Comparison among Tunisian and South Italian populations

Chr	Localisation	rs ID	Ho	Hs	Ht	Dst	Htp	Dstp	Fst	Fstp	Fis	Dest
1	65381861	rs12563017	0,1815	0,2182	0,2191	0,0009	0,2200	0,0017	**0,0040**	**0,0079**	0,1684	0,0022
1	65389835	rs10889503	0,3096		0,3594	0,0046	0,3640	0,0092	**0,0128**	**0,0253**	0,1272	0,0143
1	65421058	rs4916014	0,3395	0,4233	0,4226	-0,0007	0,4220	-0,0013	**-0,0016**	**-0,0031**	0,1979	-0,0023
1	65427476	rs4915675		0,3726	0,3719	-0,0007	0,3713	-0,0013	**-0,0018**	**-0,0036**	0,0733	-0,0021
1	65516055	rs6588109	0,3871	0,4108	0,4101	-0,0007	0,4093	-0,0015	**-0,0018**	**-0,0036**	0,0577	-0,0025
1	65557876	rs6699671	0,2395	0,2317	0,2330	0,0012	0,2342	0,0025	**0,0053**	**0,0106**	-0,0336	0,0032
1	65583858	rs11208591	0,5037	0,4891	0,4906	0,0015	0,4921	0,0030	**0,0031**	**0,0062**	-0,0299	0,0060
1	65619880	rs10789171	0,3263	0,3341	0,3335	-0,0006	0,3329	-0,0012	**-0,0018**	**-0,0037**	0,0233	-0,0018
1	65658412	rs6677316	0,3908	0,3710	0,3713	0,0003	0,3716	0,0006	**0,0008**	**0,0016**	-0,0532	0,0010
3	12286720	rs9850825	0,4991	0,4813	0,4871	0,0057	0,4928	0,0115	**0,0118**	**0,0233**	-0,0370	0,0222
3	12302462	rs9878908	0,2002	0,2018	0,2023	0,0005	0,2028	0,0010	**0,0025**	**0,0049**	0,0081	0,0013
3	12393125	rs1801282	0,1270	0,1314	0,1312	-0,0002	0,1310	-0,0004	**-0,0016**	**-0,0033**	0,0335	-0,0005
3	12402474	rs1373641	0,4192	0,4295	0,4362	0,0067	0,4429	0,0134	**0,0154**	**0,0303**	0,0241	0,0235
3	12475088	rs7626560	0,2924	0,2795	0,2792	-0,0002	0,2790	-0,0005	**-0,0009**	**-0,0018**	-0,0462	-0,0007
3	151007310	rs9863983	0,3032	0,3092	0,3092	0,0001	0,3093	0,0001	**0,0002**	**0,0004**	0,0193	0,0002
3	151041513	rs3971191	0,1859	0,1741	0,1741	0,0000	0,1741	0,0001	**0,0003**	**0,0005**	-0,0680	0,0001
3	151053898	rs7644001	0,4445	0,4681	0,4713	0,0032	0,4745	0,0063	**0,0067**	**0,0133**	0,0505	0,0119
3	151090963	rs9859538	0,5028	0,4910	0,5002	0,0092	0,5093	0,0184	**0,0184**	**0,0361**	-0,0241	0,0361
3	151112568	rs3732768	0,2660	0,2983	0,2993	0,0010	0,3003	0,0020	**0,0033**	**0,0067**	0,1082	0,0029
3	151128895	rs10935844	0,4865	0,4944	0,4943	-0,0001	0,4941	-0,0003	**-0,0003**	**-0,0005**	0,0160	-0,0005
3	151147968	rs6772196	0,1250	0,1469	0,1480	0,0010	0,1490	0,0021	**0,0070**	**0,0139**	0,1492	0,0024
5	51405600	rs12655411	0,4207	0,4358	0,4351	-0,0007	0,4344	-0,0013	**-0,0015**	**-0,0030**	0,0344	-0,0023
5	51431680	rs10064799	0,4323	0,4421	0,4468	0,0046	0,4514	0,0093	**0,0104**	**0,0205**	0,0223	0,0166
5	51505665	rs6865397	0,4791	0,4830	0,4826	-0,0003	0,4823	-0,0006	**-0,0006**	**-0,0013**	0,0080	-0,0012
5	51572584	rs4572960	0,4779	0,4776	0,4768	-0,0008	0,4761	-0,0015	**-0,0016**	**-0,0032**	-0,0007	-0,0029
5	74616843	rs10474433	0,4845	0,4909	0,4902	-0,0007	0,4895	-0,0014	**-0,0014**	**-0,0029**	0,0130	-0,0028
5	74620912	rs6878576	0,2604	0,2879	0,2899	0,0020	0,2919	0,0041	**0,0070**	**0,0139**	0,0954	0,0057
5	74625487	rs7703051	0,4323	0,4548	0,4587	0,0040	0,4627	0,0079	**0,0086**	**0,0171**	0,0495	0,0145
5	74648603	rs12654264	0,4300	0,4571	0,4966	0,0395	0,5361	0,0790	**0,0796**	**0,1474**	0,0593	0,1456
5	74651084	rs3846662	0,4863	0,5006	0,4998	-0,0008	0,4989	-0,0017	**-0,0017**	**-0,0034**	0,0286	-0,0034
5	74655726	rs3846663	0,4179	0,4556	0,4589	0,0033	0,4622	0,0066	**0,0071**	**0,0142**	0,0829	0,0120
5	74656175	rs5909	0,1435	0,1622	0,1623	0,0001	0,1623	0,0001	**0,0004**	**0,0007**	0,1154	0,0001
6	160496055	rs3777406	0,2379	0,2740	0,2773	0,0033	0,2806	0,0066	**0,0118**	**0,0234**	0,1317	0,0091
6	160517481	rs1803989	0,1414	0,1368	0,1380	0,0012	0,1391	0,0023	**0,0084**	**0,0166**	-0,0332	0,0027
6	160528057	rs7753051	0,3244	0,3660	0,3653	-0,0007	0,3646	-0,0014	**-0,0020**	**-0,0040**	0,1137	-0,0023
6	160572866	rs622342	0,3996	0,3999	0,4056	0,0057	0,4114	0,0115	**0,0141**	**0,0279**	0,0007	0,0191
6	160578860	rs1564348	0,2735	0,2682	0,2680	-0,0002	0,2678	-0,0004	**-0,0008**	**-0,0015**	-0,0199	-0,0006
6	160581374	rs651164	0,4736	0,4671	0,4685	0,0014	0,4699	0,0028	**0,0030**	**0,0059**	-0,0139	0,0052
6	160582340	rs9456505	0,1297	0,1278	0,1280	0,0002	0,1282	0,0004	**0,0014**	**0,0028**	-0,0145	0,0004
6	160635886	rs10945656	0,1868	0,1921	0,1929	0,0009	0,1938	0,0017	**0,0045**	**0,0090**	0,0276	0,0021
6	160637239	rs596881	0,1795	0,2064	0,2124	0,0060	0,2183	0,0119	**0,0280**	**0,0545**	0,1305	0,0150
6	160672625	rs316013	0,3446	0,3437	0,3547	0,0110	0,3657	0,0220	**0,0309**	**0,0600**	-0,0026	0,0335
6	160681393	rs3127573	0,1552	0,1730	0,1727	-0,0003	0,1725	-0,0006	**-0,0016**	**-0,0033**	0,1031	-0,0007
6	160682897	rs7757997	0,1447	0,1407	0,1470	0,0063	0,1533	0,0127	**0,0431**	**0,0826**	-0,0284	0,0147
6	160687866	rs316030	0,4548	0,4315	0,4346	0,0031	0,4378	0,0063	**0,0072**	**0,0143**	-0,0541	0,0110
6	160699534	rs2619276	0,3557	0,3383	0,3380	-0,0002	0,3378	-0,0005	**-0,0007**	**-0,0014**	-0,0514	-0,0007
7	87103670	rs2888611	0,3457	0,3421	0,3471	0,0051	0,3522	0,0101	**0,0146**	**0,0287**	-0,0107	0,0154
7	87154646	rs10225473	0,2271	0,2483	0,2478	-0,0005	0,2474	-0,0009	**-0,0019**	**-0,0038**	0,0855	-0,0012
7	87163016	rs11760837	0,2063	0,2334	0,2337	0,0002	0,2339	0,0005	**0,0011**	**0,0021**	0,1163	0,0007
7	87179143	rs2235033	0,4917	0,5002	0,4994	-0,0009	0,4985	-0,0017	**-0,0017**	**-0,0035**	0,0170	-0,0035
7	87179809	rs2229109	0,0617	0,0729	0,0728	-0,0001	0,0726	-0,0003	**-0,0020**	**-0,0040**	0,1543	-0,0003
7	87180198	rs10276036	0,4974	0,4837	0,4834	-0,0003	0,4831	-0,0006	**-0,0006**	**-0,0012**	-0,0284	-0,0011
7	87183354	rs1922240	0,4130	0,4511	0,4502	-0,0008	0,4494	-0,0017	**-0,0019**	**-0,0038**	0,0844	-0,0031
7	87201482	rs10260862	0,3352	0,3364	0,3364	0,0000	0,3364	0,0000	**0,0001**	**0,0001**	0,0036	0,0001
7	87278760	rs10267099	0,3874	0,3646	0,3644	-0,0002	0,3642	-0,0003	**-0,0004**	**-0,0009**	-0,0626	-0,0005
7	99207876	rs7792939	0,1680	0,1895	0,1923	0,0028	0,1952	0,0056	**0,0146**	**0,0288**	0,1135	0,0069
10	96581094	rs10786172	0,3787	0,3927	0,3931	0,0004	0,3935	0,0008	**0,0010**	**0,0021**	0,0356	0,0014
10	114711983	rs7094463	0,4846	0,4920	0,4922	0,0003	0,4925	0,0006	**0,0006**	**0,0011**	0,0150	0,0011
10	114732906	rs7901275	0,4484	0,4821	0,4814	-0,0008	0,4806	-0,0015	**-0,0016**	**-0,0032**	0,0701	-0,0029
10	114754088	rs7901695	0,4276	0,4678	0,4671	-0,0008	0,4663	-0,0015	**-0,0016**	**-0,0032**	0,0861	-0,0028
10	114756041	rs4506565	0,4246	0,4693	0,4685	-0,0008	0,4677	-0,0016	**-0,0017**	**-0,0034**	0,0952	-0,0030
10	114767771	rs4132670	0,4248	0,4787	0,4804	0,0017	0,4821	0,0034	**0,0036**	**0,0072**	0,1125	0,0066
10	114788815	rs12243326	0,3935	0,4536	0,4530	-0,0006	0,4524	-0,0012	**-0,0013**	**-0,0027**	0,1324	-0,0022
10	114821249	rs11196212	0,4114	0,4917	0,4945	0,0029	0,4974	0,0057	**0,0058**	**0,0116**	0,1632	0,0113
10	114855397	rs11196224	0,4955	0,4859	0,4872	0,0013	0,4885	0,0027	**0,0027**	**0,0054**	-0,0198	0,0052

Supplementary Table 2 next

Fst_TUN_ITAS 1

Chr	Localisation	rs ID	Ho	Hs	Ht	Dst	Htp	Dstp	Fst	Fstp	Fis	Dest
10	114859463	rs7085532	0,3945	0,4061	0,4055	-0,0007	0,4048	-0,0013	**-0,0016**	**-0,0032**	0,0286	-0,0022
10	114898093	rs3814573	0,4374	0,4703	0,4696	-0,0007	0,4690	-0,0013	**-0,0014**	**-0,0028**	0,0699	-0,0025
10	114912534	rs1555485	0,1348	0,1609	0,1651	0,0041	0,1692	0,0083	**0,0250**	**0,0488**	0,1624	0,0098
11	2528003	rs11023096	0,2695	0,3004	0,3000	-0,0005	0,2995	-0,0010	**-0,0016**	**-0,0032**	0,1031	-0,0014
11	2528233	rs4929992	0,4566	0,4917	0,4912	-0,0005	0,4907	-0,0010	**-0,0010**	**-0,0020**	0,0714	-0,0019
11	2550730	rs179429	0,2903	0,3091	0,3110	0,0019	0,3130	0,0039	**0,0062**	**0,0124**	0,0606	0,0056
11	2553703	rs179435	0,3484	0,4096	0,4088	-0,0008	0,4080	-0,0016	**-0,0020**	**-0,0040**	0,1495	-0,0027
11	2595287	rs2283171	0,3982	0,4461	0,4465	0,0003	0,4468	0,0006	**0,0007**	**0,0014**	0,1074	0,0011
11	2617782	rs1116714	0,3493	0,3647	0,3733	0,0086	0,3819	0,0173	**0,0231**	**0,0452**	0,0420	0,0272
11	2633152	rs10766212	0,4432	0,5005	0,5008	0,0003	0,5011	0,0006	**0,0006**	**0,0012**	0,1143	0,0012
11	2635797	rs2106467	0,4466	0,4870	0,4873	0,0003	0,4876	0,0006	**0,0007**	**0,0013**	0,0829	0,0013
11	2673575	rs6578283	0,4772	0,4778	0,4770	-0,0008	0,4761	-0,0016	**-0,0017**	**-0,0034**	0,0011	-0,0031
11	2750703	rs170786	0,4646	0,4532	0,4543	0,0011	0,4553	0,0021	**0,0023**	**0,0047**	-0,0251	0,0039
11	2776448	rs11023996	0,1177	0,1377	0,1374	-0,0002	0,1372	-0,0004	**-0,0015**	**-0,0031**	0,1448	-0,0005
11	2782648	rs548566	0,2088	0,2318	0,2334	0,0016	0,2350	0,0032	**0,0068**	**0,0136**	0,0994	0,0042
11	2821065	rs163171	0,3158	0,3551	0,3545	-0,0006	0,3539	-0,0011	**-0,0016**	**-0,0032**	0,1106	-0,0017
11	2837625	rs233446	0,3576	0,3612	0,3656	0,0044	0,3700	0,0088	**0,0120**	**0,0237**	0,0099	0,0138
11	2850782	rs234852	0,3561	0,4055	0,4200	0,0145	0,4344	0,0290	**0,0345**	**0,0667**	0,1219	0,0487
11	2895800	rs3987740	0,4259	0,4362	0,4372	0,0010	0,4383	0,0021	**0,0024**	**0,0047**	0,0237	0,0037
11	17393644	rs12791318	0,3045	0,3146	0,3172	0,0026	0,3198	0,0052	**0,0082**	**0,0163**	0,0322	0,0076
11	17405333	rs10832785	0,4424	0,4735	0,4828	0,0093	0,4921	0,0187	**0,0193**	**0,0379**	0,0657	0,0354
11	17408025	rs2285676	0,4503	0,4709	0,4803	0,0094	0,4897	0,0188	**0,0196**	**0,0384**	0,0436	0,0355
11	17408630	rs5215	0,2843	0,3459	0,3616	0,0157	0,3773	0,0314	**0,0434**	**0,0831**	0,1782	0,0479
11	17408831	rs1800467	0,0764	0,0846	0,0854	0,0009	0,0863	0,0017	**0,0100**	**0,0198**	0,0965	0,0019
11	17438890	rs2074315	0,3077	0,3256	0,3252	-0,0004	0,3248	-0,0007	**-0,0012**	**-0,0023**	0,0549	-0,0011
11	17441828	rs4757517	0,3986	0,4035	0,4029	-0,0006	0,4022	-0,0013	**-0,0016**	**-0,0031**	0,0121	-0,0021
11	17496516	rs1048099	0,5057	0,4858	0,4859	0,0002	0,4861	0,0003	**0,0003**	**0,0007**	-0,0410	0,0006
11	17510419	rs11603988	0,1550	0,1647	0,1647	0,0000	0,1647	0,0000	**-0,0001**	**-0,0001**	0,0585	0,0000
11	17510565	rs4757527	0,3331	0,3881	0,3884	0,0002	0,3886	0,0005	**0,0006**	**0,0012**	0,1419	0,0007
11	17530484	rs7104083	0,4359	0,4906	0,4900	-0,0006	0,4894	-0,0012	**-0,0012**	**-0,0025**	0,1115	-0,0024
11	17532597	rs1076311	0,4291	0,4885	0,4881	-0,0004	0,4877	-0,0009	**-0,0009**	**-0,0018**	0,1217	-0,0017
11	17542649	rs2041032	0,4766	0,4999	0,4993	-0,0006	0,4987	-0,0012	**-0,0012**	**-0,0024**	0,0466	-0,0024
11	108097333	rs228591	0,4433	0,4483	0,4488	0,0005	0,4494	0,0011	**0,0012**	**0,0024**	0,0110	0,0020
11	108268286	rs7931930	0,4657	0,4642	0,4740	0,0098	0,4838	0,0195	**0,0206**	**0,0404**	-0,0030	0,0365
11	108283161	rs11212617	0,4369	0,4515	0,4530	0,0016	0,4546	0,0031	**0,0034**	**0,0068**	0,0323	0,0057
16	31102321	rs7294	0,3123	0,3804	0,3825	0,0021	0,3846	0,0042	**0,0055**	**0,0109**	0,1791	0,0068
17	19447016	rs2440155	0,2490	0,2602	0,2643	0,0040	0,2683	0,0080	**0,0152**	**0,0300**	0,0432	0,0109
17	19459537	rs2244280	0,2421	0,2857	0,2881	0,0024	0,2905	0,0048	**0,0083**	**0,0164**	0,1526	0,0067
17	19484951	rs2453594	0,2554	0,2804	0,2828	0,0024	0,2853	0,0048	**0,0085**	**0,0169**	0,0892	0,0067
17	19622643	rs11656096	0,2506	0,2746	0,2810	0,0064	0,2874	0,0128	**0,0228**	**0,0446**	0,0875	0,0177
17	19642952	rs2228100	0,3948	0,4460	0,5003	0,0543	0,5547	0,1086	**0,1086**	**0,1959**	0,1148	0,1961
17	19645938	rs887241	0,4788	0,4967	0,4968	0,0001	0,4969	0,0002	**0,0002**	**0,0005**	0,0360	0,0005
22	42152988	rs17377643	0,4488	0,4486	0,4495	0,0009	0,4504	0,0018	**0,0020**	**0,0039**	-0,0004	0,0032
22	42178441	rs126092	0,3492	0,4250	0,4240	-0,0010	0,4229	-0,0021	**-0,0024**	**-0,0049**	0,1784	-0,0036
22	46235677	rs1023470	0,3049	0,3346	0,3362	0,0016	0,3378	0,0032	**0,0048**	**0,0095**	0,0888	0,0048
22	46238069	rs8141212	0,5095	0,4784	0,4822	0,0039	0,4861	0,0077	**0,0080**	**0,0159**	-0,0650	0,0148
22	46525794	rs6007919	0,4195	0,4550	0,4542	-0,0007	0,4535	-0,0015	**-0,0016**	**-0,0033**	0,0780	-0,0027
22	46629479	rs4253776	0,2366	0,2610	0,2608	-0,0002	0,2607	-0,0003	**-0,0006**	**-0,0013**	0,0935	-0,0004
22	46637254	rs9626814	0,2081	0,2501	0,2498	-0,0003	0,2495	-0,0005	**-0,0011**	**-0,0022**	0,1678	-0,0007
22	46643774	rs16995069	0,1003	0,1074	0,1089	0,0015	0,1103	0,0029	**0,0134**	**0,0265**	0,0664	0,0033
22	46670394	rs6007761	0,2244	0,2599	0,2594	-0,0005	0,2589	-0,0010	**-0,0019**	**-0,0038**	0,1368	-0,0013

$overall	Ho	Hs	Ht	Dst	Htp	Dstp	Fst	Fstp	Fis	Dest
	0,3430	0,3621	0,3646	0,0025	0,3671	0,0050	**0,0068**	**0,0135**	0,0529	0,0078

$FST 0,0135

$FIS 0,0492

Supplementary Table 2 next

Fst Comparison among Tunisian and Central Toscan populations

Chr	Localisation	rs ID	Ho	Hs	Ht	Dst	Htp	Dstp	Fst	Fstp	Fis	Dest
1	65381861	rs12563017	0,1231	0,1712	0,1708	-0,0004	0,1704	-0,0008	**-0,0025**	**-0,0049**	0,2811	-0,0010
1	65389835	rs10889503	0,2449		0,3109	0,0169	0,3278	0,0337	**0,0543**	**0,1029**	0,1673	0,0478
1	65421058	rs4916014	0,2623	0,3817	0,3865	0,0048	0,3914	0,0097	**0,0125**	**0,0248**	0,3129	0,0157
1	65427476	rs4915675		0,3315	0,3327	0,0013	0,3340	0,0025	**0,0038**	**0,0075**	0,2283	0,0038
1	65516055	rs6588109	0,3020	0,3839	0,3847	0,0008	0,3856	0,0016	**0,0021**	**0,0043**	0,2134	0,0027
1	65557876	rs6699671	0,2102	0,2259	0,2275	0,0016	0,2290	0,0032	**0,0070**	**0,0138**	0,0692	0,0041
1	65583858	rs11208591	0,4350	0,4640	0,4748	0,0109	0,4857	0,0217	**0,0229**	**0,0447**	0,0624	0,0405
1	65619880	rs10789171	0,2917	0,3338	0,3330	-0,0008	0,3321	-0,0016	**-0,0024**	**-0,0049**	0,1261	-0,0024
1	65658412	rs6677316	0,3480	0,3551	0,3568	0,0017	0,3586	0,0035	**0,0049**	**0,0097**	0,0199	0,0054
3	12286720	rs9850825	0,4291	0,4789	0,4809	0,0020	0,4830	0,0041	**0,0042**	**0,0084**	0,1040	0,0078
3	12302462	rs9878908	0,1718	0,1850	0,1847	-0,0002	0,1845	-0,0005	**-0,0013**	**-0,0026**	0,0712	-0,0006
3	12393125	rs1801282	0,1438	0,1403	0,1401	-0,0001	0,1400	-0,0003	**-0,0010**	**-0,0021**	-0,0253	-0,0003
3	12402474	rs1373641	0,4048	0,4155	0,4173	0,0017	0,4190	0,0035	**0,0042**	**0,0083**	0,0258	0,0059
3	12475088	rs7626560	0,3444	0,3214	0,3219	0,0005	0,3223	0,0009	**0,0014**	**0,0028**	-0,0716	0,0013
3	151007310	rs9863983	0,2578	0,2844	0,2837	-0,0007	0,2831	-0,0013	**-0,0023**	**-0,0046**	0,0936	-0,0018
3	151041513	rs3971191	0,1768	0,1839	0,1843	0,0003	0,1846	0,0006	**0,0018**	**0,0035**	0,0388	0,0008
3	151053898	rs7644001	0,4567	0,4663	0,4699	0,0036	0,4735	0,0072	**0,0077**	**0,0152**	0,0206	0,0135
3	151090963	rs9859538	0,4503	0,4940	0,5009	0,0069	0,5078	0,0137	**0,0137**	**0,0271**	0,0886	0,0272
3	151112568	rs3732768	0,2891	0,3291	0,3283	-0,0008	0,3275	-0,0016	**-0,0025**	**-0,0050**	0,1216	-0,0024
3	151128895	rs10935844	0,4987	0,5007	0,5005	-0,0001	0,5004	-0,0003	**-0,0003**	**-0,0006**	0,0039	-0,0006
3	151147968	rs6772196	0,1347	0,1442	0,1454	0,0012	0,1466	0,0023	**0,0080**	**0,0159**	0,0658	0,0027
5	51405600	rs12655411	0,5152	0,4542	0,4568	0,0025	0,4593	0,0050	**0,0055**	**0,0110**	-0,1342	0,0092
5	51431680	rs10064799	0,4664	0,4532	0,4675	0,0143	0,4818	0,0285	**0,0305**	**0,0593**	-0,0291	0,0522
5	51505665	rs6865397	0,4893	0,4960	0,4980	0,0020	0,5000	0,0040	**0,0041**	**0,0081**	0,0135	0,0080
5	51572584	rs4572960	0,4815	0,4803	0,4803	0,0000	NA	NA	**0,0000**	**NaN**	-0,0025	NA
5	74616843	rs10474433	0,4874	0,4857	0,4848	-0,0009	0,4839	-0,0018	**-0,0018**	**-0,0037**	-0,0035	-0,0035
5	74620912	rs6878576	0,2701	0,2701	0,2705	0,0003	0,2708	0,0007	**0,0012**	**0,0024**	0,0001	0,0009
5	74625487	rs7703051	0,4305	0,4496	0,4512	0,0016	0,4529	0,0033	**0,0036**	**0,0073**	0,0425	0,0060
5	74648603	rs12654264	0,4282	0,4519	0,4531	0,0012	0,4543	0,0024	**0,0027**	**0,0053**	0,0524	0,0044
5	74651084	rs3846662	0,4854	0,4969	0,4971	0,0002	0,4974	0,0005	**0,0005**	**0,0010**	0,0231	0,0010
5	74655726	rs3846663	0,4146	0,4522	0,4539	0,0017	0,4555	0,0033	**0,0037**	**0,0073**	0,0831	0,0061
5	74656175	rs5909	0,1209	0,1434	0,1430	-0,0003	0,1427	-0,0007	**-0,0024**	**-0,0048**	0,1567	-0,0008
6	160496055	rs3777406	0,2441	0,2451	0,2454	0,0003	0,2457	0,0007	**0,0014**	**0,0027**	0,0040	0,0009
6	160517481	rs1803989	0,1494	0,1441	0,1448	0,0007	0,1455	0,0014	**0,0049**	**0,0097**	-0,0364	0,0016
6	160528057	rs7753051	0,3228	0,3610	0,3603	-0,0007	0,3596	-0,0014	**-0,0019**	**-0,0039**	0,1060	-0,0022
6	160572866	rs622342	0,3697	0,3996	0,4048	0,0053	0,4101	0,0106	**0,0130**	**0,0257**	0,0746	0,0176
6	160578860	rs1564348	0,2510	0,2699	0,2695	-0,0004	0,2691	-0,0008	**-0,0015**	**-0,0030**	0,0700	-0,0011
6	160581374	rs651164	0,4940	0,4707	0,4713	0,0006	0,4719	0,0012	**0,0013**	**0,0025**	-0,0494	0,0023
6	160582340	rs9456505	0,1500	0,1456	0,1454	-0,0003	0,1451	-0,0005	**-0,0018**	**-0,0037**	-0,0296	-0,0006
6	160635886	rs10945656	0,2080	0,2043	0,2045	0,0002	0,2047	0,0003	**0,0008**	**0,0016**	-0,0178	0,0004
6	160637239	rs596881	0,1948	0,2353	0,2378	0,0025	0,2403	0,0050	**0,0106**	**0,0209**	0,1720	0,0066
6	160672625	rs316013	0,3706	0,3854	0,3883	0,0029	0,3912	0,0058	**0,0074**	**0,0148**	0,0384	0,0094
6	160681393	rs3127573	0,1828	0,1874	0,1870	-0,0004	0,1866	-0,0008	**-0,0021**	**-0,0042**	0,0245	-0,0010
6	160682897	rs7757997	0,1596	0,1549	0,1595	0,0047	0,1642	0,0093	**0,0292**	**0,0567**	-0,0306	0,0110
6	160687866	rs316030	0,4726	0,4412	0,4484	0,0073	0,4557	0,0145	**0,0162**	**0,0319**	-0,0713	0,0260
6	160699534	rs2619276	0,3897	0,3490	0,3494	0,0004	0,3498	0,0007	**0,0011**	**0,0021**	-0,1165	0,0012
7	87103670	rs2888611	0,3510	0,3592	0,3616	0,0024	0,3640	0,0048	**0,0067**	**0,0133**	0,0227	0,0075
7	87154646	rs10225473	0,2676	0,2563	0,2560	-0,0004	0,2556	-0,0008	**-0,0015**	**-0,0030**	-0,0441	-0,0010
7	87163016	rs11760837	0,2498	0,2433	0,2441	0,0009	0,2450	0,0017	**0,0035**	**0,0070**	-0,0267	0,0023
7	87179143	rs2235033	0,4865	0,5012	0,5003	-0,0009	0,4994	-0,0017	**-0,0017**	**-0,0035**	0,0294	-0,0035
7	87179809	rs2229109	0,0857	0,0973	0,0974	0,0002	0,0976	0,0003	**0,0018**	**0,0036**	0,1188	0,0004
7	87180198	rs10276036	0,4781	0,4837	0,4831	-0,0006	0,4825	-0,0011	**-0,0012**	**-0,0024**	0,0116	-0,0022
7	87183354	rs1922240	0,4059	0,4421	0,4416	-0,0006	0,4410	-0,0012	**-0,0013**	**-0,0027**	0,0820	-0,0021
7	87201482	rs10260862	0,3429	0,3482	0,3475	-0,0006	0,3469	-0,0013	**-0,0018**	**-0,0037**	0,0152	-0,0020
7	87278760	rs10267099	0,3978	0,3748	0,3741	-0,0007	0,3734	-0,0014	**-0,0018**	**-0,0036**	-0,0613	-0,0022
7	99207876	rs7792939	0,1461	0,1484	0,1485	0,0000	0,1485	0,0000	**0,0001**	**0,0002**	0,0159	0,0000
10	96581094	rs10786172	0,3713	0,4083	0,4111	0,0029	0,4140	0,0058	**0,0070**	**0,0139**	0,0905	0,0097
10	114711983	rs7094463	0,5155	0,4843	0,4869	0,0026	0,4895	0,0052	**0,0054**	**0,0107**	-0,0644	0,0101
10	114732906	rs7901275	0,5044	0,4899	0,4924	0,0026	0,4950	0,0051	**0,0052**	**0,0103**	-0,0296	0,0100
10	114754088	rs7901695	0,4103	0,4702	0,4691	-0,0011	0,4680	-0,0021	**-0,0023**	**-0,0046**	0,1274	-0,0040
10	114756041	rs4506565	0,3994	0,4720	0,4708	-0,0011	0,4697	-0,0023	**-0,0024**	**-0,0049**	0,1538	-0,0043
10	114767771	rs4132670	0,3918	0,4785	0,4802	0,0016	0,4818	0,0033	**0,0034**	**0,0068**	0,1813	0,0063
10	114788815	rs12243326	0,3467	0,4415	0,4420	0,0006	0,4426	0,0011	**0,0013**	**0,0025**	0,2146	0,0020
10	114821249	rs11196212	0,4417	0,4913	0,4928	0,0015	0,4944	0,0030	**0,0031**	**0,0061**	0,1011	0,0059
10	114855397	rs11196224	0,4669	0,4728	0,4783	0,0055	0,4838	0,0109	**0,0114**	**0,0226**	0,0126	0,0208

Supplementary Table 2 next

Chr	Localisation	rs ID	Ho	Hs	Ht	Dst	Htp	Dstp	Fst	Fstp	Fis	Dest
10	114859463	rs7085532	0,4230	0,4201	0,4194	-0,0007	0,4187	-0,0013	**-0,0016**	**-0,0032**	-0,0071	-0,0023
10	114898093	rs3814573	0,4779	0,4817	0,4808	-0,0008	0,4800	-0,0017	**-0,0017**	**-0,0035**	0,0078	-0,0032
10	114912534	rs1555485	0,1476	0,1627	0,1670	0,0043	0,1714	0,0086	**0,0258**	**0,0503**	0,0931	0,0103
11	2528003	rs11023096	0,3203	0,3102	0,3096	-0,0006	0,3090	-0,0013	**-0,0020**	**-0,0041**	-0,0325	-0,0018
11	2528233	rs4929992	0,4691	0,4947	0,4958	0,0011	0,4968	0,0021	**0,0021**	**0,0043**	0,0518	0,0042
11	2550730	rs179429	0,2624	0,2941	0,2944	0,0003	0,2947	0,0006	**0,0010**	**0,0020**	0,1078	0,0008
11	2553703	rs179435	0,3456	0,3870	0,3876	0,0007	0,3883	0,0013	**0,0017**	**0,0035**	0,1068	0,0022
11	2595287	rs2283171	0,4206	0,4490	0,4488	-0,0001	0,4487	-0,0003	**-0,0003**	**-0,0006**	0,0632	-0,0005
11	2617782	rs1116714	0,3497	0,3606	0,3701	0,0094	0,3795	0,0189	**0,0255**	**0,0497**	0,0303	0,0295
11	2633152	rs10766212	0,4622	0,5006	0,5009	0,0002	0,5011	0,0005	**0,0005**	**0,0009**	0,0768	0,0009
11	2635797	rs2106467	0,4652	0,4895	0,4913	0,0017	0,4930	0,0034	**0,0035**	**0,0070**	0,0497	0,0067
11	2673575	rs6578283	0,4238	0,4656	0,4654	-0,0002	0,4652	-0,0004	**-0,0005**	**-0,0009**	0,0899	-0,0008
11	2750703	rs170786	0,4006	0,4367	0,4410	0,0042	0,4452	0,0084	**0,0096**	**0,0190**	0,0828	0,0150
11	2776448	rs11023996	0,1421	0,1601	0,1605	0,0004	0,1609	0,0008	**0,0025**	**0,0049**	0,1121	0,0009
11	2782648	rs548566	0,2312	0,2739	0,2732	-0,0007	0,2726	-0,0013	**-0,0024**	**-0,0048**	0,1559	-0,0018
11	2821065	rs163171	0,3083	0,3290	0,3300	0,0011	0,3311	0,0022	**0,0033**	**0,0065**	0,0629	0,0032
11	2837625	rs233446	0,3474	0,3440	0,3452	0,0012	0,3464	0,0024	**0,0035**	**0,0070**	-0,0100	0,0037
11	2850782	rs234852	0,4090	0,4344	0,4407	0,0062	0,4469	0,0125	**0,0141**	**0,0279**	0,0585	0,0220
11	2895800	rs3987740	0,3840	0,4209	0,4245	0,0035	0,4280	0,0071	**0,0083**	**0,0165**	0,0877	0,0122
11	17393644	rs12791318	0,3446	0,3336	0,3399	0,0063	0,3462	0,0126	**0,0186**	**0,0365**	-0,0330	0,0189
11	17405333	rs10832785	0,4160	0,4702	0,4809	0,0107	0,4916	0,0214	**0,0223**	**0,0436**	0,1152	0,0405
11	17408025	rs2285676	0,4304	0,4761	0,4833	0,0072	0,4905	0,0145	**0,0150**	**0,0295**	0,0959	0,0276
11	17408630	rs5215	0,2737	0,3299	0,3387	0,0089	0,3476	0,0177	**0,0261**	**0,0509**	0,1704	0,0264
11	17408831	rs1800467	0,0640	0,0706	0,0708	0,0003	0,0711	0,0005	**0,0037**	**0,0073**	0,0931	0,0006
11	17438890	rs2074315	0,3013	0,3120	0,3113	-0,0007	0,3106	-0,0014	**-0,0022**	**-0,0045**	0,0340	-0,0020
11	17441828	rs4757517	0,3819	0,3862	0,3856	-0,0005	0,3851	-0,0010	**-0,0013**	**-0,0027**	0,0111	-0,0017
11	17496516	rs1048099	0,5121	0,4846	0,4842	-0,0004	0,4838	-0,0008	**-0,0008**	**-0,0017**	-0,0569	-0,0016
11	17510419	rs11603988	0,1809	0,1649	0,1649	0,0000	0,1649	0,0000	**-0,0001**	**-0,0002**	-0,0972	0,0000
11	17510565	rs4757527	0,3233	0,3998	0,4014	0,0016	0,4030	0,0032	**0,0040**	**0,0081**	0,1914	0,0054
11	17530484	rs7104083	0,4747	0,4875	0,4864	-0,0011	0,4853	-0,0021	**-0,0022**	**-0,0043**	0,0262	-0,0041
11	17532597	rs1076311	0,4868	0,4872	0,4864	-0,0008	0,4856	-0,0016	**-0,0016**	**-0,0033**	0,0008	-0,0031
11	17542649	rs2041032	0,4862	0,4997	0,4987	-0,0010	0,4977	-0,0020	**-0,0020**	**-0,0040**	0,0269	-0,0040
11	108097333	rs228591	0,3919	0,4471	0,4476	0,0005	0,4481	0,0010	**0,0012**	**0,0023**	0,1236	0,0019
11	108268286	rs7931930	0,4157	0,4639	0,4737	0,0099	0,4836	0,0197	**0,0208**	**0,0407**	0,1039	0,0368
11	108283161	rs11212617	0,3869	0,4511	0,4525	0,0015	0,4540	0,0029	**0,0032**	**0,0064**	0,1423	0,0053
16	31102321	rs7294	0,3665	0,3960	0,4020	0,0060	0,4080	0,0120	**0,0150**	**0,0295**	0,0745	0,0199
17	19447016	rs2440155	0,2761	0,2736	0,2760	0,0024	0,2785	0,0049	**0,0088**	**0,0175**	-0,0091	0,0067
17	19459537	rs2244280	0,2568	0,2893	0,2912	0,0019	0,2932	0,0039	**0,0066**	**0,0132**	0,1123	0,0054
17	19484951	rs2453594	0,2800	0,3245	0,3238	-0,0008	0,3230	-0,0015	**-0,0023**	**-0,0047**	0,1374	-0,0022
17	19622643	rs11656096	0,3067	0,3240	0,3500	0,0260	0,3760	0,0520	**0,0743**	**0,1384**	0,0532	0,0770
17	19642952	rs2228100	0,3985	0,4182	0,4220	0,0039	0,4259	0,0077	**0,0092**	**0,0182**	0,0470	0,0133
17	19645938	rs887241	0,4386	0,4749	0,4838	0,0089	0,4926	0,0177	**0,0183**	**0,0360**	0,0765	0,0338
22	42152988	rs17377643	0,4625	0,4631	0,4710	0,0078	0,4788	0,0157	**0,0167**	**0,0328**	0,0014	0,0292
22	42178441	rs126092	0,3455	0,3805	0,3841	0,0036	0,3877	0,0072	**0,0093**	**0,0185**	0,0920	0,0115
22	46235677	rs1023470	0,2776	0,3468	0,3500	0,0033	0,3533	0,0066	**0,0094**	**0,0186**	0,1994	0,0100
22	46238069	rs8141212	0,4426	0,4786	0,4819	0,0033	0,4852	0,0065	**0,0067**	**0,0134**	0,0752	0,0125
22	46525794	rs6007919	0,3584	0,4413	0,4405	-0,0008	0,4397	-0,0016	**-0,0018**	**-0,0037**	0,1880	-0,0029
22	46629479	rs4253776	0,2690	0,2831	0,2825	-0,0006	0,2819	-0,0012	**-0,0021**	**-0,0043**	0,0499	-0,0017
22	46637254	rs9626814	0,2469	0,2769	0,2763	-0,0005	0,2758	-0,0011	**-0,0020**	**-0,0040**	0,1081	-0,0015
22	46643774	rs16995069	0,1649	0,1720	0,1717	-0,0002	0,1715	-0,0005	**-0,0014**	**-0,0028**	0,0412	-0,0006
22	46670394	rs6007761	0,2720	0,3060	0,3072	0,0011	0,3083	0,0023	**0,0037**	**0,0073**	0,1112	0,0033

	**$overall**	**Ho**	**Hs**	**Ht**	**Dst**	**Htp**	**Dstp**	**Fst**	**Fstp**	**Fis**	**Dest**
	0,3419	0,3630	0,3651	0,0022	0,3663	0,0044	0,0059	**0,0119**	**0,0580**	0,0068	

$FST 0,0117

$FIS 0,0599

Supplementary Table 2 next

Fst Comparison among Tunisian and Iberian Italian populations

Chr	Localisation	rs ID	Ho	Hs	Ht	Dst	Htp	Dstp	Fst	Fstp	Fis	Dest
1	65381861	rs12563017	0,1465	0,1899	0,1895	-0,0004	0,1891	-0,0008	**-0,0022**	**-0,0044**	0,2290	-0,0010
1	65389835	rs10889503	0,2916		0,3492	0,0066	0,3558	0,0133	**0,0190**	**0,0373**	0,1488	0,0202
1	65421058	rs4916014	0,2903	0,4117	0,4119	0,0002	0,4121	0,0004	**0,0004**	**0,0009**	0,2949	0,0006
1	65427476	rs4915675		0,3695	0,3686	-0,0009	0,3677	-0,0019	**-0,0025**	**-0,0051**	0,2066	-0,0030
1	65516055	rs6588109	0,3628	0,3911	0,3913	0,0002	0,3915	0,0003	**0,0004**	**0,0009**	0,0726	0,0006
1	65557876	rs6699671	0,2196	0,2103	0,2134	0,0031	0,2164	0,0061	**0,0143**	**0,0282**	-0,0440	0,0077
1	65583858	rs11208591	0,5004	0,4847	0,4876	0,0030	0,4906	0,0059	**0,0061**	**0,0121**	-0,0325	0,0115
1	65619880	rs10789171	0,3711	0,3465	0,3461	-0,0004	0,3457	-0,0008	**-0,0012**	**-0,0024**	-0,0710	-0,0013
1	65658412	rs6677316	0,4321	0,3859	0,3853	-0,0006	0,3847	-0,0012	**-0,0016**	**-0,0032**	-0,1198	-0,0020
3	12286720	rs9850825	0,4618	0,4824	0,4925	0,0101	0,5026	0,0202	**0,0205**	**0,0402**	0,0427	0,0391
3	12302462	rs9878908	0,2279	0,2376	0,2415	0,0039	0,2453	0,0077	**0,0160**	**0,0315**	0,0409	0,0101
3	12393125	rs1801282	0,1765	0,1665	0,1674	0,0009	0,1683	0,0018	**0,0054**	**0,0107**	-0,0603	0,0022
3	12402474	rs1373641	0,3908	0,4140	0,4154	0,0014	0,4168	0,0028	**0,0033**	**0,0067**	0,0560	0,0047
3	12475088	rs7626560	0,2650	0,2644	0,2648	0,0003	0,2651	0,0007	**0,0013**	**0,0026**	-0,0021	0,0009
3	151007310	rs9863983	0,3092	0,3050	0,3047	-0,0003	0,3045	-0,0005	**-0,0008**	**-0,0017**	-0,0138	-0,0007
3	151041513	rs3971191	0,2282	0,2076	0,2097	0,0021	0,2118	0,0042	**0,0101**	**0,0200**	-0,0992	0,0053
3	151053898	rs7644001	0,4427	0,4877	0,4867	-0,0010	0,4857	-0,0020	**-0,0021**	**-0,0042**	0,0923	-0,0040
3	151090963	rs9859538	0,4736	0,4990	0,5007	0,0017	0,5024	0,0034	**0,0034**	**0,0068**	0,0509	0,0068
3	151112568	rs3732768	0,3171	0,3399	0,3391	-0,0008	0,3382	-0,0017	**-0,0024**	**-0,0049**	0,0670	-0,0025
3	151128895	rs10935844	0,4707	0,4990	0,5011	0,0021	0,5032	0,0042	**0,0042**	**0,0083**	0,0568	0,0084
3	151147968	rs6772196	0,1674	0,1726	0,1725	-0,0001	0,1723	-0,0003	**-0,0008**	**-0,0016**	0,0299	-0,0003
5	51405600	rs12655411	0,4685	0,4500	0,4511	0,0011	0,4522	0,0022	**0,0024**	**0,0048**	-0,0410	0,0039
5	51431680	rs10064799	0,4571	0,4471	0,4543	0,0072	0,4615	0,0144	**0,0159**	**0,0313**	-0,0223	0,0261
5	51505665	rs6865397	0,4799	0,4889	0,4878	-0,0011	0,4868	-0,0021	**-0,0022**	**-0,0043**	0,0182	-0,0041
5	51572584	rs4572960	0,4815	0,4803	0,4803	0,0000	NA	NA	**0,0000**	**NaN**	-0,0025	NA
5	74616843	rs10474433	0,4500	0,4770	0,4774	0,0004	0,4778	0,0007	**0,0008**	**0,0015**	0,0566	0,0014
5	74620912	rs6878576	0,2000	0,2278	0,2274	-0,0004	0,2270	-0,0008	**-0,0018**	**-0,0035**	0,1219	-0,0010
5	74625487	rs7703051	0,4164	0,4433	0,4436	0,0002	0,4438	0,0004	**0,0005**	**0,0010**	0,0607	0,0008
5	74648603	rs12654264	0,4142	0,4457	0,4456	-0,0001	0,4455	-0,0002	**-0,0002**	**-0,0003**	0,0707	-0,0003
5	74651084	rs3846662	0,4434	0,4932	0,4948	0,0016	0,4965	0,0033	**0,0033**	**0,0066**	0,1011	0,0065
5	74655726	rs3846663	0,4053	0,4448	0,4449	0,0000	0,4449	0,0000	**0,0000**	**0,0001**	0,0890	0,0001
5	74656175	rs5909	0,1443	0,1625	0,1625	0,0000	0,1625	0,0000	**0,0000**	**0,0000**	0,1124	0,0000
6	160496055	rs3777406	0,2628	0,2632	0,2651	0,0019	0,2669	0,0037	**0,0070**	**0,0139**	0,0016	0,0050
6	160517481	rs1803989	0,1494	0,1441	0,1448	0,0007	0,1455	0,0014	**0,0049**	**0,0097**	-0,0364	0,0016
6	160528057	rs7753051	0,3415	0,3557	0,3553	-0,0004	0,3549	-0,0008	**-0,0011**	**-0,0022**	0,0399	-0,0012
6	160572866	rs622342	0,4492	0,4230	0,4461	0,0231	0,4692	0,0462	**0,0517**	**0,0984**	-0,0618	0,0800
6	160578860	rs1564348	0,2837	0,2858	0,2851	-0,0006	0,2845	-0,0012	**-0,0022**	**-0,0044**	0,0071	-0,0017
6	160581374	rs651164	0,4566	0,4546	0,4588	0,0043	0,4631	0,0086	**0,0093**	**0,0185**	-0,0044	0,0157
6	160582340	rs9456505	0,1406	0,1376	0,1374	-0,0001	0,1373	-0,0003	**-0,0010**	**-0,0020**	-0,0221	-0,0003
6	160635886	rs10945656	0,2127	0,2298	0,2292	-0,0005	0,2287	-0,0010	**-0,0023**	**-0,0046**	0,0745	-0,0014
6	160637239	rs596881	0,2042	0,2427	0,2446	0,0018	0,2464	0,0036	**0,0074**	**0,0147**	0,1589	0,0048
6	160672625	rs316013	0,3987	0,4004	0,4012	0,0009	0,4021	0,0017	**0,0021**	**0,0043**	0,0043	0,0029
6	160681393	rs3127573	0,1781	0,1983	0,1981	-0,0003	0,1978	-0,0005	**-0,0013**	**-0,0026**	0,1018	-0,0007
6	160682897	rs7757997	0,1596	0,1635	0,1672	0,0037	0,1710	0,0075	**0,0223**	**0,0436**	0,0238	0,0089
6	160687866	rs316030	0,4539	0,4324	0,4356	0,0032	0,4388	0,0064	**0,0073**	**0,0145**	-0,0497	0,0112
6	160699534	rs2619276	0,3102	0,3420	0,3417	-0,0003	0,3414	-0,0006	**-0,0009**	**-0,0018**	0,0929	-0,0009
7	87103670	rs2888611	0,3697	0,3647	0,3665	0,0017	0,3682	0,0035	**0,0047**	**0,0094**	-0,0137	0,0054
7	87154646	rs10225473	0,2583	0,2564	0,2560	-0,0004	0,2556	-0,0008	**-0,0016**	**-0,0031**	-0,0075	-0,0011
7	87163016	rs11760837	0,2358	0,2402	0,2408	0,0006	0,2414	0,0012	**0,0025**	**0,0050**	0,0184	0,0016
7	87179143	rs2235033	0,5005	0,4975	0,4970	-0,0006	0,4964	-0,0011	**-0,0011**	**-0,0022**	-0,0060	-0,0022
7	87179809	rs2229109	0,0577	0,0632	0,0631	-0,0001	0,0630	-0,0002	**-0,0016**	**-0,0033**	0,0876	-0,0002
7	87180198	rs10276036	0,5015	0,4751	0,4742	-0,0009	0,4733	-0,0018	**-0,0019**	**-0,0037**	-0,0555	-0,0034
7	87183354	rs1922240	0,4526	0,4574	0,4565	-0,0008	0,4557	-0,0016	**-0,0018**	**-0,0036**	0,0104	-0,0030
7	87201482	rs10260862	0,3522	0,3481	0,3475	-0,0006	0,3469	-0,0012	**-0,0018**	**-0,0036**	-0,0117	-0,0019
7	87278760	rs10267099	0,3931	0,3772	0,3765	-0,0007	0,3757	-0,0015	**-0,0020**	**-0,0039**	-0,0421	-0,0024
7	99207876	rs7792939	0,1414	0,1742	0,1755	0,0013	0,1768	0,0025	**0,0073**	**0,0144**	0,1883	0,0031
10	96581094	rs10786172	0,3947	0,3997	0,4009	0,0012	0,4021	0,0024	**0,0030**	**0,0060**	0,0125	0,0040
10	114711983	rs7094463	0,4921	0,4877	0,4892	0,0015	0,4907	0,0029	**0,0030**	**0,0060**	-0,0090	0,0057
10	114732906	rs7901275	0,4810	0,4891	0,4905	0,0014	0,4919	0,0028	**0,0029**	**0,0058**	0,0165	0,0055
10	114754088	rs7901695	0,4804	0,4853	0,4875	0,0022	0,4897	0,0044	**0,0045**	**0,0089**	0,0102	0,0085
10	114756041	rs4506565	0,4742	0,4859	0,4879	0,0020	0,4899	0,0040	**0,0041**	**0,0081**	0,0242	0,0077
10	114767771	rs4132670	0,4619	0,4963	0,4954	-0,0009	0,4945	-0,0017	**-0,0017**	**-0,0035**	0,0693	-0,0034
10	114788815	rs12243326	0,4168	0,4646	0,4636	-0,0010	0,4626	-0,0020	**-0,0022**	**-0,0044**	0,1029	-0,0038
10	114821249	rs11196212	0,4510	0,4917	0,4940	0,0022	0,4962	0,0045	**0,0046**	**0,0091**	0,0828	0,0088
10	114855397	rs11196224	0,4669	0,4728	0,4783	0,0055	0,4838	0,0109	**0,0114**	**0,0226**	0,0126	0,0208

Supplementary Table 2 next

Chr	Localisation	rs ID	Ho	Hs	Ht	Dst	Htp	Dstp	Fst	Fstp	Fis	Dest
10	114859463	rs7085532	0,3716	0,4288	0,4287	-0,0001	0,4286	-0,0001	**-0,0001**	**-0,0003**	0,1332	-0,0002
10	114898093	rs3814573	0,4499	0,4686	0,4680	-0,0006	0,4674	-0,0012	**-0,0013**	**-0,0026**	0,0399	-0,0023
10	114912534	rs1555485	0,1569	0,1694	0,1746	0,0053	0,1799	0,0105	**0,0301**	**0,0584**	0,0736	0,0126
11	2528003	rs11023096	0,3296	0,3215	0,3210	-0,0005	0,3205	-0,0009	**-0,0014**	**-0,0029**	-0,0255	-0,0014
11	2528233	rs4929992	0,5111	0,4949	0,4970	0,0022	0,4992	0,0044	**0,0044**	**0,0087**	-0,0329	0,0086
11	2550730	rs179429	0,3091	0,3048	0,3062	0,0013	0,3075	0,0027	**0,0044**	**0,0088**	-0,0141	0,0039
11	2553703	rs179435	0,3924	0,4176	0,4167	-0,0009	0,4157	-0,0019	**-0,0022**	**-0,0045**	0,0604	-0,0032
11	2595287	rs2283171	0,4440	0,4598	0,4589	-0,0010	0,4579	-0,0019	**-0,0021**	**-0,0042**	0,0345	-0,0035
11	2617782	rs1116714	0,3544	0,3637	0,3724	0,0088	0,3812	0,0175	**0,0235**	**0,0460**	0,0256	0,0276
11	2633152	rs10766212	0,4575	0,5021	0,5011	-0,0010	0,5001	-0,0019	**-0,0019**	**-0,0038**	0,0887	-0,0038
11	2635797	rs2106467	0,4559	0,4901	0,4925	0,0024	0,4950	0,0049	**0,0049**	**0,0098**	0,0699	0,0095
11	2673575	rs6578283	0,4845	0,4721	0,4713	-0,0008	0,4705	-0,0016	**-0,0017**	**-0,0034**	-0,0263	-0,0031
11	2750703	rs170786	0,4240	0,4387	0,4425	0,0038	0,4463	0,0076	**0,0086**	**0,0170**	0,0337	0,0135
11	2776448	rs11023996	0,1515	0,1745	0,1757	0,0013	0,1770	0,0025	**0,0072**	**0,0143**	0,1319	0,0031
11	2782648	rs548566	0,2499	0,3038	0,3038	0,0000	0,3037	0,0000	**0,0000**	**-0,0001**	0,1774	0,0000
11	2821065	rs163171	0,3550	0,3621	0,3613	-0,0008	0,3605	-0,0016	**-0,0022**	**-0,0044**	0,0195	-0,0025
11	2837625	rs233446	0,3474	0,3530	0,3555	0,0025	0,3580	0,0050	**0,0071**	**0,0141**	0,0157	0,0078
11	2850782	rs234852	0,3997	0,4345	0,4407	0,0062	0,4469	0,0124	**0,0141**	**0,0278**	0,0801	0,0220
11	2895800	rs3987740	0,4307	0,4485	0,4479	-0,0005	0,4474	-0,0011	**-0,0012**	**-0,0024**	0,0395	-0,0020
11	17393644	rs12791318	0,3166	0,3203	0,3236	0,0034	0,3270	0,0068	**0,0104**	**0,0207**	0,0115	0,0099
11	17405333	rs10832785	0,4394	0,4684	0,4799	0,0115	0,4914	0,0230	**0,0240**	**0,0468**	0,0619	0,0433
11	17408025	rs2285676	0,4631	0,4712	0,4805	0,0092	0,4897	0,0184	**0,0192**	**0,0377**	0,0172	0,0349
11	17408630	rs5215	0,3157	0,3625	0,3898	0,0273	0,4172	0,0546	**0,0701**	**0,1309**	0,1291	0,0857
11	17408831	rs1800467	0,0780	0,0830	0,0837	0,0007	0,0845	0,0015	**0,0089**	**0,0177**	0,0601	0,0016
11	17438890	rs2074315	0,3387	0,3482	0,3495	0,0013	0,3508	0,0026	**0,0037**	**0,0073**	0,0272	0,0039
11	17441828	rs4757517	0,4473	0,4216	0,4225	0,0009	0,4234	0,0019	**0,0022**	**0,0044**	-0,0611	0,0032
11	17496516	rs1048099	0,4748	0,4890	0,4904	0,0015	0,4919	0,0029	**0,0030**	**0,0059**	0,0291	0,0057
11	17510419	rs11603988	0,2370	0,2307	0,2372	0,0065	0,2437	0,0130	**0,0274**	**0,0533**	-0,0272	0,0169
11	17510565	rs4757527	0,3560	0,3862	0,3861	-0,0001	0,3861	-0,0002	**-0,0002**	**-0,0005**	0,0783	-0,0003
11	17530484	rs7104083	0,4700	0,4944	0,4960	0,0016	0,4976	0,0031	**0,0032**	**0,0063**	0,0493	0,0062
11	17532597	rs1076311	0,4681	0,4925	0,4945	0,0019	0,4964	0,0039	**0,0039**	**0,0078**	0,0496	0,0076
11	17542649	rs2041032	0,4302	0,4985	0,5012	0,0027	0,5039	0,0054	**0,0054**	**0,0107**	0,1371	0,0107
11	108097333	rs228591	0,4526	0,4553	0,4549	-0,0004	0,4545	-0,0008	**-0,0009**	**-0,0019**	0,0060	-0,0016
11	108268286	rs7931930	0,4858	0,4751	0,4807	0,0056	0,4864	0,0113	**0,0117**	**0,0232**	-0,0224	0,0215
11	108283161	rs11212617	0,4570	0,4623	0,4621	-0,0002	0,4618	-0,0005	**-0,0005**	**-0,0011**	0,0116	-0,0009
16	31102321	rs7294	0,3478	0,4019	0,4102	0,0083	0,4185	0,0166	**0,0203**	**0,0398**	0,1346	0,0278
17	19447016	rs2440155	0,2621	0,2629	0,2665	0,0036	0,2702	0,0073	**0,0137**	**0,0269**	0,0031	0,0099
17	19459537	rs2244280	0,2568	0,3025	0,3032	0,0007	0,3039	0,0014	**0,0023**	**0,0046**	0,1511	0,0020
17	19484951	rs2453594	0,2753	0,3097	0,3096	-0,0001	0,3094	-0,0003	**-0,0004**	**-0,0009**	0,1111	-0,0004
17	19622643	rs11656096	0,3114	0,3254	0,3526	0,0271	0,3797	0,0542	**0,0769**	**0,1428**	0,0431	0,0804
17	19642952	rs2228100	0,3892	0,4034	0,4105	0,0071	0,4175	0,0141	**0,0172**	**0,0338**	0,0353	0,0236
17	19645938	rs887241	0,4619	0,4845	0,4893	0,0048	0,4941	0,0096	**0,0098**	**0,0194**	0,0466	0,0186
22	42152988	rs17377643	0,4578	0,4599	0,4650	0,0050	0,4700	0,0100	**0,0108**	**0,0214**	0,0047	0,0186
22	42178441	rs126092	0,3315	0,3886	0,3908	0,0023	0,3931	0,0045	**0,0058**	**0,0115**	0,1469	0,0074
22	46235677	rs1023470	0,2636	0,3256	0,3260	0,0004	0,3264	0,0008	**0,0012**	**0,0023**	0,1905	0,0011
22	46238069	rs8141212	0,4567	0,4741	0,4749	0,0008	0,4757	0,0016	**0,0017**	**0,0033**	0,0368	0,0030
22	46525794	rs6007919	0,3724	0,4319	0,4320	0,0001	0,4322	0,0003	**0,0003**	**0,0006**	0,1378	0,0005
22	46629479	rs4253776	0,1943	0,2360	0,2371	0,0012	0,2383	0,0023	**0,0049**	**0,0098**	0,1768	0,0031
22	46637254	rs9626814	0,1722	0,2297	0,2303	0,0006	0,2309	0,0012	**0,0027**	**0,0053**	0,2504	0,0016
22	46643774	rs16995069	0,1368	0,1488	0,1486	-0,0003	0,1483	-0,0005	**-0,0018**	**-0,0036**	0,0806	-0,0006
22	46670394	rs6007761	0,2159	0,2714	0,2707	-0,0007	0,2699	-0,0014	**-0,0026**	**-0,0053**	0,2044	-0,0020

$overall	Ho	Hs	Ht	Dst	Htp	Dstp	Fst	Fstp	Fis	Dest
	0,3517	0,368	0,3702	0,0022	0,3715	0,0044	**0,0059**	**0,0118**	0,0445	0,007

$FST 0,0117

$FIS 0,04774

Supplementary Table 2 next

Fst Comparison among Tunisian and Utah residents (CEPH) with Northern and Western European ancestry populations

Chr	Localisation	rs ID	Ho	Hs	Ht	Dst	Htp	Dstp	Fst	Fstp	Fis	Dest
1	65381861	rs12563017	0,1750	0,2119	0,2122	0,0003	0,2126	0,0007	**0,0016**	**0,0031**	0,1743	0,0008
1	65389835	rs10889503	0,3523		0,3872	0,0002	0,3874	0,0005	**0,0006**	**0,0012**	0,0896	0,0008
1	65421058	rs4916014	0,3680	0,4316	0,4304	-0,0012	0,4292	-0,0024	**-0,0028**	**-0,0056**	0,1472	-0,0042
1	65427476	rs4915675		0,3838	0,3834	-0,0004	0,3830	-0,0009	**-0,0011**	**-0,0022**	0,0488	-0,0014
1	65516055	rs6588109	0,3923	0,4079	0,4070	-0,0009	0,4062	-0,0017	**-0,0021**	**-0,0043**	0,0384	-0,0029
1	65557876	rs6699671	0,2260	0,2401	0,2407	0,0005	0,2412	0,0011	**0,0022**	**0,0044**	0,0588	0,0014
1	65583858	rs11208591	0,5539	0,4774	0,4831	0,0056	0,4887	0,0113	**0,0117**	**0,0231**	-0,1602	0,0216
1	65619880	rs10789171	0,3135	0,3541	0,3539	-0,0002	0,3537	-0,0004	**-0,0006**	**-0,0011**	0,1147	-0,0006
1	65658412	rs6677316	0,3702	0,3863	0,3855	-0,0008	0,3848	-0,0016	**-0,0020**	**-0,0041**	0,0418	-0,0026
3	12286720	rs9850825	0,4306	0,4761	0,4767	0,0006	0,4773	0,0012	**0,0013**	**0,0025**	0,0957	0,0023
3	12302462	rs9878908	0,2147	0,2377	0,2415	0,0038	0,2453	0,0076	**0,0158**	**0,0310**	0,0966	0,0100
3	12393125	rs1801282	0,1355	0,1501	0,1502	0,0001	0,1502	0,0001	**0,0004**	**0,0007**	0,0977	0,0001
3	12402474	rs1373641	0,3519	0,4110	0,4118	0,0007	0,4125	0,0014	**0,0017**	**0,0034**	0,1440	0,0024
3	12475088	rs7626560	0,2593	0,2614	0,2619	0,0005	0,2624	0,0010	**0,0019**	**0,0038**	0,0082	0,0014
3	151007310	rs9863983	0,3239	0,3256	0,3269	0,0013	0,3283	0,0027	**0,0041**	**0,0082**	0,0053	0,0040
3	151041513	rs3971191	0,2601	0,2416	0,2487	0,0072	0,2559	0,0143	**0,0288**	**0,0560**	-0,0767	0,0189
3	151053898	rs7644001	0,4561	0,4718	0,4739	0,0021	0,4760	0,0042	**0,0044**	**0,0088**	0,0333	0,0079
3	151090963	rs9859538	0,4976	0,4970	0,5011	0,0041	0,5053	0,0083	**0,0082**	**0,0164**	-0,0012	0,0164
3	151112568	rs3732768	0,3058	0,3236	0,3229	-0,0006	0,3223	-0,0013	**-0,0020**	**-0,0040**	0,0547	-0,0019
3	151128895	rs10935844	0,4931	0,4982	0,5010	0,0028	0,5039	0,0056	**0,0056**	**0,0112**	0,0103	0,0112
3	151147968	rs6772196	0,1641	0,1699	0,1698	-0,0001	0,1697	-0,0001	**-0,0004**	**-0,0008**	0,0337	-0,0002
5	51405600	rs12655411	0,4419	0,4365	0,4358	-0,0008	0,4350	-0,0016	**-0,0018**	**-0,0037**	-0,0123	-0,0028
5	51431680	rs10064799	0,4572	0,4551	0,4765	0,0213	0,4978	0,0427	**0,0448**	**0,0858**	-0,0047	0,0783
5	51505665	rs6865397	0,4899	0,4924	0,4914	-0,0009	0,4905	-0,0019	**-0,0019**	**-0,0039**	0,0051	-0,0037
5	51572584	rs4572960	0,4815	0,4803	0,4803	0,0000	NA	NA	**0,0000**	**NaN**	-0,0025	NA
5	74616843	rs10474433	0,4911	0,4781	0,4783	0,0002	0,4784	0,0004	**0,0004**	**0,0008**	-0,0273	0,0007
5	74620912	rs6878576	0,2126	0,2157	0,2158	0,0001	0,2158	0,0001	**0,0002**	**0,0005**	0,0145	0,0001
5	74625487	rs7703051	0,4338	0,4532	0,4560	0,0028	0,4588	0,0057	**0,0062**	**0,0123**	0,0427	0,0104
5	74648603	rs12654264	0,4316	0,4555	0,4578	0,0023	0,4601	0,0046	**0,0050**	**0,0100**	0,0526	0,0084
5	74651084	rs3846662	0,4755	0,4962	0,4967	0,0005	0,4972	0,0010	**0,0010**	**0,0019**	0,0417	0,0019
5	74655726	rs3846663	0,4176	0,4536	0,4557	0,0021	0,4577	0,0041	**0,0045**	**0,0090**	0,0794	0,0075
5	74656175	rs5909	0,1763	0,1807	0,1816	0,0009	0,1825	0,0018	**0,0049**	**0,0098**	0,0241	0,0022
6	160496055	rs3777406	0,2508	0,2571	0,2583	0,0012	0,2595	0,0024	**0,0046**	**0,0092**	0,0249	0,0032
6	160517481	rs1803989	0,1286	0,1346	0,1358	0,0012	0,1370	0,0024	**0,0090**	**0,0178**	0,0443	0,0028
6	160528057	rs7753051	0,3212	0,3512	0,3511	-0,0002	0,3509	-0,0004	**-0,0005**	**-0,0010**	0,0855	-0,0005
6	160572866	rs622342	0,3863	0,4129	0,4245	0,0116	0,4362	0,0232	**0,0273**	**0,0532**	0,0644	0,0396
6	160578860	rs1564348	0,2803	0,2783	0,2777	-0,0006	0,2771	-0,0012	**-0,0021**	**-0,0042**	-0,0073	-0,0016
6	160581374	rs651164	0,4930	0,4670	0,4683	0,0013	0,4696	0,0026	**0,0028**	**0,0056**	-0,0557	0,0049
6	160582340	rs9456505	0,1459	0,1422	0,1420	-0,0002	0,1417	-0,0005	**-0,0016**	**-0,0033**	-0,0261	-0,0005
6	160635886	rs10945656	0,1907	0,2068	0,2068	0,0000	0,2068	0,0000	**0,0000**	**0,0001**	0,0780	0,0000
6	160637239	rs596881	0,2164	0,2534	0,2543	0,0009	0,2552	0,0018	**0,0036**	**0,0071**	0,1461	0,0024
6	160672625	rs316013	0,3932	0,3897	0,3919	0,0022	0,3942	0,0045	**0,0057**	**0,0114**	-0,0090	0,0074
6	160681393	rs3127573	0,1806	0,1863	0,1859	-0,0004	0,1855	-0,0008	**-0,0023**	**-0,0046**	0,0305	-0,0010
6	160682897	rs7757997	0,1475	0,1435	0,1494	0,0059	0,1554	0,0119	**0,0398**	**0,0765**	-0,0278	0,0139
6	160687866	rs316030	0,4339	0,4398	0,4459	0,0062	0,4521	0,0123	**0,0138**	**0,0272**	0,0133	0,0220
6	160699534	rs2619276	0,3562	0,3590	0,3603	0,0013	0,3617	0,0027	**0,0037**	**0,0074**	0,0079	0,0042
7	87103670	rs2888611	0,3278	0,3403	0,3455	0,0052	0,3507	0,0104	**0,0151**	**0,0297**	0,0369	0,0158
7	87154646	rs10225473	0,2444	0,2405	0,2400	-0,0005	0,2394	-0,0010	**-0,0022**	**-0,0043**	-0,0162	-0,0014
7	87163016	rs11760837	0,2172	0,2211	0,2208	-0,0003	0,2205	-0,0006	**-0,0014**	**-0,0028**	0,0178	-0,0008
7	87179143	rs2235033	0,5259	0,4986	0,5008	0,0022	0,5030	0,0044	**0,0044**	**0,0087**	-0,0547	0,0087
7	87179809	rs2229109	0,0448	0,0508	0,0510	0,0001	0,0511	0,0002	**0,0023**	**0,0045**	0,1191	0,0002
7	87180198	rs10276036	0,5316	0,4849	0,4847	-0,0002	0,4845	-0,0004	**-0,0004**	**-0,0008**	-0,0963	-0,0008
7	87183354	rs1922240	0,4365	0,4252	0,4267	0,0015	0,4282	0,0030	**0,0035**	**0,0070**	-0,0265	0,0052
7	87201482	rs10260862	0,3572	0,3632	0,3624	-0,0008	0,3616	-0,0016	**-0,0022**	**-0,0044**	0,0165	-0,0025
7	87278760	rs10267099	0,3985	0,3771	0,3764	-0,0008	0,3756	-0,0015	**-0,0020**	**-0,0041**	-0,0565	-0,0024
7	99207876	rs7792939	0,1637	0,1704	0,1714	0,0010	0,1725	0,0021	**0,0061**	**0,0121**	0,0390	0,0025
10	96581094	rs10786172	0,4431	0,4205	0,4280	0,0075	0,4355	0,0150	**0,0175**	**0,0344**	-0,0538	0,0258
10	114711983	rs7094463	0,5164	0,5002	0,4995	-0,0007	0,4987	-0,0014	**-0,0014**	**-0,0029**	-0,0325	-0,0029
10	114732906	rs7901275	0,4715	0,4869	0,5000	0,0131	0,5131	0,0262	**0,0262**	**0,0510**	0,0318	0,0510
10	114754088	rs7901695	0,3667	0,4580	0,4580	0,0000	0,4580	0,0000	**0,0000**	**0,0001**	0,1994	0,0001
10	114756041	rs4506565	0,3655	0,4603	0,4601	-0,0002	0,4599	-0,0003	**-0,0003**	**-0,0007**	0,2059	-0,0006
10	114767771	rs4132670	0,3626	0,4706	0,4745	0,0039	0,4784	0,0078	**0,0082**	**0,0163**	0,2295	0,0147
10	114788815	rs12243326	0,3106	0,4304	0,4327	0,0024	0,4351	0,0047	**0,0054**	**0,0108**	0,2783	0,0083
10	114821249	rs11196212	0,4147	0,4892	0,5002	0,0110	0,5112	0,0220	**0,0219**	**0,0429**	0,1523	0,0430
10	114855397	rs11196224	0,4539	0,4524	0,4654	0,0130	0,4784	0,0261	**0,0280**	**0,0545**	-0,0034	0,0476

Supplementary Table 2 next

Chr	Localisation	rs ID	Ho	Hs	Ht	Dst	Htp	Dstp	Fst	Fstp	Fis	Dest
10	114859463	rs7085532	0,4167	0,4200	0,4193	-0,0007	0,4186	-0,0015	**-0,0017**	**-0,0035**	0,0080	-0,0025
10	114898093	rs3814573	0,4839	0,4772	0,4761	-0,0011	0,4750	-0,0022	**-0,0024**	**-0,0047**	-0,0140	-0,0043
10	114912534	rs1555485	0,2021	0,2066	0,2195	0,0129	0,2324	0,0259	**0,0589**	**0,1113**	0,0217	0,0326
11	2528003	rs11023096	0,3300	0,3339	0,3341	0,0001	0,3342	0,0003	**0,0004**	**0,0008**	0,0118	0,0004
11	2528233	rs4929992	0,4883	0,4947	0,4956	0,0010	0,4966	0,0019	**0,0019**	**0,0039**	0,0128	0,0038
11	2550730	rs179429	0,2784	0,2886	0,2885	0,0000	0,2885	-0,0001	**-0,0001**	**-0,0003**	0,0353	-0,0001
11	2553703	rs179435	0,3340	0,3994	0,3989	-0,0005	0,3983	-0,0010	**-0,0013**	**-0,0026**	0,1637	-0,0017
11	2595287	rs2283171	0,3781	0,4210	0,4257	0,0047	0,4303	0,0094	**0,0110**	**0,0218**	0,1018	0,0162
11	2617782	rs1116714	0,3066	0,3163	0,3365	0,0202	0,3567	0,0405	**0,0601**	**0,1134**	0,0306	0,0592
11	2633152	rs10766212	0,4259	0,4743	0,4896	0,0153	0,5050	0,0307	**0,0313**	**0,0608**	0,1020	0,0584
11	2635797	rs2106467	0,4242	0,4629	0,4635	0,0007	0,4642	0,0013	**0,0014**	**0,0028**	0,0835	0,0025
11	2673575	rs6578283	0,4237	0,4304	0,4379	0,0074	0,4453	0,0149	**0,0170**	**0,0334**	0,0156	0,0261
11	2750703	rs170786	0,4492	0,4521	0,4532	0,0011	0,4543	0,0022	**0,0024**	**0,0048**	0,0066	0,0040
11	2776448	rs11023996	0,1854	0,1998	0,2037	0,0039	0,2076	0,0078	**0,0191**	**0,0375**	0,0722	0,0097
11	2782648	rs548566	0,2453	0,2723	0,2717	-0,0006	0,2710	-0,0012	**-0,0023**	**-0,0046**	0,0991	-0,0017
11	2821065	rs163171	0,3153	0,3473	0,3469	-0,0004	0,3466	-0,0007	**-0,0011**	**-0,0022**	0,0922	-0,0011
11	2837625	rs233446	0,3582	0,3605	0,3645	0,0040	0,3685	0,0080	**0,0109**	**0,0216**	0,0062	0,0125
11	2850782	rs234852	0,4116	0,4420	0,4463	0,0043	0,4506	0,0086	**0,0097**	**0,0192**	0,0688	0,0155
11	2895800	rs3987740	0,4182	0,4211	0,4247	0,0035	0,4282	0,0070	**0,0083**	**0,0164**	0,0070	0,0121
11	17393644	rs12791318	0,2664	0,2693	0,2687	-0,0006	0,2680	-0,0012	**-0,0023**	**-0,0045**	0,0106	-0,0017
11	17405333	rs10832785	0,4517	0,4871	0,4907	0,0036	0,4943	0,0072	**0,0073**	**0,0145**	0,0727	0,0140
11	17408025	rs2285676	0,4762	0,4890	0,4912	0,0022	0,4934	0,0044	**0,0045**	**0,0089**	0,0262	0,0086
11	17408630	rs5215	0,3556	0,3626	0,3901	0,0275	0,4176	0,0551	**0,0706**	**0,1318**	0,0194	0,0864
11	17408831	rs1800467	0,0573	0,0558	0,0558	0,0000	0,0557	-0,0001	**-0,0007**	**-0,0014**	-0,0264	-0,0001
11	17438890	rs2074315	0,3550	0,3485	0,3498	0,0013	0,3511	0,0026	**0,0038**	**0,0075**	-0,0187	0,0041
11	17441828	rs4757517	0,4580	0,4168	0,4170	0,0002	0,4172	0,0004	**0,0005**	**0,0011**	-0,0988	0,0008
11	17496516	rs1048099	0,5041	0,4902	0,4962	0,0060	0,5022	0,0119	**0,0120**	**0,0238**	-0,0283	0,0234
11	17510419	rs11603988	0,2347	0,2249	0,2304	0,0054	0,2358	0,0109	**0,0236**	**0,0461**	-0,0434	0,0140
11	17510565	rs4757527	0,3120	0,3629	0,3620	-0,0009	0,3611	-0,0018	**-0,0024**	**-0,0049**	0,1402	-0,0028
11	17530484	rs7104083	0,4154	0,4924	0,5006	0,0082	0,5088	0,0164	**0,0164**	**0,0323**	0,1563	0,0324
11	17532597	rs1076311	0,4232	0,4901	0,5004	0,0103	0,5107	0,0206	**0,0206**	**0,0404**	0,1364	0,0404
11	17542649	rs2041032	0,4176	0,4884	0,4896	0,0012	0,4908	0,0024	**0,0025**	**0,0049**	0,1450	0,0047
11	108097333	rs228591	0,5022	0,4812	0,4837	0,0026	0,4863	0,0052	**0,0053**	**0,0106**	-0,0437	0,0100
11	108268286	rs7931930	0,5260	0,4979	0,4968	-0,0011	0,4957	-0,0022	**-0,0023**	**-0,0045**	-0,0563	-0,0045
11	108283161	rs11212617	0,4972	0,4851	0,4865	0,0014	0,4879	0,0027	**0,0028**	**0,0056**	-0,0249	0,0053
16	31102321	rs7294	0,3550	0,3879	0,3914	0,0035	0,3949	0,0070	**0,0090**	**0,0178**	0,0846	0,0115
17	19447016	rs2440155	0,2956	0,3219	0,3212	-0,0007	0,3204	-0,0015	**-0,0023**	**-0,0046**	0,0816	-0,0022
17	19459537	rs2244280	0,2825	0,3503	0,3497	-0,0006	0,3490	-0,0013	**-0,0018**	**-0,0036**	0,1934	-0,0019
17	19484951	rs2453594	0,2668	0,3056	0,3057	0,0001	0,3058	0,0001	**0,0002**	**0,0005**	0,1271	0,0002
17	19622643	rs11656096	0,3043	0,3198	0,3429	0,0231	0,3660	0,0462	**0,0674**	**0,1263**	0,0484	0,0680
17	19642952	rs2228100	0,4101	0,4256	0,4281	0,0024	0,4305	0,0049	**0,0057**	**0,0113**	0,0365	0,0085
17	19645938	rs887241	0,4416	0,4722	0,4822	0,0100	0,4923	0,0200	**0,0208**	**0,0407**	0,0649	0,0380
22	42152988	rs17377643	0,4118	0,4492	0,4498	0,0006	0,4504	0,0012	**0,0013**	**0,0026**	0,0833	0,0022
22	42178441	rs126092	0,3350	0,3817	0,3850	0,0033	0,3884	0,0067	**0,0087**	**0,0172**	0,1224	0,0108
22	46235677	rs1023470	0,2710	0,3380	0,3397	0,0017	0,3415	0,0035	**0,0051**	**0,0102**	0,1982	0,0053
22	46238069	rs8141212	0,4744	0,4762	0,4779	0,0017	0,4796	0,0033	**0,0035**	**0,0070**	0,0038	0,0064
22	46525794	rs6007919	0,4279	0,4671	0,4681	0,0010	0,4690	0,0020	**0,0021**	**0,0042**	0,0839	0,0037
22	46629479	rs4253776	0,2360	0,2616	0,2613	-0,0003	0,2609	-0,0006	**-0,0012**	**-0,0024**	0,0976	-0,0008
22	46637254	rs9626814	0,2190	0,2589	0,2583	-0,0006	0,2577	-0,0012	**-0,0024**	**-0,0048**	0,1541	-0,0017
22	46643774	rs16995069	0,1270	0,1321	0,1322	0,0002	0,1324	0,0004	**0,0013**	**0,0027**	0,0385	0,0004
22	46670394	rs6007761	0,2413	0,2700	0,2693	-0,0007	0,2686	-0,0014	**-0,0026**	**-0,0051**	0,1065	-0,0019

$overall	Ho	Hs	Ht	Dst	Htp	Dstp	Fst	Fstp	Fis	Dest
	0,3502	0,3671	0,3700	0,0028	0,3719	0,0057	**0,0077**	**0,0154**	0,0461	0,0091

$FST 0,0153

$FIS 0,0503

Supplementary Table 2 next

Fst Comparison among Tunisian and British populations

Chr	Localisation	rs ID	Ho	Hs	Ht	Dst	Htp	Dstp	Fst	Fstp	Fis	Dest
1	65381861	rs12563017	0,1371	0,1840	0,1834	-0,0005	0,1829	-0,0011	**-0,0029**	**-0,0058**	0,2549	-0,0013
1	65389835	rs10889503	0,3248		0,3589	0,0046	0,3634	0,0091	**0,0127**	**0,0251**	0,0834	0,0141
1	65421058	rs4916014	0,3441	0,4152	0,4150	-0,0002	0,4149	-0,0004	**-0,0004**	**-0,0009**	0,1714	-0,0006
1	65427476	rs4915675		0,3717	0,3708	-0,0009	0,3699	-0,0019	**-0,0025**	**-0,0050**	0,1007	-0,0030
1	65516055	rs6588109	0,3885	0,4267	0,4262	-0,0004	0,4258	-0,0009	**-0,0010**	**-0,0021**	0,0895	-0,0016
1	65557876	rs6699671	0,2500	0,2352	0,2361	0,0009	0,2370	0,0018	**0,0038**	**0,0075**	-0,0632	0,0023
1	65583858	rs11208591	0,5563	0,4859	0,4884	0,0025	0,4910	0,0051	**0,0052**	**0,0103**	-0,1449	0,0099
1	65619880	rs10789171	0,3117	0,3512	0,3508	-0,0004	0,3504	-0,0009	**-0,0012**	**-0,0025**	0,1127	-0,0013
1	65658412	rs6677316	0,3688	0,3838	0,3830	-0,0007	0,3823	-0,0015	**-0,0019**	**-0,0038**	0,0390	-0,0024
3	12286720	rs9850825	0,4597	0,4816	0,4862	0,0046	0,4908	0,0092	**0,0094**	**0,0186**	0,0454	0,0177
3	12302462	rs9878908	0,2157	0,2478	0,2532	0,0054	0,2587	0,0108	**0,0213**	**0,0418**	0,1296	0,0144
3	12393125	rs1801282	0,1586	0,1698	0,1708	0,0010	0,1718	0,0021	**0,0060**	**0,0119**	0,0657	0,0025
3	12402474	rs1373641	0,4434	0,4265	0,4315	0,0050	0,4365	0,0100	**0,0116**	**0,0228**	-0,0396	0,0174
3	12475088	rs7626560	0,2525	0,2660	0,2661	0,0002	0,2663	0,0003	**0,0007**	**0,0013**	0,0504	0,0005
3	151007310	rs9863983	0,2863	0,3365	0,3392	0,0027	0,3419	0,0054	**0,0080**	**0,0159**	0,1492	0,0082
3	151041513	rs3971191	0,2317	0,2522	0,2616	0,0094	0,2711	0,0189	**0,0361**	**0,0697**	0,0813	0,0252
3	151053898	rs7644001	0,4390	0,4656	0,4693	0,0037	0,4731	0,0075	**0,0080**	**0,0159**	0,0571	0,0140
3	151090963	rs9859538	0,4873	0,4986	0,5010	0,0024	0,5034	0,0048	**0,0048**	**0,0095**	0,0228	0,0096
3	151112568	rs3732768	0,3420	0,3461	0,3454	-0,0007	0,3447	-0,0014	**-0,0021**	**-0,0042**	0,0118	-0,0022
3	151128895	rs10935844	0,5030	0,4965	0,5008	0,0043	0,5050	0,0086	**0,0085**	**0,0169**	-0,0131	0,0170
3	151147968	rs6772196	0,1987	0,2144	0,2143	0,0000	0,2143	-0,0001	**-0,0002**	**-0,0004**	0,0730	-0,0001
5	51405600	rs12655411	0,4656	0,4594	0,4641	0,0047	0,4688	0,0093	**0,0101**	**0,0199**	-0,0134	0,0173
5	51431680	rs10064799	0,4520	0,4552	0,4752	0,0200	0,4952	0,0401	**0,0422**	**0,0809**	0,0070	0,0736
5	51505665	rs6865397	0,4850	0,4959	0,4967	0,0007	0,4974	0,0015	**0,0015**	**0,0030**	0,0220	0,0029
5	51572584	rs4572960	0,4815	0,4803	0,4803	0,0000	NA	NA	**0,0000**	**NaN**	-0,0025	NA
5	74616843	rs10474433	0,5285	0,4732	0,4744	0,0012	0,4756	0,0025	**0,0026**	**0,0052**	-0,1168	0,0047
5	74620912	rs6878576	0,2164	0,2273	0,2269	-0,0004	0,2265	-0,0008	**-0,0018**	**-0,0035**	0,0479	-0,0010
5	74625487	rs7703051	0,4762	0,4556	0,4597	0,0041	0,4638	0,0081	**0,0088**	**0,0175**	-0,0452	0,0149
5	74648603	rs12654264	0,4795	0,4569	0,4599	0,0030	0,4628	0,0059	**0,0064**	**0,0128**	-0,0494	0,0109
5	74651084	rs3846662	0,5188	0,4994	0,4988	-0,0006	0,4981	-0,0013	**-0,0013**	**-0,0026**	-0,0388	-0,0026
5	74655726	rs3846663	0,4705	0,4560	0,4592	0,0032	0,4624	0,0063	**0,0069**	**0,0137**	-0,0317	0,0116
5	74656175	rs5909	0,1371	0,1412	0,1409	-0,0003	0,1405	-0,0007	**-0,0024**	**-0,0048**	0,0293	-0,0008
6	160496055	rs3777406	0,2036	0,2425	0,2425	0,0000	0,2426	0,0001	**0,0002**	**0,0003**	0,1602	0,0001
6	160517481	rs1803989	0,1482	0,1432	0,1439	0,0007	0,1446	0,0015	**0,0051**	**0,0101**	-0,0354	0,0017
6	160528057	rs7753051	0,2974	0,3390	0,3398	0,0008	0,3406	0,0015	**0,0022**	**0,0045**	0,1228	0,0023
6	160572866	rs622342	0,4004	0,4060	0,4137	0,0077	0,4215	0,0155	**0,0187**	**0,0368**	0,0138	0,0261
6	160578860	rs1564348	0,2982	0,2913	0,2907	-0,0006	0,2901	-0,0012	**-0,0021**	**-0,0042**	-0,0238	-0,0017
6	160581374	rs651164	0,4644	0,4319	0,4427	0,0108	0,4535	0,0216	**0,0244**	**0,0477**	-0,0754	0,0381
6	160582340	rs9456505	0,1796	0,1872	0,1877	0,0005	0,1882	0,0010	**0,0027**	**0,0053**	0,0405	0,0012
6	160635886	rs10945656	0,2307	0,2540	0,2538	-0,0001	0,2537	-0,0002	**-0,0005**	**-0,0010**	0,0914	-0,0003
6	160637239	rs596881	0,2363	0,2773	0,2768	-0,0005	0,2763	-0,0010	**-0,0017**	**-0,0035**	0,1479	-0,0013
6	160672625	rs316013	0,3758	0,4375	0,4368	-0,0007	0,4360	-0,0015	**-0,0017**	**-0,0033**	0,1411	-0,0026
6	160681393	rs3127573	0,2056	0,2295	0,2310	0,0014	0,2324	0,0029	**0,0063**	**0,0124**	0,1044	0,0038
6	160682897	rs7757997	0,1772	0,1709	0,1739	0,0030	0,1769	0,0060	**0,0172**	**0,0338**	-0,0364	0,0072
6	160687866	rs316030	0,4269	0,4160	0,4158	-0,0002	0,4156	-0,0004	**-0,0005**	**-0,0010**	-0,0261	-0,0007
6	160699534	rs2619276	0,3882	0,3461	0,3462	0,0001	0,3462	0,0001	**0,0002**	**0,0004**	-0,1216	0,0002
7	87103670	rs2888611	0,3553	0,3315	0,3382	0,0068	0,3450	0,0135	**0,0200**	**0,0392**	-0,0720	0,0202
7	87154646	rs10225473	0,2665	0,2568	0,2563	-0,0004	0,2559	-0,0009	**-0,0017**	**-0,0033**	-0,0378	-0,0012
7	87163016	rs11760837	0,2393	0,2374	0,2378	0,0004	0,2382	0,0008	**0,0016**	**0,0032**	-0,0078	0,0010
7	87179143	rs2235033	0,5229	0,5006	0,4995	-0,0011	0,4984	-0,0022	**-0,0022**	**-0,0044**	-0,0446	-0,0044
7	87179809	rs2229109	0,0736	0,0781	0,0779	-0,0002	0,0777	-0,0003	**-0,0021**	**-0,0043**	0,0574	-0,0004
7	87180198	rs10276036	0,5010	0,4842	0,4836	-0,0005	0,4831	-0,0011	**-0,0011**	**-0,0022**	-0,0348	-0,0021
7	87183354	rs1922240	0,4396	0,4422	0,4416	-0,0006	0,4410	-0,0012	**-0,0014**	**-0,0028**	0,0060	-0,0022
7	87201482	rs10260862	0,3301	0,3382	0,3379	-0,0003	0,3377	-0,0005	**-0,0008**	**-0,0016**	0,0239	-0,0008
7	87278760	rs10267099	0,4323	0,4041	0,4047	0,0006	0,4053	0,0012	**0,0015**	**0,0029**	-0,0697	0,0020
7	99207876	rs7792939	0,2010	0,2195	0,2267	0,0072	0,2340	0,0144	**0,0318**	**0,0617**	0,0844	0,0185
10	96581094	rs10786172	0,3902	0,3906	0,3905	0,0000	0,3905	-0,0001	**-0,0001**	**-0,0002**	0,0011	-0,0002
10	114711983	rs7094463	0,5560	0,5002	0,5006	0,0004	0,5009	0,0007	**0,0007**	**0,0014**	-0,1115	0,0014
10	114732906	rs7901275	0,4713	0,4877	0,4998	0,0121	0,5119	0,0242	**0,0242**	**0,0472**	0,0337	0,0472
10	114754088	rs7901695	0,4198	0,4413	0,4444	0,0032	0,4476	0,0063	**0,0071**	**0,0142**	0,0487	0,0114
10	114756041	rs4506565	0,4136	0,4418	0,4452	0,0033	0,4485	0,0067	**0,0075**	**0,0148**	0,0640	0,0119
10	114767771	rs4132670	0,4106	0,4522	0,4622	0,0100	0,4722	0,0200	**0,0217**	**0,0424**	0,0919	0,0365
10	114788815	rs12243326	0,3999	0,4259	0,4292	0,0033	0,4325	0,0066	**0,0077**	**0,0153**	0,0609	0,0116
10	114821249	rs11196212	0,4984	0,4787	0,5010	0,0223	0,5232	0,0446	**0,0445**	**0,0852**	-0,0413	0,0855
10	114855397	rs11196224	0,4222	0,4114	0,4410	0,0296	0,4706	0,0592	**0,0671**	**0,1258**	-0,0264	0,1005

Supplementary Table 2 next

Chr	Localisation	rs ID	Ho	Hs	Ht	Dst	Htp	Dstp	Fst	Fstp	Fis	Dest
10	114859463	rs7085532	0,4202	0,4412	0,4438	0,0026	0,4464	0,0052	**0,0059**	**0,0117**	0,0476	0,0093
10	114898093	rs3814573	0,4690	0,4789	0,4777	-0,0012	0,4765	-0,0024	**-0,0025**	**-0,0050**	0,0207	-0,0046
10	114912534	rs1555485	0,2222	0,2075	0,2208	0,0132	0,2340	0,0265	**0,0600**	**0,1131**	-0,0709	0,0334
11	2528003	rs11023096	0,3139	0,3074	0,3067	-0,0007	0,3060	-0,0014	**-0,0022**	**-0,0045**	-0,0211	-0,0020
11	2528233	rs4929992	0,5220	0,4920	0,5006	0,0087	0,5093	0,0173	**0,0173**	**0,0340**	-0,0610	0,0341
11	2550730	rs179429	0,2752	0,2819	0,2816	-0,0004	0,2812	-0,0007	**-0,0013**	**-0,0026**	0,0238	-0,0010
11	2553703	rs179435	0,3532	0,3731	0,3755	0,0024	0,3779	0,0048	**0,0064**	**0,0126**	0,0532	0,0076
11	2595287	rs2283171	0,4106	0,4290	0,4320	0,0030	0,4350	0,0060	**0,0070**	**0,0139**	0,0429	0,0106
11	2617782	rs1116714	0,3054	0,3239	0,3421	0,0182	0,3603	0,0364	**0,0532**	**0,1010**	0,0572	0,0538
11	2633152	rs10766212	0,4437	0,4862	0,4950	0,0088	0,5038	0,0175	**0,0177**	**0,0348**	0,0876	0,0341
11	2635797	rs2106467	0,4530	0,4719	0,4712	-0,0007	0,4705	-0,0014	**-0,0014**	**-0,0029**	0,0400	-0,0026
11	2673575	rs6578283	0,4401	0,4659	0,4656	-0,0003	0,4653	-0,0006	**-0,0006**	**-0,0013**	0,0553	-0,0011
11	2750703	rs170786	0,4678	0,4410	0,4443	0,0033	0,4476	0,0066	**0,0074**	**0,0147**	-0,0607	0,0118
11	2776448	rs11023996	0,1759	0,1787	0,1803	0,0016	0,1819	0,0032	**0,0089**	**0,0177**	0,0159	0,0039
11	2782648	rs548566	0,2619	0,3057	0,3058	0,0001	0,3059	0,0001	**0,0002**	**0,0004**	0,1434	0,0002
11	2821065	rs163171	0,3400	0,3383	0,3386	0,0003	0,3388	0,0005	**0,0007**	**0,0015**	-0,0051	0,0008
11	2837625	rs233446	0,3545	0,3572	0,3605	0,0032	0,3637	0,0065	**0,0090**	**0,0178**	0,0078	0,0101
11	2850782	rs234852	0,4092	0,4385	0,4437	0,0051	0,4488	0,0103	**0,0116**	**0,0229**	0,0669	0,0183
11	2895800	rs3987740	0,4089	0,4074	0,4138	0,0064	0,4202	0,0128	**0,0155**	**0,0304**	-0,0036	0,0216
11	17393644	rs12791318	0,3063	0,3257	0,3300	0,0043	0,3343	0,0086	**0,0130**	**0,0256**	0,0596	0,0127
11	17405333	rs10832785	0,4552	0,4969	0,4968	-0,0001	0,4966	-0,0003	**-0,0003**	**-0,0005**	0,0839	-0,0005
11	17408025	rs2285676	0,4806	0,4978	0,4971	-0,0007	0,4964	-0,0014	**-0,0014**	**-0,0029**	0,0347	-0,0029
11	17408630	rs5215	0,2925	0,3202	0,3263	0,0061	0,3325	0,0122	**0,0188**	**0,0368**	0,0866	0,0180
11	17408831	rs1800467	0,0384	0,0378	0,0378	-0,0001	0,0377	-0,0002	**-0,0020**	**-0,0041**	-0,0150	-0,0002
11	17438890	rs2074315	0,3081	0,3491	0,3503	0,0012	0,3515	0,0024	**0,0035**	**0,0069**	0,1173	0,0037
11	17441828	rs4757517	0,3991	0,4173	0,4174	0,0001	0,4175	0,0001	**0,0002**	**0,0003**	0,0437	0,0002
11	17496516	rs1048099	0,4727	0,4895	0,4983	0,0088	0,5072	0,0176	**0,0177**	**0,0348**	0,0343	0,0346
11	17510419	rs11603988	0,2375	0,2415	0,2502	0,0087	0,2589	0,0174	**0,0347**	**0,0671**	0,0169	0,0229
11	17510565	rs4757527	0,2877	0,3466	0,3463	-0,0002	0,3461	-0,0005	**-0,0007**	**-0,0014**	0,1697	-0,0007
11	17530484	rs7104083	0,4501	0,4924	0,5005	0,0081	0,5086	0,0162	**0,0162**	**0,0318**	0,0860	0,0319
11	17532597	rs1076311	0,4529	0,4907	0,5001	0,0094	0,5095	0,0188	**0,0188**	**0,0369**	0,0771	0,0369
11	17542649	rs2041032	0,4523	0,4957	0,4949	-0,0008	0,4941	-0,0016	**-0,0016**	**-0,0032**	0,0876	-0,0032
11	108097333	rs228591	0,4670	0,4752	0,4750	-0,0002	0,4747	-0,0005	**-0,0005**	**-0,0010**	0,0172	-0,0009
11	108268286	rs7931930	0,4908	0,4920	0,4920	0,0000	0,4920	0,0000	**0,0000**	**0,0001**	0,0023	0,0001
11	108283161	rs11212617	0,4620	0,4792	0,4784	-0,0008	0,4776	-0,0016	**-0,0017**	**-0,0034**	0,0357	-0,0031
16	31102321	rs7294	0,4075	0,4161	0,4349	0,0188	0,4536	0,0375	**0,0432**	**0,0827**	0,0206	0,0643
17	19447016	rs2440155	0,3296	0,3321	0,3314	-0,0007	0,3307	-0,0014	**-0,0022**	**-0,0043**	0,0076	-0,0021
17	19459537	rs2244280	0,2743	0,3245	0,3238	-0,0007	0,3231	-0,0014	**-0,0021**	**-0,0042**	0,1546	-0,0020
17	19484951	rs2453594	0,2788	0,3290	0,3281	-0,0009	0,3271	-0,0018	**-0,0028**	**-0,0056**	0,1526	-0,0027
17	19622643	rs11656096	0,2904	0,3185	0,3406	0,0222	0,3628	0,0443	**0,0651**	**0,1222**	0,0881	0,0650
17	19642952	rs2228100	0,3962	0,4315	0,4329	0,0014	0,4342	0,0027	**0,0031**	**0,0063**	0,0818	0,0048
17	19645938	rs887241	0,4451	0,4799	0,4866	0,0067	0,4933	0,0134	**0,0138**	**0,0273**	0,0725	0,0258
22	42152988	rs17377643	0,4511	0,4622	0,4686	0,0065	0,4751	0,0129	**0,0138**	**0,0272**	0,0240	0,0241
22	42178441	rs126092	0,2896	0,3833	0,3863	0,0030	0,3892	0,0059	**0,0076**	**0,0152**	0,2444	0,0096
22	46235677	rs1023470	0,3346	0,3508	0,3550	0,0042	0,3592	0,0084	**0,0118**	**0,0233**	0,0461	0,0129
22	46238069	rs8141212	0,4348	0,4654	0,4645	-0,0010	0,4635	-0,0020	**-0,0021**	**-0,0042**	0,0658	-0,0037
22	46525794	rs6007919	0,3613	0,4578	0,4567	-0,0010	0,4557	-0,0021	**-0,0023**	**-0,0045**	0,2107	-0,0038
22	46629479	rs4253776	0,1753	0,2063	0,2103	0,0040	0,2143	0,0080	**0,0190**	**0,0372**	0,1505	0,0101
22	46637254	rs9626814	0,1532	0,2000	0,2031	0,0031	0,2062	0,0062	**0,0152**	**0,0300**	0,2342	0,0077
22	46643774	rs16995069	0,1209	0,1365	0,1365	0,0000	0,1365	0,0000	**-0,0001**	**-0,0002**	0,1143	0,0000
22	46670394	rs6007761	0,1777	0,2323	0,2328	0,0005	0,2333	0,0009	**0,0020**	**0,0040**	0,2353	0,0012

$overall	Ho	Hs	Ht	Dst	Htp	Dstp	Fst	Fstp	Fis	Dest
	0,3547	0,3681	0,3714	0,0033	0,3738	0,0066	**0,0088**	**0,0177**	0,0366	0,0105

**$FST**	0,0175
**$FIS**	0,0447

Supplementary Table 2 next

Fst Comparison among Tunisian and Finish populations

Chr	Localisation	rs ID	Ho	Hs	Ht	Dst	Htp	Dstp	Fst	Fstp	Fis	Dest
1	65381861	rs12563017	0,1447	0,1892	0,1887	-0,0005	0,1883	-0,0009	**-0,0024**	**-0,0048**	0,2353	-0,0011
1	65389835	rs10889503	0,3876		0,4164	-0,0008	0,4156	-0,0017	**-0,0020**	**-0,0040**	0,0710	-0,0029
1	65421058	rs4916014	0,3782	0,4456	0,4448	-0,0007	0,4441	-0,0015	**-0,0016**	**-0,0033**	0,1513	-0,0026
1	65427476	rs4915675		0,4111	0,4152	0,0041	0,4194	0,0083	**0,0099**	**0,0197**	0,0628	0,0140
1	65516055	rs6588109	0,3721	0,4166	0,4157	-0,0010	0,4147	-0,0019	**-0,0023**	**-0,0047**	0,1070	-0,0033
1	65557876	rs6699671	0,2109	0,2029	0,2067	0,0038	0,2106	0,0076	**0,0185**	**0,0363**	-0,0391	0,0096
1	65583858	rs11208591	0,5337	0,4941	0,4940	-0,0001	0,4940	-0,0001	**-0,0001**	**-0,0002**	-0,0802	-0,0002
1	65619880	rs10789171	0,3387	0,3662	0,3673	0,0011	0,3683	0,0021	**0,0029**	**0,0058**	0,0750	0,0033
1	65658412	rs6677316	0,3904	0,4089	0,4086	-0,0003	0,4083	-0,0006	**-0,0007**	**-0,0015**	0,0452	-0,0010
3	12286720	rs9850825	0,4659	0,4665	0,4655	-0,0009	0,4646	-0,0019	**-0,0020**	**-0,0040**	0,0012	-0,0035
3	12302462	rs9878908	0,2551	0,2820	0,2965	0,0145	0,3110	0,0291	**0,0490**	**0,0934**	0,0952	0,0405
3	12393125	rs1801282	0,2365	0,2155	0,2222	0,0067	0,2289	0,0135	**0,0303**	**0,0589**	-0,0975	0,0172
3	12402474	rs1373641	0,3973	0,4195	0,4220	0,0026	0,4246	0,0052	**0,0061**	**0,0121**	0,0528	0,0089
3	12475088	rs7626560	0,2643	0,2499	0,2513	0,0014	0,2526	0,0028	**0,0055**	**0,0109**	-0,0578	0,0037
3	151007310	rs9863983	0,3188	0,3384	0,3416	0,0032	0,3448	0,0064	**0,0094**	**0,0186**	0,0577	0,0097
3	151041513	rs3971191	0,2197	0,2162	0,2192	0,0030	0,2222	0,0060	**0,0137**	**0,0270**	-0,0161	0,0077
3	151053898	rs7644001	0,4410	0,4900	0,4888	-0,0012	0,4876	-0,0024	**-0,0025**	**-0,0050**	0,1001	-0,0048
3	151090963	rs9859538	0,4622	0,4904	0,5002	0,0098	0,5100	0,0195	**0,0195**	**0,0383**	0,0575	0,0384
3	151112568	rs3732768	0,2755	0,3107	0,3107	-0,0001	0,3106	-0,0001	**-0,0002**	**-0,0004**	0,1132	-0,0002
3	151128895	rs10935844	0,4729	0,4943	0,4941	-0,0002	0,4938	-0,0005	**-0,0005**	**-0,0009**	0,0433	-0,0009
3	151147968	rs6772196	0,1843	0,1945	0,1940	-0,0004	0,1936	-0,0009	**-0,0023**	**-0,0045**	0,0521	-0,0011
5	51405600	rs12655411	0,4975	0,4629	0,4709	0,0080	0,4790	0,0160	**0,0170**	**0,0334**	-0,0746	0,0298
5	51431680	rs10064799	0,4269	0,4532	0,4864	0,0332	0,5197	0,0665	**0,0683**	**0,1279**	0,0579	0,1216
5	51505665	rs6865397	0,5000	0,4951	0,4999	0,0048	0,5048	0,0097	**0,0097**	**0,0192**	-0,0098	0,0192
5	51572584	rs4572960	0,4815	0,4803	0,4803	0,0000	NA	NA	**0,0000**	**NaN**	-0,0025	NA
5	74616843	rs10474433	0,4911	0,4753	0,4760	0,0008	0,4768	0,0015	**0,0016**	**0,0032**	-0,0333	0,0029
5	74620912	rs6878576	0,1874	0,1951	0,1964	0,0012	0,1976	0,0025	**0,0064**	**0,0126**	0,0398	0,0031
5	74625487	rs7703051	0,4136	0,4588	0,4647	0,0059	0,4706	0,0118	**0,0127**	**0,0250**	0,0985	0,0218
5	74648603	rs12654264	0,4114	0,4612	0,4663	0,0052	0,4715	0,0103	**0,0111**	**0,0219**	0,1080	0,0192
5	74651084	rs3846662	0,4654	0,4999	0,4991	-0,0008	0,4983	-0,0016	**-0,0016**	**-0,0033**	0,0691	-0,0033
5	74655726	rs3846663	0,4024	0,4603	0,4657	0,0054	0,4712	0,0108	**0,0116**	**0,0230**	0,1258	0,0201
5	74656175	rs5909	0,1662	0,1730	0,1734	0,0004	0,1739	0,0009	**0,0026**	**0,0051**	0,0392	0,0011
6	160496055	rs3777406	0,2356	0,2404	0,2404	0,0000	0,2405	0,0001	**0,0002**	**0,0004**	0,0198	0,0001
6	160517481	rs1803989	0,1539	0,1482	0,1486	0,0005	0,1491	0,0010	**0,0033**	**0,0066**	-0,0387	0,0012
6	160528057	rs7753051	0,3414	0,3451	0,3454	0,0003	0,3457	0,0007	**0,0010**	**0,0020**	0,0105	0,0010
6	160572866	rs622342	0,4065	0,4128	0,4245	0,0117	0,4362	0,0233	**0,0275**	**0,0534**	0,0153	0,0397
6	160578860	rs1564348	0,2702	0,2713	0,2709	-0,0004	0,2704	-0,0009	**-0,0016**	**-0,0032**	0,0041	-0,0012
6	160581374	rs651164	0,4829	0,4522	0,4571	0,0049	0,4620	0,0098	**0,0107**	**0,0212**	-0,0679	0,0179
6	160582340	rs9456505	0,1408	0,1466	0,1462	-0,0003	0,1459	-0,0006	**-0,0021**	**-0,0043**	0,0390	-0,0007
6	160635886	rs10945656	0,2008	0,2067	0,2068	0,0000	0,2068	0,0001	**0,0001**	**0,0003**	0,0290	0,0001
6	160637239	rs596881	0,1760	0,2124	0,2175	0,0051	0,2226	0,0102	**0,0234**	**0,0457**	0,1716	0,0129
6	160672625	rs316013	0,3477	0,3562	0,3644	0,0082	0,3726	0,0164	**0,0225**	**0,0441**	0,0237	0,0255
6	160681393	rs3127573	0,1958	0,1902	0,1899	-0,0004	0,1895	-0,0007	**-0,0019**	**-0,0039**	-0,0291	-0,0009
6	160682897	rs7757997	0,1778	0,1714	0,1744	0,0030	0,1774	0,0059	**0,0170**	**0,0334**	-0,0369	0,0071
6	160687866	rs316030	0,4087	0,4429	0,4509	0,0080	0,4589	0,0160	**0,0178**	**0,0349**	0,0773	0,0288
6	160699534	rs2619276	0,3764	0,3589	0,3603	0,0014	0,3617	0,0028	**0,0038**	**0,0076**	-0,0486	0,0043
7	87103670	rs2888611	0,3682	0,3772	0,3775	0,0003	0,3779	0,0007	**0,0009**	**0,0018**	0,0240	0,0011
7	87154646	rs10225473	0,1484	0,1612	0,1659	0,0047	0,1707	0,0095	**0,0286**	**0,0556**	0,0793	0,0113
7	87163016	rs11760837	0,1212	0,1418	0,1443	0,0025	0,1468	0,0049	**0,0171**	**0,0336**	0,1454	0,0057
7	87179143	rs2235033	0,5108	0,4938	0,4995	0,0056	0,5051	0,0113	**0,0113**	**0,0223**	-0,0343	0,0222
7	87179809	rs2229109	0,0397	0,0459	0,0461	0,0002	0,0464	0,0005	**0,0054**	**0,0107**	0,1343	0,0005
7	87180198	rs10276036	0,5114	0,4850	0,4847	-0,0002	0,4845	-0,0005	**-0,0005**	**-0,0010**	-0,0544	-0,0010
7	87183354	rs1922240	0,4618	0,4509	0,4499	-0,0010	0,4490	-0,0019	**-0,0021**	**-0,0043**	-0,0241	-0,0035
7	87201482	rs10260862	0,3118	0,3182	0,3196	0,0014	0,3209	0,0027	**0,0042**	**0,0084**	0,0204	0,0040
7	87278760	rs10267099	0,3631	0,3580	0,3581	0,0001	0,3582	0,0002	**0,0003**	**0,0005**	-0,0142	0,0003
7	99207876	rs7792939	0,1890	0,1889	0,1916	0,0027	0,1943	0,0054	**0,0140**	**0,0277**	-0,0003	0,0066
10	96581094	rs10786172	0,3774	0,4220	0,4300	0,0080	0,4380	0,0160	**0,0186**	**0,0365**	0,1057	0,0277
10	114711983	rs7094463	0,4609	0,4990	0,5012	0,0022	0,5034	0,0044	**0,0044**	**0,0087**	0,0764	0,0087
10	114732906	rs7901275	0,4664	0,4832	0,5009	0,0177	0,5185	0,0353	**0,0353**	**0,0681**	0,0348	0,0683
10	114754088	rs7901695	0,3919	0,4308	0,4364	0,0056	0,4420	0,0112	**0,0128**	**0,0254**	0,0902	0,0197
10	114756041	rs4506565	0,3857	0,4313	0,4372	0,0058	0,4430	0,0116	**0,0133**	**0,0262**	0,1058	0,0204
10	114767771	rs4132670	0,3929	0,4464	0,4584	0,0121	0,4705	0,0242	**0,0263**	**0,0513**	0,1198	0,0436
10	114788815	rs12243326	0,3207	0,3988	0,4083	0,0095	0,4177	0,0189	**0,0232**	**0,0453**	0,1959	0,0315
10	114821249	rs11196212	0,4703	0,4884	0,5003	0,0119	0,5122	0,0238	**0,0238**	**0,0465**	0,0371	0,0465
10	114855397	rs11196224	0,4388	0,4357	0,4553	0,0196	0,4749	0,0393	**0,0431**	**0,0826**	-0,0071	0,0696

Fst_TUN_FIN

Chr	Localisation	rs ID	Ho	Hs	Ht	Dst	Htp	Dstp	Fst	Fstp	Fis	Dest
10	114859463	rs7085532	0,3712	0,4053	0,4044	-0,0009	0,4036	-0,0017	**-0,0021**	**-0,0042**	0,0841	-0,0029
10	114898093	rs3814573	0,4637	0,4724	0,4714	-0,0010	0,4704	-0,0019	**-0,0021**	**-0,0041**	0,0184	-0,0037
10	114912534	rs1555485	0,2173	0,2208	0,2381	0,0174	0,2555	0,0347	**0,0729**	**0,1359**	0,0160	0,0446
11	2528003	rs11023096	0,3350	0,3564	0,3594	0,0030	0,3624	0,0060	**0,0083**	**0,0165**	0,0599	0,0093
11	2528233	rs4929992	0,4631	0,4921	0,5007	0,0087	0,5094	0,0173	**0,0173**	**0,0340**	0,0589	0,0341
11	2550730	rs179429	0,2380	0,2548	0,2543	-0,0005	0,2538	-0,0010	**-0,0019**	**-0,0038**	0,0659	-0,0013
11	2553703	rs179435	0,3037	0,3467	0,3535	0,0068	0,3602	0,0135	**0,0191**	**0,0375**	0,1241	0,0207
11	2595287	rs2283171	0,3529	0,4022	0,4113	0,0091	0,4204	0,0182	**0,0221**	**0,0433**	0,1226	0,0305
11	2617782	rs1116714	0,2611	0,2787	0,3093	0,0306	0,3400	0,0613	**0,0990**	**0,1802**	0,0630	0,0849
11	2633152	rs10766212	0,4209	0,4525	0,4796	0,0271	0,5066	0,0541	**0,0564**	**0,1068**	0,0700	0,0988
11	2635797	rs2106467	0,3889	0,4160	0,4287	0,0127	0,4414	0,0254	**0,0296**	**0,0575**	0,0652	0,0435
11	2673575	rs6578283	0,4540	0,4351	0,4414	0,0062	0,4476	0,0125	**0,0141**	**0,0278**	-0,0434	0,0221
11	2750703	rs170786	0,4189	0,3979	0,4125	0,0146	0,4272	0,0292	**0,0354**	**0,0685**	-0,0526	0,0486
11	2776448	rs11023996	0,2410	0,2558	0,2732	0,0174	0,2905	0,0347	**0,0635**	**0,1195**	0,0581	0,0466
11	2782648	rs548566	0,2655	0,2986	0,2983	-0,0003	0,2980	-0,0006	**-0,0010**	**-0,0019**	0,1107	-0,0008
11	2821065	rs163171	0,3254	0,3588	0,3580	-0,0008	0,3571	-0,0017	**-0,0023**	**-0,0046**	0,0931	-0,0026
11	2837625	rs233446	0,3431	0,3336	0,3338	0,0002	0,3340	0,0004	**0,0005**	**0,0011**	-0,0283	0,0005
11	2850782	rs234852	0,3611	0,4120	0,4244	0,0124	0,4369	0,0249	**0,0293**	**0,0570**	0,1235	0,0423
11	2895800	rs3987740	0,3727	0,3911	0,4014	0,0103	0,4117	0,0207	**0,0257**	**0,0502**	0,0469	0,0339
11	17393644	rs12791318	0,2462	0,2551	0,2547	-0,0005	0,2542	-0,0009	**-0,0019**	**-0,0037**	0,0350	-0,0013
11	17405333	rs10832785	0,5022	0,4843	0,4890	0,0048	0,4938	0,0095	**0,0097**	**0,0192**	-0,0369	0,0184
11	17408025	rs2285676	0,5216	0,4899	0,4918	0,0019	0,4937	0,0038	**0,0039**	**0,0077**	-0,0648	0,0074
11	17408630	rs5215	0,3657	0,3730	0,4168	0,0438	0,4606	0,0876	**0,1050**	**0,1901**	0,0198	0,1397
11	17408831	rs1800467	0,1027	0,0961	0,0977	0,0015	0,0992	0,0030	**0,0156**	**0,0307**	-0,0685	0,0034
11	17438890	rs2074315	0,3499	0,3645	0,3687	0,0041	0,3728	0,0083	**0,0113**	**0,0223**	0,0401	0,0131
11	17441828	rs4757517	0,4277	0,4205	0,4212	0,0007	0,4218	0,0013	**0,0016**	**0,0031**	-0,0170	0,0023
11	17496516	rs1048099	0,5193	0,4810	0,5009	0,0199	0,5208	0,0398	**0,0398**	**0,0765**	-0,0796	0,0768
11	17510419	rs11603988	0,2397	0,2460	0,2558	0,0098	0,2656	0,0196	**0,0384**	**0,0739**	0,0254	0,0260
11	17510565	rs4757527	0,2918	0,3197	0,3221	0,0024	0,3244	0,0047	**0,0073**	**0,0145**	0,0874	0,0069
11	17530484	rs7104083	0,4609	0,4760	0,4999	0,0239	0,5238	0,0478	**0,0478**	**0,0912**	0,0318	0,0912
11	17532597	rs1076311	0,4788	0,4756	0,5004	0,0249	0,5253	0,0497	**0,0497**	**0,0947**	-0,0068	0,0948
11	17542649	rs2041032	0,4883	0,4980	0,4969	-0,0011	0,4958	-0,0022	**-0,0022**	**-0,0043**	0,0194	-0,0043
11	108097333	rs228591	0,4264	0,4766	0,4767	0,0001	0,4768	0,0001	**0,0001**	**0,0003**	0,1054	0,0002
11	108268286	rs7931930	0,4553	0,4942	0,4937	-0,0006	0,4931	-0,0012	**-0,0012**	**-0,0024**	0,0789	-0,0023
11	108283161	rs11212617	0,4265	0,4814	0,4810	-0,0004	0,4806	-0,0008	**-0,0009**	**-0,0018**	0,1141	-0,0016
16	31102321	rs7294	0,3449	0,4157	0,4337	0,0180	0,4517	0,0360	**0,0415**	**0,0797**	0,1702	0,0616
17	19447016	rs2440155	0,3108	0,3123	0,3119	-0,0004	0,3114	-0,0009	**-0,0014**	**-0,0029**	0,0050	-0,0013
17	19459537	rs2244280	0,2674	0,3178	0,3175	-0,0004	0,3171	-0,0007	**-0,0011**	**-0,0022**	0,1586	-0,0010
17	19484951	rs2453594	0,2516	0,2881	0,2896	0,0015	0,2911	0,0030	**0,0052**	**0,0103**	0,1265	0,0042
17	19622643	rs11656096	0,3144	0,3370	0,3752	0,0381	0,4133	0,0763	**0,1017**	**0,1845**	0,0672	0,1150
17	19642952	rs2228100	0,3596	0,3749	0,3891	0,0142	0,4033	0,0284	**0,0365**	**0,0704**	0,0408	0,0454
17	19645938	rs887241	0,4466	0,4906	0,4929	0,0023	0,4952	0,0046	**0,0047**	**0,0093**	0,0897	0,0091
22	42152988	rs17377643	0,4320	0,4615	0,4674	0,0059	0,4733	0,0118	**0,0126**	**0,0250**	0,0639	0,0219
22	42178441	rs126092	0,3097	0,3848	0,3875	0,0028	0,3903	0,0056	**0,0072**	**0,0143**	0,1950	0,0091
22	46235677	rs1023470	0,3164	0,3352	0,3367	0,0015	0,3382	0,0030	**0,0045**	**0,0089**	0,0560	0,0045
22	46238069	rs8141212	0,4997	0,4768	0,4789	0,0021	0,4810	0,0042	**0,0044**	**0,0088**	-0,0479	0,0081
22	46525794	rs6007919	0,4027	0,4617	0,4613	-0,0004	0,4609	-0,0008	**-0,0008**	**-0,0016**	0,1278	-0,0014
22	46629479	rs4253776	0,2158	0,2388	0,2398	0,0010	0,2407	0,0019	**0,0040**	**0,0081**	0,0962	0,0025
22	46637254	rs9626814	0,1837	0,2245	0,2255	0,0010	0,2265	0,0020	**0,0043**	**0,0086**	0,1821	0,0025
22	46643774	rs16995069	0,1118	0,1276	0,1279	0,0003	0,1282	0,0007	**0,0026**	**0,0052**	0,1234	0,0008
22	46670394	rs6007761	0,1807	0,2410	0,2410	0,0000	0,2410	0,0000	**-0,0001**	**-0,0002**	0,2505	-0,0001

	Ho	Hs	Ht	Dst	Htp	Dstp	Fst	Fstp	Fis	Dest
**$overall**	0,3454	0,3625	0,3681	0,0056	0,3728	0,0114	**0,0153**	**0,0305**	**0,0470**	0,0179

$FST 0,0302

$FIS 0,0516

Supplementary Table 2 next

Fst Comparison among Tunisian and Yoruba populations

Chr	Localisation	rs ID	Ho	Hs	Ht	Dst	Htp	Dstp	Fst	Fstp	Fis	Dest
1	65381861	rs12563017	0,1594	0,1927	0,1923	-0,0003	0,1920	-0,0007	**-0,0018**	**-0,0036**	0,1728	-0,0009
1	65389835	rs10889503	0,2626		0,4907	0,1621	0,6528	0,3241	**0,3303**	**0,4965**	0,2009	0,4828
1	65421058	rs4916014	0,2241	0,3093	0,4781	0,1688	0,6469	0,3376	**0,3530**	**0,5218**	0,2755	0,4887
1	65427476	rs4915675		0,3302	0,4986	0,1683	0,6669	0,3366	**0,3376**	**0,5048**	0,1161	0,5026
1	65516055	rs6588109	0,4352	0,4553	0,4874	0,0321	0,5195	0,0643	**0,0659**	**0,1237**	0,0441	0,1180
1	65557876	rs6699671	0,3485	0,3553	0,3668	0,0115	0,3783	0,0230	**0,0313**	**0,0608**	0,0191	0,0357
1	65583858	rs11208591	0,2993	0,2722	0,4039	0,1317	0,5356	0,2633	**0,3260**	**0,4917**	-0,0993	0,3619
1	65619880	rs10789171	0,3690	0,4173	0,4506	0,0333	0,4839	0,0666	**0,0739**	**0,1376**	0,1157	0,1143
1	65658412	rs6677316	0,4809	0,4465	0,4657	0,0192	0,4849	0,0384	**0,0413**	**0,0792**	-0,0770	0,0694
3	12286720	rs9850825	0,5105	0,4718	0,5002	0,0284	0,5285	0,0567	**0,0567**	**0,1074**	-0,0821	0,1074
3	12302462	rs9878908	0,1663	0,1655	0,1652	-0,0003	0,1648	-0,0007	**-0,0020**	**-0,0040**	-0,0050	-0,0008
3	12393125	rs1801282	0,0597	0,0629	0,0650	0,0021	0,0672	0,0042	**0,0325**	**0,0630**	0,0513	0,0045
3	12402474	rs1373641	0,3102	0,3163	0,3223	0,0060	0,3284	0,0121	**0,0187**	**0,0367**	0,0194	0,0176
3	12475088	rs7626560	0,3426	0,3350	0,3370	0,0019	0,3389	0,0039	**0,0057**	**0,0114**	-0,0226	0,0058
3	151007310	rs9863983	0,3816	0,3938	0,4363	0,0425	0,4788	0,0850	**0,0974**	**0,1776**	0,0311	0,1402
3	151041513	rs3971191	0,2315	0,2282	0,2329	0,0047	0,2377	0,0094	**0,0202**	**0,0397**	-0,0143	0,0122
3	151053898	rs7644001	0,4683	0,4959	0,4962	0,0003	0,4965	0,0006	**0,0006**	**0,0012**	0,0555	0,0012
3	151090963	rs9859538	0,2956	0,3011	0,3774	0,0763	0,4538	0,1527	**0,2023**	**0,3365**	0,0184	0,2185
3	151112568	rs3732768	0,4174	0,4052	0,4192	0,0140	0,4332	0,0280	**0,0334**	**0,0646**	-0,0299	0,0470
3	151128895	rs10935844	0,5474	0,5004	0,5000	-0,0004	0,4996	-0,0008	**-0,0008**	**-0,0016**	-0,0938	-0,0016
3	151147968	rs6772196	0,2454	0,2438	0,2461	0,0023	0,2483	0,0045	**0,0092**	**0,0182**	-0,0065	0,0060
5	51405600	rs12655411	0,3598	0,3913	0,3939	0,0026	0,3965	0,0052	**0,0066**	**0,0132**	0,0804	0,0086
5	51431680	rs10064799	0,3668	0,4035	0,4026	-0,0009	0,4018	-0,0018	**-0,0022**	**-0,0044**	0,0911	-0,0030
5	51505665	rs6865397	0,4036	0,4159	0,4802	0,0643	0,5445	0,1286	**0,1339**	**0,2362**	0,0296	0,2201
5	51572584	rs4572960	0,4815	0,4803	0,4803	0,0000	NA	NA	**0,0000**	**NaN**	-0,0025	NA
5	74616843	rs10474433	0,4991	0,4796	0,4795	0,0000	0,4795	-0,0001	**-0,0001**	**-0,0002**	-0,0408	-0,0002
5	74620912	rs6878576	0,2732	0,2889	0,2909	0,0020	0,2930	0,0041	**0,0070**	**0,0140**	0,0541	0,0058
5	74625487	rs7703051	0,4237	0,4263	0,4254	-0,0009	0,4245	-0,0018	**-0,0021**	**-0,0042**	0,0062	-0,0031
5	74648603	rs12654264	0,4168	0,4231	0,4224	-0,0007	0,4217	-0,0014	**-0,0016**	**-0,0033**	0,0147	-0,0024
5	74651084	rs3846662	0,2747	0,2909	0,4040	0,1132	0,5172	0,2264	**0,2801**	**0,4376**	0,0554	0,3192
5	74655726	rs3846663	0,3246	0,3693	0,3756	0,0063	0,3819	0,0126	**0,0168**	**0,0331**	0,1212	0,0200
5	74656175	rs5909	0,0740	0,0926	0,0937	0,0011	0,0949	0,0022	**0,0120**	**0,0236**	0,2006	0,0025
6	160496055	rs3777406	0,2752	0,2591	0,2606	0,0015	0,2621	0,0029	**0,0057**	**0,0112**	-0,0618	0,0040
6	160517481	rs1803989	0,2877	0,2745	0,2837	0,0093	0,2930	0,0185	**0,0326**	**0,0632**	-0,0483	0,0255
6	160528057	rs7753051	0,4184	0,4295	0,4424	0,0129	0,4552	0,0257	**0,0291**	**0,0565**	0,0257	0,0451
6	160572866	rs622342	0,2938	0,3148	0,3158	0,0010	0,3168	0,0020	**0,0032**	**0,0063**	0,0668	0,0029
6	160578860	rs1564348	0,2454	0,2520	0,2525	0,0005	0,2530	0,0010	**0,0019**	**0,0038**	0,0263	0,0013
6	160581374	rs651164	0,4408	0,4207	0,4351	0,0144	0,4495	0,0288	**0,0331**	**0,0640**	-0,0477	0,0497
6	160582340	rs9456505	0,2094	0,2062	0,2082	0,0020	0,2102	0,0041	**0,0097**	**0,0193**	-0,0160	0,0051
6	160635886	rs10945656	0,2719	0,2640	0,2646	0,0006	0,2653	0,0012	**0,0023**	**0,0046**	-0,0297	0,0017
6	160637239	rs596881	0,3376	0,3594	0,3669	0,0075	0,3744	0,0149	**0,0203**	**0,0399**	0,0607	0,0233
6	160672625	rs316013	0,4572	0,4640	0,4784	0,0144	0,4928	0,0288	**0,0301**	**0,0585**	0,0147	0,0538
6	160681393	rs3127573	0,1773	0,1975	0,1972	-0,0003	0,1969	-0,0006	**-0,0014**	**-0,0028**	0,1023	-0,0007
6	160682897	rs7757997	0,2704	0,2582	0,2581	-0,0001	0,2580	-0,0001	**-0,0003**	**-0,0006**	-0,0473	-0,0002
6	160687866	rs316030	0,4146	0,3733	0,3739	0,0006	0,3746	0,0013	**0,0017**	**0,0034**	-0,1106	0,0020
6	160699534	rs2619276	0,3275	0,3135	0,3130	-0,0005	0,3126	-0,0010	**-0,0016**	**-0,0031**	-0,0447	-0,0014
7	87103670	rs2888611	0,4654	0,4500	0,4843	0,0343	0,5187	0,0687	**0,0709**	**0,1325**	-0,0343	0,1249
7	87154646	rs10225473	0,2848	0,2905	0,2924	0,0019	0,2943	0,0038	**0,0065**	**0,0129**	0,0198	0,0054
7	87163016	rs11760837	0,2761	0,2864	0,2936	0,0072	0,3007	0,0143	**0,0244**	**0,0477**	0,0360	0,0201
7	87179143	rs2235033	0,5074	0,5011	0,5008	-0,0003	0,5005	-0,0005	**-0,0005**	**-0,0011**	-0,0126	-0,0011
7	87179809	rs2229109	0,0296	0,0358	0,0364	0,0006	0,0370	0,0012	**0,0164**	**0,0323**	0,1733	0,0012
7	87180198	rs10276036	0,3973	0,3934	0,4126	0,0192	0,4319	0,0385	**0,0466**	**0,0891**	-0,0100	0,0635
7	87183354	rs1922240	0,3578	0,3805	0,3913	0,0108	0,4020	0,0215	**0,0275**	**0,0535**	0,0598	0,0347
7	87201482	rs10260862	0,3921	0,4282	0,4529	0,0247	0,4776	0,0495	**0,0546**	**0,1036**	0,0841	0,0865
7	87278760	rs10267099	0,3635	0,3557	0,3560	0,0003	0,3563	0,0006	**0,0009**	**0,0017**	-0,0219	0,0009
7	99207876	rs7792939	0,0526	0,0634	0,0655	0,0021	0,0677	0,0043	**0,0326**	**0,0631**	0,1699	0,0046
10	96581094	rs10786172	0,1889	0,2038	0,2288	0,0251	0,2539	0,0501	**0,1095**	**0,1974**	0,0731	0,0630
10	114711983	rs7094463	0,4668	0,4755	0,4812	0,0057	0,4869	0,0114	**0,0118**	**0,0233**	0,0185	0,0217
10	114732906	rs7901275	0,3953	0,4255	0,4352	0,0098	0,4450	0,0195	**0,0224**	**0,0439**	0,0709	0,0340
10	114754088	rs7901695	0,4731	0,4823	0,4827	0,0004	0,4830	0,0007	**0,0008**	**0,0015**	0,0189	0,0014
10	114756041	rs4506565	0,4669	0,4829	0,4831	0,0002	0,4833	0,0005	**0,0005**	**0,0009**	0,0330	0,0009
10	114767771	rs4132670	0,3992	0,4620	0,4933	0,0313	0,5246	0,0627	**0,0635**	**0,1194**	0,1359	0,1165
10	114788815	rs12243326	0,3960	0,4420	0,4426	0,0006	0,4431	0,0011	**0,0013**	**0,0025**	0,1040	0,0020
10	114821249	rs11196212	0,3145	0,3473	0,3854	0,0381	0,4235	0,0762	**0,0988**	**0,1799**	0,0944	0,1167
10	114855397	rs11196224	0,5065	0,4839	0,4857	0,0019	0,4876	0,0037	**0,0038**	**0,0077**	-0,0469	0,0072

Supplementary Table 2 next

Chr	Localisation	rs ID	Ho	Hs	Ht	Dst	Htp	Dstp	Fst	Fstp	Fis	Dest
10	114859463	rs7085532	0,3422	0,3422	0,3494	0,0072	0,3566	0,0144	**0,0206**	**0,0404**	0,0002	0,0219
10	114898093	rs3814573	0,3042	0,3191	0,3631	0,0441	0,4072	0,0881	**0,1213**	**0,2164**	0,0467	0,1294
10	114912534	rs1555485	0,0354	0,0416	0,0425	0,0008	0,0433	0,0017	**0,0197**	**0,0386**	0,1490	0,0017
11	2528003	rs11023096	0,3232	0,3230	0,3226	-0,0004	0,3222	-0,0008	**-0,0013**	**-0,0026**	-0,0006	-0,0012
11	2528233	rs4929992	0,3835	0,4041	0,4266	0,0225	0,4491	0,0450	**0,0527**	**0,1002**	0,0508	0,0755
11	2550730	rs179429	0,2796	0,2984	0,2991	0,0007	0,2998	0,0013	**0,0022**	**0,0045**	0,0629	0,0019
11	2553703	rs179435	0,3950	0,4401	0,4419	0,0018	0,4437	0,0036	**0,0041**	**0,0081**	0,1025	0,0064
11	2595287	rs2283171	0,3954	0,4123	0,4933	0,0810	0,5743	0,1619	**0,1641**	**0,2820**	0,0412	0,2756
11	2617782	rs1116714	0,4223	0,4447	0,5005	0,0558	0,5563	0,1116	**0,1115**	**0,2006**	0,0502	0,2010
11	2633152	rs10766212	0,4230	0,4622	0,4782	0,0159	0,4941	0,0318	**0,0333**	**0,0644**	0,0850	0,0592
11	2635797	rs2106467	0,4259	0,4527	0,4554	0,0028	0,4582	0,0056	**0,0061**	**0,0122**	0,0590	0,0102
11	2673575	rs6578283	0,4730	0,4832	0,5005	0,0173	0,5178	0,0346	**0,0346**	**0,0668**	0,0211	0,0669
11	2750703	rs170786	0,5009	0,4860	0,4890	0,0031	0,4921	0,0061	**0,0062**	**0,0124**	-0,0308	0,0119
11	2776448	rs11023996	0,1597	0,1665	0,1672	0,0008	0,1680	0,0015	**0,0046**	**0,0091**	0,0404	0,0018
11	2782648	rs548566	0,1746	0,2005	0,2052	0,0047	0,2099	0,0094	**0,0229**	**0,0448**	0,1291	0,0118
11	2821065	rs163171	0,4041	0,4297	0,4806	0,0509	0,5315	0,1018	**0,1059**	**0,1915**	0,0597	0,1785
11	2837625	rs233446	0,3363	0,3381	0,3387	0,0006	0,3392	0,0011	**0,0017**	**0,0034**	0,0051	0,0017
11	2850782	rs234852	0,4394	0,4824	0,5006	0,0183	0,5189	0,0366	**0,0365**	**0,0705**	0,0891	0,0707
11	2895800	rs3987740	0,4287	0,4403	0,4407	0,0004	0,4410	0,0007	**0,0008**	**0,0016**	0,0263	0,0013
11	17393644	rs12791318	0,3657	0,3648	0,3848	0,0200	0,4048	0,0400	**0,0519**	**0,0988**	-0,0025	0,0629
11	17405333	rs10832785	0,4744	0,4962	0,4998	0,0037	0,5035	0,0073	**0,0073**	**0,0146**	0,0439	0,0145
11	17408025	rs2285676	0,4795	0,4943	0,4996	0,0053	0,5050	0,0107	**0,0107**	**0,0212**	0,0298	0,0211
11	17408630	rs5215	0,0866	0,1389	0,1471	0,0082	0,1553	0,0164	**0,0559**	**0,1058**	0,3763	0,0191
11	17408831	rs1800467	0,0219	0,0215	0,0217	0,0002	0,0219	0,0004	**0,0089**	**0,0176**	-0,0179	0,0004
11	17438890	rs2074315	0,3508	0,4035	0,4716	0,0682	0,5398	0,1364	**0,1446**	**0,2526**	0,1306	0,2286
11	17441828	rs4757517	0,3662	0,3874	0,5006	0,1132	0,6138	0,2264	**0,2261**	**0,3689**	0,0547	0,3696
11	17496516	rs1048099	0,4401	0,4655	0,4998	0,0343	0,5342	0,0686	**0,0687**	**0,1285**	0,0545	0,1284
11	17510419	rs11603988	0,0874	0,0815	0,0837	0,0022	0,0859	0,0044	**0,0264**	**0,0514**	-0,0720	0,0048
11	17510565	rs4757527	0,3865	0,4330	0,4557	0,0227	0,4784	0,0454	**0,0498**	**0,0949**	0,1074	0,0801
11	17530484	rs7104083	0,4444	0,4547	0,4600	0,0053	0,4653	0,0106	**0,0115**	**0,0227**	0,0226	0,0194
11	17532597	rs1076311	0,3917	0,4180	0,4327	0,0147	0,4474	0,0294	**0,0340**	**0,0658**	0,0629	0,0506
11	17542649	rs2041032	0,4003	0,4998	0,5012	0,0015	0,5027	0,0029	**0,0029**	**0,0058**	0,1989	0,0059
11	108097333	rs228591	0,5013	0,4805	0,4978	0,0173	0,5150	0,0345	**0,0347**	**0,0670**	-0,0433	0,0664
11	108268286	rs7931930	0,4094	0,3979	0,4627	0,0647	0,5274	0,1294	**0,1399**	**0,2455**	-0,0288	0,2150
11	108283161	rs11212617	0,3806	0,3852	0,4810	0,0958	0,5768	0,1916	**0,1992**	**0,3322**	0,0119	0,3116
16	31102321	rs7294	0,3689	0,4227	0,4653	0,0425	0,5078	0,0851	**0,0914**	**0,1675**	0,1273	0,1474
17	19447016	rs2440155	0,3352	0,3365	0,3359	-0,0005	0,3354	-0,0011	**-0,0016**	**-0,0032**	0,0038	-0,0016
17	19459537	rs2244280	0,2510	0,2983	0,2994	0,0010	0,3004	0,0021	**0,0035**	**0,0070**	0,1587	0,0030
17	19484951	rs2453594	0,3480	0,3654	0,3666	0,0012	0,3678	0,0024	**0,0033**	**0,0066**	0,0475	0,0038
17	19622643	rs11656096	0,1242	0,1341	0,1364	0,0023	0,1387	0,0047	**0,0172**	**0,0338**	0,0738	0,0054
17	19642952	rs2228100	0,4661	0,4780	0,4828	0,0048	0,4875	0,0096	**0,0099**	**0,0196**	0,0249	0,0183
17	19645938	rs887241	0,4689	0,4882	0,4915	0,0033	0,4948	0,0066	**0,0067**	**0,0132**	0,0395	0,0128
22	42152988	rs17377643	0,2981	0,2919	0,3172	0,0253	0,3426	0,0507	**0,0799**	**0,1480**	-0,0214	0,0716
22	42178441	rs126092	0,4179	0,4384	0,5006	0,0622	0,5628	0,1244	**0,1243**	**0,2211**	0,0467	0,2216
22	46235677	rs1023470	0,2297	0,2683	0,2685	0,0002	0,2687	0,0004	**0,0007**	**0,0013**	0,1437	0,0005
22	46238069	rs8141212	0,4130	0,4184	0,4231	0,0047	0,4278	0,0094	**0,0111**	**0,0219**	0,0130	0,0161
22	46525794	rs6007919	0,3059	0,3384	0,4789	0,1405	0,6195	0,2811	**0,2934**	**0,4537**	0,0962	0,4248
22	46629479	rs4253776	0,3324	0,3902	0,4374	0,0472	0,4846	0,0943	**0,1079**	**0,1947**	0,1482	0,1547
22	46637254	rs9626814	0,3150	0,3823	0,4253	0,0430	0,4683	0,0859	**0,1010**	**0,1835**	0,1762	0,1391
22	46643774	rs16995069	0,3399	0,3023	0,3322	0,0299	0,3622	0,0598	**0,0900**	**0,1652**	-0,1245	0,0858
22	46670394	rs6007761	0,3397	0,3819	0,4600	0,0780	0,5380	0,1561	**0,1697**	**0,2901**	0,1104	0,2525

	Ho	Hs	Ht	Dst	Htp	Dstp	Fst	Fstp	Fis	Dest
**$overall**										
	0,3405	0,3542	0,3772	0,0229	0,3994	0,0463	**0,0608**	**0,1158**	0,0389	0,0716

$FST 0,1144

$FIS 0,0423

>
>

Supplementary Table 2 next

Fst Comparison among Tunisian and Luhya populations

Chr	Localisation	rs ID	Ho	Hs	Ht	Dst	Htp	Dstp	Fst	Fstp	Fis	Dest
1	65381861	rs12563017	0,1497	0,2008	0,2006	-0,0002	0,2004	-0,0004	**-0,0010**	**-0,0020**	0,2545	-0,0005
1	65389835	rs10889503	0,2967		0,4918	0,1575	0,6494	0,3151	**0,3203**	**0,4852**	0,1123	0,4733
1	65421058	rs4916014	0,2620	0,3320	0,4840	0,1520	0,6360	0,3040	**0,3140**	**0,4780**	0,2110	0,4551
1	65427476	rs4915675		0,3553	0,5005	0,1452	0,6456	0,2903	**0,2901**	**0,4497**	0,0151	0,4503
1	65516055	rs6588109	0,4731	0,4557	0,4693	0,0136	0,4829	0,0272	**0,0290**	**0,0564**	-0,0381	0,0500
1	65557876	rs6699671	0,2765	0,2625	0,2620	-0,0004	0,2616	-0,0008	**-0,0016**	**-0,0032**	-0,0536	-0,0011
1	65583858	rs11208591	0,3064	0,2791	0,4071	0,1280	0,5351	0,2560	**0,3144**	**0,4784**	-0,0979	0,3550
1	65619880	rs10789171	0,3943	0,4078	0,4246	0,0168	0,4415	0,0337	**0,0397**	**0,0763**	0,0331	0,0569
1	65658412	rs6677316	0,4864	0,4467	0,4663	0,0196	0,4859	0,0392	**0,0420**	**0,0807**	-0,0888	0,0709
3	12286720	rs9850825	0,4508	0,4776	0,4987	0,0211	0,5198	0,0422	**0,0423**	**0,0812**	0,0562	0,0808
3	12302462	rs9878908	0,0935	0,1097	0,1118	0,0020	0,1138	0,0041	**0,0182**	**0,0358**	0,1480	0,0046
3	12393125	rs1801282	0,0648	0,0680	0,0698	0,0018	0,0715	0,0035	**0,0254**	**0,0496**	0,0476	0,0038
3	12402474	rs1373641	0,3519	0,3518	0,3529	0,0012	0,3541	0,0023	**0,0033**	**0,0066**	-0,0003	0,0036
3	12475088	rs7626560	0,2643	0,2576	0,2584	0,0008	0,2591	0,0016	**0,0030**	**0,0060**	-0,0261	0,0021
3	151007310	rs9863983	0,3037	0,3581	0,3663	0,0082	0,3745	0,0164	**0,0224**	**0,0438**	0,1520	0,0255
3	151041513	rs3971191	0,1843	0,1837	0,1840	0,0003	0,1843	0,0006	**0,0017**	**0,0034**	-0,0034	0,0008
3	151053898	rs7644001	0,5117	0,4959	0,4964	0,0005	0,4969	0,0010	**0,0010**	**0,0020**	-0,0318	0,0020
3	151090963	rs9859538	0,3057	0,3101	0,3824	0,0724	0,4548	0,1448	**0,1893**	**0,3183**	0,0142	0,2098
3	151112568	rs3732768	0,3715	0,4140	0,4366	0,0226	0,4592	0,0452	**0,0517**	**0,0984**	0,1027	0,0771
3	151128895	rs10935844	0,5083	0,4976	0,5009	0,0033	0,5042	0,0066	**0,0066**	**0,0131**	-0,0214	0,0132
3	151147968	rs6772196	0,1944	0,2315	0,2324	0,0009	0,2334	0,0019	**0,0041**	**0,0081**	0,1600	0,0025
5	51405600	rs12655411	0,3561	0,3370	0,3506	0,0136	0,3642	0,0272	**0,0388**	**0,0747**	-0,0567	0,0411
5	51431680	rs10064799	0,3209	0,3631	0,3659	0,0028	0,3688	0,0057	**0,0077**	**0,0153**	0,1163	0,0089
5	51505665	rs6865397	0,3939	0,4242	0,4832	0,0590	0,5422	0,1180	**0,1221**	**0,2176**	0,0714	0,2049
5	51572584	rs4572960	0,4815	0,4803	0,4803	0,0000	NA	NA	**0,0000**	**NaN**	-0,0025	NA
5	74616843	rs10474433	0,5063	0,4941	0,4942	0,0000	0,4942	0,0001	**0,0001**	**0,0001**	-0,0245	0,0001
5	74620912	rs6878576	0,2833	0,3127	0,3189	0,0062	0,3251	0,0124	**0,0195**	**0,0382**	0,0940	0,0181
5	74625487	rs7703051	0,4591	0,4578	0,4632	0,0054	0,4686	0,0108	**0,0117**	**0,0231**	-0,0027	0,0199
5	74648603	rs12654264	0,4669	0,4584	0,4621	0,0037	0,4658	0,0074	**0,0080**	**0,0160**	-0,0186	0,0137
5	74651084	rs3846662	0,2634	0,2803	0,3990	0,1187	0,5177	0,2373	**0,2974**	**0,4584**	0,0605	0,3298
5	74655726	rs3846663	0,4226	0,4345	0,4336	-0,0009	0,4327	-0,0018	**-0,0020**	**-0,0041**	0,0273	-0,0031
5	74656175	rs5909	0,0955	0,1041	0,1047	0,0005	0,1052	0,0011	**0,0052**	**0,0104**	0,0829	0,0012
6	160496055	rs3777406	0,2962	0,2948	0,3020	0,0072	0,3092	0,0144	**0,0239**	**0,0466**	-0,0049	0,0204
6	160517481	rs1803989	0,2751	0,2646	0,2714	0,0068	0,2782	0,0136	**0,0251**	**0,0490**	-0,0399	0,0185
6	160528057	rs7753051	0,3869	0,4318	0,4466	0,0148	0,4614	0,0297	**0,0332**	**0,0643**	0,1040	0,0522
6	160572866	rs622342	0,3257	0,3372	0,3367	-0,0005	0,3362	-0,0010	**-0,0015**	**-0,0030**	0,0341	-0,0015
6	160578860	rs1564348	0,2298	0,2334	0,2353	0,0019	0,2372	0,0038	**0,0080**	**0,0159**	0,0154	0,0049
6	160581374	rs651164	0,4223	0,4130	0,4299	0,0169	0,4468	0,0339	**0,0394**	**0,0758**	-0,0226	0,0577
6	160582340	rs9456505	0,1863	0,1832	0,1835	0,0003	0,1839	0,0007	**0,0019**	**0,0038**	-0,0170	0,0008
6	160635886	rs10945656	0,2260	0,2482	0,2479	-0,0003	0,2476	-0,0006	**-0,0012**	**-0,0025**	0,0896	-0,0008
6	160637239	rs596881	0,3174	0,3790	0,3954	0,0165	0,4119	0,0329	**0,0416**	**0,0799**	0,1625	0,0530
6	160672625	rs316013	0,4588	0,4644	0,4832	0,0188	0,5020	0,0376	**0,0389**	**0,0748**	0,0119	0,0701
6	160681393	rs3127573	0,2210	0,2306	0,2322	0,0016	0,2339	0,0032	**0,0070**	**0,0139**	0,0416	0,0042
6	160682897	rs7757997	0,2586	0,2650	0,2652	0,0002	0,2654	0,0004	**0,0007**	**0,0015**	0,0241	0,0005
6	160687866	rs316030	0,3127	0,3219	0,3300	0,0081	0,3381	0,0161	**0,0244**	**0,0477**	0,0286	0,0238
6	160699534	rs2619276	0,3309	0,3300	0,3293	-0,0007	0,3286	-0,0014	**-0,0021**	**-0,0041**	-0,0029	-0,0020
7	87103670	rs2888611	0,4894	0,4467	0,4892	0,0425	0,5317	0,0850	**0,0869**	**0,1599**	-0,0956	0,1536
7	87154646	rs10225473	0,2696	0,2780	0,2786	0,0006	0,2792	0,0012	**0,0022**	**0,0044**	0,0302	0,0017
7	87163016	rs11760837	0,2677	0,2950	0,3043	0,0093	0,3137	0,0186	**0,0306**	**0,0594**	0,0927	0,0265
7	87179143	rs2235033	0,5057	0,5013	0,5006	-0,0006	0,5000	-0,0013	**-0,0013**	**-0,0026**	-0,0088	-0,0026
7	87179809	rs2229109	0,0296	0,0358	0,0364	0,0006	0,0370	0,0012	**0,0163**	**0,0321**	0,1735	0,0012
7	87180198	rs10276036	0,3750	0,3751	0,4004	0,0253	0,4257	0,0506	**0,0632**	**0,1188**	0,0002	0,0809
7	87183354	rs1922240	0,3709	0,3911	0,3993	0,0082	0,4075	0,0164	**0,0206**	**0,0403**	0,0517	0,0270
7	87201482	rs10260862	0,4178	0,4224	0,4382	0,0157	0,4539	0,0314	**0,0359**	**0,0693**	0,0110	0,0544
7	87278760	rs10267099	0,2975	0,2933	0,3021	0,0088	0,3109	0,0177	**0,0292**	**0,0568**	-0,0142	0,0250
7	99207876	rs7792939	0,0526	0,0634	0,0655	0,0021	0,0677	0,0043	**0,0324**	**0,0628**	0,1701	0,0045
10	96581094	rs10786172	0,2057	0,2198	0,2412	0,0213	0,2625	0,0427	**0,0885**	**0,1626**	0,0641	0,0547
10	114711983	rs7094463	0,4760	0,4870	0,4886	0,0017	0,4903	0,0033	**0,0034**	**0,0068**	0,0225	0,0065
10	114732906	rs7901275	0,3957	0,4341	0,4414	0,0073	0,4487	0,0146	**0,0165**	**0,0325**	0,0885	0,0258
10	114754088	rs7901695	0,4677	0,4872	0,4924	0,0052	0,4975	0,0104	**0,0105**	**0,0208**	0,0400	0,0202
10	114756041	rs4506565	0,4564	0,4879	0,4933	0,0055	0,4988	0,0109	**0,0111**	**0,0219**	0,0644	0,0213
10	114767771	rs4132670	0,4181	0,4686	0,4954	0,0268	0,5223	0,0537	**0,0542**	**0,1028**	0,1078	0,1010
10	114788815	rs12243326	0,3308	0,4101	0,4168	0,0067	0,4235	0,0134	**0,0161**	**0,0317**	0,1934	0,0228
10	114821249	rs11196212	0,3288	0,3737	0,4023	0,0285	0,4308	0,0571	**0,0709**	**0,1324**	0,1201	0,0911
10	114855397	rs11196224	0,4893	0,4873	0,4882	0,0008	0,4890	0,0016	**0,0017**	**0,0033**	-0,0040	0,0032

Supplementary Table 2 next

Fst_TUN_LWK

Chr	Localisation	rs ID	Ho	Hs	Ht	Dst	Htp	Dstp	Fst	Fstp	Fis	Dest
10	114859463	rs7085532	0,3258	0,3315	0,3408	0,0093	0,3500	0,0185	**0,0272**	**0,0529**	0,0173	0,0277
10	114898093	rs3814573	0,2920	0,3174	0,3621	0,0446	0,4067	0,0893	**0,1233**	**0,2195**	0,0802	0,1308
10	114912534	rs1555485	0,0506	0,0566	0,0569	0,0003	0,0571	0,0005	**0,0045**	**0,0089**	0,1068	0,0005
11	2528003	rs11023096	0,3249	0,3136	0,3129	-0,0006	0,3123	-0,0013	**-0,0020**	**-0,0041**	-0,0362	-0,0019
11	2528233	rs4929992	0,4227	0,4284	0,4424	0,0140	0,4564	0,0280	**0,0316**	**0,0613**	0,0134	0,0490
11	2550730	rs179429	0,2733	0,2978	0,2983	0,0006	0,2989	0,0011	**0,0019**	**0,0037**	0,0821	0,0016
11	2553703	rs179435	0,4148	0,4480	0,4528	0,0048	0,4576	0,0096	**0,0106**	**0,0210**	0,0742	0,0174
11	2595287	rs2283171	0,4034	0,4131	0,4935	0,0804	0,5739	0,1609	**0,1630**	**0,2803**	0,0235	0,2741
11	2617782	rs1116714	0,4733	0,4598	0,4966	0,0369	0,5335	0,0737	**0,0742**	**0,1382**	-0,0294	0,1365
11	2633152	rs10766212	0,4158	0,4066	0,4487	0,0421	0,4908	0,0842	**0,0938**	**0,1715**	-0,0226	0,1419
11	2635797	rs2106467	0,4798	0,4856	0,4852	-0,0004	0,4848	-0,0008	**-0,0008**	**-0,0017**	0,0120	-0,0016
11	2673575	rs6578283	0,5146	0,4868	0,4877	0,0009	0,4886	0,0019	**0,0019**	**0,0038**	-0,0573	0,0036
11	2750703	rs170786	0,4744	0,4863	0,4969	0,0105	0,5074	0,0211	**0,0212**	**0,0416**	0,0245	0,0410
11	2776448	rs11023996	0,2006	0,2032	0,2076	0,0044	0,2120	0,0088	**0,0212**	**0,0415**	0,0129	0,0110
11	2782648	rs548566	0,2049	0,2349	0,2361	0,0012	0,2374	0,0025	**0,0052**	**0,0104**	0,1277	0,0032
11	2821065	rs163171	0,4163	0,4330	0,4718	0,0388	0,5106	0,0776	**0,0822**	**0,1519**	0,0386	0,1368
11	2837625	rs233446	0,2623	0,2838	0,2838	0,0001	0,2839	0,0001	**0,0002**	**0,0004**	0,0758	0,0002
11	2850782	rs234852	0,3914	0,4822	0,5009	0,0186	0,5195	0,0373	**0,0372**	**0,0717**	0,1884	0,0720
11	2895800	rs3987740	0,4030	0,3969	0,4058	0,0089	0,4147	0,0178	**0,0219**	**0,0430**	-0,0155	0,0295
11	17393644	rs12791318	0,2866	0,3178	0,3206	0,0028	0,3234	0,0057	**0,0088**	**0,0175**	0,0980	0,0083
11	17405333	rs10832785	0,4214	0,4687	0,4886	0,0199	0,5086	0,0399	**0,0408**	**0,0784**	0,1010	0,0751
11	17408025	rs2285676	0,4358	0,4644	0,4879	0,0235	0,5115	0,0470	**0,0482**	**0,0919**	0,0617	0,0878
11	17408630	rs5215	0,0980	0,1499	0,1566	0,0067	0,1634	0,0135	**0,0430**	**0,0825**	0,3463	0,0159
11	17408831	rs1800467	0,0219	0,0215	0,0217	0,0002	0,0219	0,0004	**0,0088**	**0,0174**	-0,0177	0,0004
11	17438890	rs2074315	0,3802	0,4074	0,4584	0,0509	0,5093	0,1019	**0,1111**	**0,2000**	0,0668	0,1719
11	17441828	rs4757517	0,4832	0,4284	0,4975	0,0691	0,5667	0,1383	**0,1390**	**0,2440**	-0,1280	0,2419
11	17496516	rs1048099	0,4890	0,4873	0,4997	0,0124	0,5121	0,0248	**0,0248**	**0,0483**	-0,0034	0,0483
11	17510419	rs11603988	0,1034	0,0970	0,0982	0,0012	0,0994	0,0024	**0,0122**	**0,0240**	-0,0653	0,0026
11	17510565	rs4757527	0,4029	0,4347	0,4687	0,0339	0,5026	0,0679	**0,0724**	**0,1350**	0,0732	0,1201
11	17530484	rs7104083	0,4154	0,4357	0,4467	0,0111	0,4578	0,0221	**0,0247**	**0,0483**	0,0465	0,0392
11	17532597	rs1076311	0,3576	0,3745	0,4042	0,0297	0,4339	0,0594	**0,0734**	**0,1368**	0,0452	0,0949
11	17542649	rs2041032	0,4428	0,4987	0,4975	-0,0012	0,4962	-0,0024	**-0,0024**	**-0,0049**	0,1119	-0,0048
11	108097333	rs228591	0,4365	0,4525	0,5002	0,0477	0,5478	0,0953	**0,0953**	**0,1740**	0,0353	0,1741
11	108268286	rs7931930	0,3947	0,4021	0,4645	0,0624	0,5268	0,1247	**0,1343**	**0,2367**	0,0186	0,2086
11	108283161	rs11212617	0,3659	0,3894	0,4823	0,0929	0,5752	0,1859	**0,1927**	**0,3232**	0,0603	0,3044
16	31102321	rs7294	0,4005	0,4178	0,4390	0,0212	0,4602	0,0424	**0,0483**	**0,0921**	0,0415	0,0728
17	19447016	rs2440155	0,3158	0,3279	0,3272	-0,0007	0,3264	-0,0015	**-0,0023**	**-0,0045**	0,0368	-0,0022
17	19459537	rs2244280	0,2421	0,3080	0,3082	0,0002	0,3084	0,0004	**0,0007**	**0,0014**	0,2137	0,0006
17	19484951	rs2453594	0,3426	0,3698	0,3717	0,0018	0,3735	0,0036	**0,0049**	**0,0097**	0,0738	0,0058
17	19622643	rs11656096	0,1225	0,1325	0,1350	0,0025	0,1374	0,0049	**0,0182**	**0,0358**	0,0758	0,0057
17	19642952	rs2228100	0,4606	0,4798	0,4914	0,0116	0,5030	0,0233	**0,0237**	**0,0462**	0,0400	0,0447
17	19645938	rs887241	0,4517	0,4788	0,4860	0,0072	0,4932	0,0144	**0,0148**	**0,0292**	0,0566	0,0276
22	42152988	rs17377643	0,3209	0,3184	0,3367	0,0183	0,3550	0,0366	**0,0544**	**0,1032**	-0,0079	0,0537
22	42178441	rs126092	0,3804	0,4549	0,4970	0,0421	0,5392	0,0843	**0,0848**	**0,1563**	0,1637	0,1546
22	46235677	rs1023470	0,3164	0,3300	0,3309	0,0009	0,3318	0,0018	**0,0027**	**0,0054**	0,0410	0,0027
22	46238069	rs8141212	0,3734	0,3696	0,3867	0,0170	0,4037	0,0341	**0,0441**	**0,0844**	-0,0102	0,0541
22	46525794	rs6007919	0,2814	0,3284	0,4761	0,1477	0,6238	0,2955	**0,3103**	**0,4736**	0,1429	0,4399
22	46629479	rs4253776	0,3623	0,3891	0,4580	0,0689	0,5269	0,1379	**0,1505**	**0,2616**	0,0688	0,2257
22	46637254	rs9626814	0,3251	0,3802	0,4176	0,0374	0,4550	0,0748	**0,0896**	**0,1644**	0,1450	0,1207
22	46643774	rs16995069	0,2431	0,2639	0,2754	0,0115	0,2870	0,0231	**0,0419**	**0,0804**	0,0786	0,0313
22	46670394	rs6007761	0,3372	0,3554	0,4864	0,1310	0,6174	0,2620	**0,2694**	**0,4244**	0,0511	0,4065

	Ho	Hs	Ht	Dst	Htp	Dstp	Fst	Fstp	Fis	Dest
**$overall**	**Ho**	**Hs**	**Ht**	**Dst**	**Htp**	**Dstp**	**Fst**	**Fstp**	**Fis**	**Dest**
	0,3370	0,3520	0,3742	0,0223	0,3958	0,0449	**0,0595**	**0,1135**	0,0425	0,0693
**$FST**	0,1118									
**$FIS**	0,0480									

Supplementary Table 2 next

Fst Comparison among Tunisian and Esan populations

Chr	Localisation	rs ID	Ho	Hs	Ht	Dst	Htp	Dstp	Fst	Fstp	Fis	Dest
1	65381861	rs12563017	0,1295	0,1932	0,1928	-0,0004	0,1923	-0,0009	**-0,0023**	**-0,0045**	0,3296	-0,0011
1	65389835	rs10889503	0,3220		0,4970	0,1329	0,6299	0,2658	**0,2675**	**0,4220**	0,1155	0,4180
1	65421058	rs4916014	0,2519	0,3321	0,4841	0,1520	0,6360	0,3039	**0,3139**	**0,4779**	0,2415	0,4551
1	65427476	rs4915675		0,3373	0,4993	0,1620	0,6612	0,3239	**0,3244**	**0,4899**	0,1122	0,4888
1	65516055	rs6588109	0,3923	0,4551	0,4882	0,0331	0,5213	0,0662	**0,0678**	**0,1269**	0,1382	0,1215
1	65557876	rs6699671	0,3321	0,3451	0,3527	0,0077	0,3604	0,0153	**0,0217**	**0,0425**	0,0376	0,0234
1	65583858	rs11208591	0,2862	0,2596	0,3981	0,1385	0,5366	0,2769	**0,3478**	**0,5161**	-0,1024	0,3740
1	65619880	rs10789171	0,4145	0,4171	0,4498	0,0327	0,4824	0,0654	**0,0727**	**0,1355**	0,0061	0,1122
1	65658412	rs6677316	0,4611	0,4471	0,4677	0,0206	0,4882	0,0412	**0,0440**	**0,0843**	-0,0314	0,0745
3	12286720	rs9850825	0,4659	0,4733	0,5001	0,0268	0,5269	0,0536	**0,0536**	**0,1018**	0,0156	0,1018
3	12302462	rs9878908	0,1440	0,1466	0,1466	0,0001	0,1467	0,0001	**0,0004**	**0,0007**	0,0175	0,0001
3	12393125	rs1801282	0,0597	0,0629	0,0651	0,0021	0,0672	0,0042	**0,0324**	**0,0628**	0,0515	0,0045
3	12402474	rs1373641	0,3064	0,3149	0,3211	0,0062	0,3273	0,0125	**0,0194**	**0,0381**	0,0269	0,0182
3	12475088	rs7626560	0,2744	0,2931	0,2924	-0,0007	0,2917	-0,0014	**-0,0023**	**-0,0047**	0,0636	-0,0019
3	151007310	rs9863983	0,3997	0,3946	0,4424	0,0477	0,4901	0,0955	**0,1079**	**0,1948**	-0,0127	0,1577
3	151041513	rs3971191	0,2146	0,2262	0,2305	0,0043	0,2348	0,0086	**0,0188**	**0,0368**	0,0511	0,0112
3	151053898	rs7644001	0,4410	0,4962	0,4997	0,0034	0,5031	0,0069	**0,0069**	**0,0137**	0,1113	0,0136
3	151090963	rs9859538	0,3006	0,3056	0,3800	0,0743	0,4543	0,1487	**0,1956**	**0,3272**	0,0165	0,2141
3	151112568	rs3732768	0,3665	0,4098	0,4272	0,0174	0,4446	0,0348	**0,0408**	**0,0783**	0,1058	0,0590
3	151128895	rs10935844	0,5133	0,4991	0,4980	-0,0011	0,4969	-0,0021	**-0,0022**	**-0,0043**	-0,0285	-0,0043
3	151147968	rs6772196	0,2348	0,2632	0,2682	0,0050	0,2732	0,0100	**0,0186**	**0,0365**	0,1078	0,0135
5	51405600	rs12655411	0,4015	0,3738	0,3795	0,0057	0,3852	0,0114	**0,0150**	**0,0296**	-0,0741	0,0182
5	51431680	rs10064799	0,3916	0,4056	0,4047	-0,0009	0,4038	-0,0019	**-0,0023**	**-0,0046**	0,0346	-0,0031
5	51505665	rs6865397	0,3939	0,3877	0,4697	0,0820	0,5517	0,1640	**0,1745**	**0,2972**	-0,0159	0,2678
5	51572584	rs4572960	0,4815	0,4803	0,4803	0,0000	NA	NA	**0,0000**	**NaN**	-0,0025	NA
5	74616843	rs10474433	0,4861	0,4911	0,4902	-0,0010	0,4892	-0,0019	**-0,0019**	**-0,0039**	0,0103	-0,0038
5	74620912	rs6878576	0,3035	0,3348	0,3482	0,0134	0,3616	0,0268	**0,0385**	**0,0741**	0,0933	0,0403
5	74625487	rs7703051	0,4136	0,4399	0,4396	-0,0003	0,4392	-0,0006	**-0,0007**	**-0,0015**	0,0597	-0,0011
5	74648603	rs12654264	0,4063	0,4372	0,4363	-0,0009	0,4354	-0,0018	**-0,0021**	**-0,0041**	0,0707	-0,0032
5	74651084	rs3846662	0,2583	0,2756	0,3967	0,1211	0,5178	0,2423	**0,3054**	**0,4679**	0,0625	0,3345
5	74655726	rs3846663	0,3317	0,3439	0,3556	0,0117	0,3674	0,0235	**0,0330**	**0,0639**	0,0355	0,0358
5	74656175	rs5909	0,0753	0,0849	0,0865	0,0016	0,0881	0,0032	**0,0184**	**0,0362**	0,1128	0,0035
6	160496055	rs3777406	0,3013	0,3022	0,3114	0,0092	0,3206	0,0184	**0,0295**	**0,0574**	0,0032	0,0264
6	160517481	rs1803989	0,3206	0,3010	0,3199	0,0188	0,3387	0,0377	**0,0589**	**0,1112**	-0,0649	0,0539
6	160528057	rs7753051	0,4071	0,4374	0,4674	0,0300	0,4974	0,0600	**0,0642**	**0,1206**	0,0694	0,1067
6	160572866	rs622342	0,2954	0,3114	0,3127	0,0013	0,3140	0,0026	**0,0041**	**0,0082**	0,0514	0,0038
6	160578860	rs1564348	0,2045	0,2125	0,2165	0,0040	0,2205	0,0080	**0,0185**	**0,0363**	0,0376	0,0102
6	160581374	rs651164	0,4122	0,3948	0,4180	0,0232	0,4411	0,0464	**0,0555**	**0,1051**	-0,0441	0,0766
6	160582340	rs9456505	0,1812	0,1870	0,1876	0,0005	0,1881	0,0010	**0,0028**	**0,0056**	0,0309	0,0013
6	160635886	rs10945656	0,2361	0,2683	0,2691	0,0008	0,2699	0,0016	**0,0030**	**0,0060**	0,1199	0,0022
6	160637239	rs596881	0,3427	0,3605	0,3682	0,0078	0,3760	0,0155	**0,0211**	**0,0413**	0,0494	0,0243
6	160672625	rs316013	0,4437	0,4645	0,4822	0,0178	0,5000	0,0355	**0,0368**	**0,0710**	0,0448	0,0663
6	160681393	rs3127573	0,1655	0,1824	0,1819	-0,0005	0,1815	-0,0009	**-0,0025**	**-0,0050**	0,0925	-0,0011
6	160682897	rs7757997	0,2131	0,2334	0,2329	-0,0005	0,2324	-0,0011	**-0,0023**	**-0,0046**	0,0870	-0,0014
6	160687866	rs316030	0,3986	0,3817	0,3815	-0,0002	0,3814	-0,0003	**-0,0004**	**-0,0008**	-0,0442	-0,0005
6	160699534	rs2619276	0,3107	0,2911	0,2918	0,0007	0,2925	0,0014	**0,0024**	**0,0047**	-0,0672	0,0019
7	87103670	rs2888611	0,4439	0,4513	0,4700	0,0187	0,4887	0,0374	**0,0398**	**0,0766**	0,0163	0,0682
7	87154646	rs10225473	0,2848	0,2748	0,2752	0,0004	0,2756	0,0008	**0,0015**	**0,0031**	-0,0364	0,0012
7	87163016	rs11760837	0,2778	0,2736	0,2781	0,0045	0,2826	0,0091	**0,0163**	**0,0321**	-0,0154	0,0125
7	87179143	rs2235033	0,5158	0,5011	0,5009	-0,0003	0,5006	-0,0006	**-0,0005**	**-0,0011**	-0,0293	-0,0011
7	87179809	rs2229109	0,0296	0,0358	0,0364	0,0006	0,0370	0,0012	**0,0163**	**0,0321**	0,1735	0,0012
7	87180198	rs10276036	0,4154	0,3883	0,4092	0,0209	0,4301	0,0418	**0,0511**	**0,0972**	-0,0698	0,0683
7	87183354	rs1922240	0,3506	0,3912	0,3993	0,0082	0,4075	0,0163	**0,0205**	**0,0401**	0,1036	0,0268
7	87201482	rs10260862	0,3926	0,4250	0,4435	0,0185	0,4620	0,0371	**0,0418**	**0,0802**	0,0762	0,0644
7	87278760	rs10267099	0,3732	0,3395	0,3414	0,0019	0,3433	0,0038	**0,0056**	**0,0112**	-0,0994	0,0058
7	99207876	rs7792939	0,0526	0,0634	0,0655	0,0021	0,0677	0,0043	**0,0324**	**0,0628**	0,1701	0,0045
10	96581094	rs10786172	0,1855	0,2006	0,2264	0,0258	0,2522	0,0516	**0,1140**	**0,2047**	0,0750	0,0646
10	114711983	rs7094463	0,5215	0,4856	0,4877	0,0022	0,4899	0,0043	**0,0044**	**0,0089**	-0,0739	0,0084
10	114732906	rs7901275	0,4159	0,4239	0,4342	0,0102	0,4444	0,0204	**0,0235**	**0,0459**	0,0189	0,0354
10	114754088	rs7901695	0,4626	0,4815	0,4815	0,0000	0,4815	0,0000	**0,0000**	**-0,0001**	0,0392	-0,0001
10	114756041	rs4506565	0,4564	0,4821	0,4819	-0,0001	0,4818	-0,0003	**-0,0003**	**-0,0006**	0,0532	-0,0006
10	114767771	rs4132670	0,3878	0,4573	0,4918	0,0345	0,5262	0,0689	**0,0701**	**0,1309**	0,1520	0,1270
10	114788815	rs12243326	0,3863	0,4450	0,4451	0,0001	0,4452	0,0002	**0,0002**	**0,0004**	0,1318	0,0003
10	114821249	rs11196212	0,3491	0,3665	0,3977	0,0311	0,4288	0,0622	**0,0782**	**0,1451**	0,0477	0,0982
10	114855397	rs11196224	0,4741	0,4885	0,4890	0,0005	0,4895	0,0010	**0,0010**	**0,0020**	0,0295	0,0019

Supplementary Table 2 next

Fst_TUN_ESN

Chr	Localisation	rs ID	Ho	Hs	Ht	Dst	Htp	Dstp	Fst	Fstp	Fis	Dest
10	114859463	rs7085532	0,3258	0,3386	0,3465	0,0078	0,3543	0,0157	**0,0226**	**0,0443**	0,0380	0,0237
10	114898093	rs3814573	0,2920	0,3089	0,3567	0,0478	0,4044	0,0956	**0,1340**	**0,2363**	0,0548	0,1383
10	114912534	rs1555485	0,0354	0,0416	0,0425	0,0008	0,0433	0,0017	**0,0195**	**0,0383**	0,1492	0,0017
11	2528003	rs11023096	0,2845	0,3010	0,3004	-0,0006	0,2998	-0,0012	**-0,0020**	**-0,0040**	0,0547	-0,0017
11	2528233	rs4929992	0,3873	0,3901	0,4177	0,0276	0,4453	0,0552	**0,0661**	**0,1239**	0,0071	0,0905
11	2550730	rs179429	0,3642	0,3246	0,3291	0,0045	0,3336	0,0089	**0,0136**	**0,0267**	-0,1220	0,0132
11	2553703	rs179435	0,4754	0,4530	0,4617	0,0087	0,4703	0,0174	**0,0188**	**0,0369**	-0,0495	0,0317
11	2595287	rs2283171	0,3478	0,4051	0,4916	0,0866	0,5782	0,1731	**0,1761**	**0,2994**	0,1413	0,2910
11	2617782	rs1116714	0,4177	0,4436	0,5007	0,0570	0,5577	0,1141	**0,1139**	**0,2045**	0,0584	0,2050
11	2633152	rs10766212	0,4663	0,4774	0,4864	0,0090	0,4953	0,0179	**0,0184**	**0,0362**	0,0233	0,0343
11	2635797	rs2106467	0,4242	0,4433	0,4483	0,0050	0,4533	0,0100	**0,0112**	**0,0221**	0,0430	0,0180
11	2673575	rs6578283	0,4995	0,4887	0,4972	0,0085	0,5057	0,0169	**0,0170**	**0,0335**	-0,0219	0,0331
11	2750703	rs170786	0,4795	0,4829	0,4995	0,0166	0,5160	0,0332	**0,0332**	**0,0643**	0,0071	0,0641
11	2776448	rs11023996	0,1854	0,1998	0,2037	0,0039	0,2076	0,0078	**0,0191**	**0,0375**	0,0722	0,0097
11	2782648	rs548566	0,1696	0,1878	0,1941	0,0063	0,2003	0,0126	**0,0324**	**0,0627**	0,0970	0,0155
11	2821065	rs163171	0,3658	0,4188	0,4912	0,0723	0,5635	0,1447	**0,1473**	**0,2567**	0,1266	0,2489
11	2837625	rs233446	0,2976	0,3007	0,3001	-0,0006	0,2995	-0,0012	**-0,0020**	**-0,0039**	0,0103	-0,0017
11	2850782	rs234852	0,4167	0,4830	0,5007	0,0177	0,5184	0,0354	**0,0353**	**0,0683**	0,1373	0,0684
11	2895800	rs3987740	0,3576	0,3753	0,3898	0,0145	0,4042	0,0289	**0,0371**	**0,0716**	0,0472	0,0463
11	17393644	rs12791318	0,3220	0,3548	0,3685	0,0137	0,3823	0,0275	**0,0373**	**0,0719**	0,0925	0,0426
11	17405333	rs10832785	0,4315	0,4983	0,5006	0,0023	0,5029	0,0046	**0,0045**	**0,0091**	0,1341	0,0091
11	17408025	rs2285676	0,4509	0,4902	0,4983	0,0081	0,5063	0,0161	**0,0162**	**0,0319**	0,0801	0,0317
11	17408630	rs5215	0,1030	0,1547	0,1608	0,0061	0,1669	0,0123	**0,0382**	**0,0735**	0,3339	0,0145
11	17408831	rs1800467	0,0219	0,0215	0,0217	0,0002	0,0219	0,0004	**0,0088**	**0,0174**	-0,0177	0,0004
11	17438890	rs2074315	0,3853	0,4060	0,4654	0,0594	0,5248	0,1188	**0,1277**	**0,2264**	0,0510	0,2000
11	17441828	rs4757517	0,3721	0,3669	0,4990	0,1321	0,6310	0,2641	**0,2647**	**0,4186**	-0,0142	0,4172
11	17496516	rs1048099	0,4031	0,4265	0,4906	0,0642	0,5548	0,1284	**0,1308**	**0,2313**	0,0548	0,2238
11	17510419	rs11603988	0,0781	0,0723	0,0752	0,0029	0,0781	0,0058	**0,0386**	**0,0743**	-0,0803	0,0063
11	17510565	rs4757527	0,4080	0,4346	0,4699	0,0353	0,5052	0,0706	**0,0751**	**0,1397**	0,0614	0,1249
11	17530484	rs7104083	0,4255	0,4537	0,4593	0,0055	0,4648	0,0111	**0,0121**	**0,0238**	0,0622	0,0203
11	17532597	rs1076311	0,4030	0,4097	0,4271	0,0174	0,4446	0,0349	**0,0408**	**0,0785**	0,0162	0,0591
11	17542649	rs2041032	0,4681	0,5002	0,5009	0,0007	0,5017	0,0014	**0,0014**	**0,0029**	0,0642	0,0029
11	108097333	rs228591	0,4214	0,4667	0,5011	0,0344	0,5355	0,0688	**0,0687**	**0,1286**	0,0971	0,1291
11	108268286	rs7931930	0,3745	0,3957	0,4617	0,0660	0,5277	0,1320	**0,1429**	**0,2501**	0,0538	0,2184
11	108283161	rs11212617	0,3457	0,3830	0,4803	0,0974	0,5777	0,1947	**0,2027**	**0,3371**	0,0974	0,3156
16	31102321	rs7294	0,4005	0,4225	0,4556	0,0331	0,4886	0,0662	**0,0726**	**0,1354**	0,0521	0,1146
17	19447016	rs2440155	0,3057	0,3024	0,3024	0,0000	0,3025	0,0001	**0,0001**	**0,0002**	-0,0110	0,0001
17	19459537	rs2244280	0,2775	0,3242	0,3235	-0,0006	0,3229	-0,0012	**-0,0019**	**-0,0038**	0,1439	-0,0018
17	19484951	rs2453594	0,3274	0,3677	0,3691	0,0014	0,3705	0,0029	**0,0039**	**0,0077**	0,1095	0,0045
17	19622643	rs11656096	0,1225	0,1510	0,1521	0,0011	0,1532	0,0022	**0,0071**	**0,0141**	0,1891	0,0025
17	19642952	rs2228100	0,4252	0,4769	0,4801	0,0032	0,4834	0,0065	**0,0067**	**0,0134**	0,1083	0,0124
17	19645938	rs887241	0,4618	0,4993	0,4985	-0,0008	0,4977	-0,0016	**-0,0016**	**-0,0032**	0,0752	-0,0032
22	42152988	rs17377643	0,3259	0,3299	0,3453	0,0154	0,3607	0,0309	**0,0447**	**0,0856**	0,0119	0,0461
22	42178441	rs126092	0,4006	0,4585	0,4949	0,0364	0,5313	0,0728	**0,0736**	**0,1371**	0,1262	0,1345
22	46235677	rs1023470	0,2255	0,2513	0,2527	0,0014	0,2541	0,0028	**0,0055**	**0,0110**	0,1026	0,0037
22	46238069	rs8141212	0,4037	0,4404	0,4410	0,0006	0,4416	0,0012	**0,0014**	**0,0028**	0,0833	0,0022
22	46525794	rs6007919	0,2663	0,3165	0,4726	0,1561	0,6287	0,3122	**0,3304**	**0,4967**	0,1585	0,4568
22	46629479	rs4253776	0,3522	0,3891	0,4580	0,0689	0,5269	0,1378	**0,1504**	**0,2615**	0,0949	0,2256
22	46637254	rs9626814	0,3352	0,3832	0,4295	0,0463	0,4758	0,0925	**0,1077**	**0,1945**	0,1254	0,1500
22	46643774	rs16995069	0,2987	0,2852	0,3049	0,0197	0,3246	0,0394	**0,0646**	**0,1213**	-0,0474	0,0551
22	46670394	rs6007761	0,3019	0,3717	0,4756	0,1039	0,5794	0,2077	**0,2184**	**0,3585**	0,1878	0,3306

	Ho	Hs	Ht	Dst	Htp	Dstp	Fst	Fstp	Fis	Dest
**$overall**										
	0,3345	0,3523	0,3765	0,0243	0,4001	0,0490	**0,0644**	**0,1224**	0,0505	0,0756

$FST 0,1206

$FIS 0,0548

Supplementary Table 2 next

Fst Comparison among Tunisian and Gambian Mandinka populations

Chr	Localisation	rs ID	Ho	Hs	Ht	Dst	Htp	Dstp	Fst	Fstp	Fis	Dest
1	65381861	rs12563017	0,2074	0,2332	0,2354	0,0021	0,2375	0,0043	**0,0091**	**0,0181**	0,1109	0,0056
1	65389835	rs10889503	0,2805		0,4890	0,1684	0,6574	0,3367	**0,3443**	**0,5122**	0,1254	0,4957
1	65421058	rs4916014	0,2425	0,3230	0,4817	0,1587	0,6404	0,3173	**0,3294**	**0,4956**	0,2492	0,4688
1	65427476	rs4915675		0,3474	0,5000	0,1526	0,6527	0,3053	**0,3053**	**0,4677**	0,1011	0,4678
1	65516055	rs6588109	0,4684	0,4568	0,4830	0,0263	0,5093	0,0526	**0,0544**	**0,1032**	-0,0254	0,0968
1	65557876	rs6699671	0,4056	0,3637	0,3796	0,0159	0,3956	0,0319	**0,0420**	**0,0806**	-0,1154	0,0501
1	65583858	rs11208591	0,2982	0,2712	0,4035	0,1322	0,5357	0,2644	**0,3277**	**0,4936**	-0,0996	0,3628
1	65619880	rs10789171	0,3725	0,4168	0,4481	0,0314	0,4795	0,0627	**0,0700**	**0,1308**	0,1062	0,1075
1	65658412	rs6677316	0,4418	0,4343	0,4404	0,0061	0,4464	0,0121	**0,0138**	**0,0271**	-0,0173	0,0214
3	12286720	rs9850825	0,4890	0,4736	0,4738	0,0002	0,4740	0,0003	**0,0004**	**0,0007**	-0,0324	0,0007
3	12302462	rs9878908	0,1757	0,1731	0,1728	-0,0003	0,1725	-0,0007	**-0,0020**	**-0,0039**	-0,0148	-0,0008
3	12393125	rs1801282	0,0597	0,0629	0,0650	0,0021	0,0672	0,0042	**0,0326**	**0,0631**	0,0512	0,0045
3	12402474	rs1373641	0,3091	0,3212	0,3265	0,0052	0,3317	0,0105	**0,0161**	**0,0316**	0,0378	0,0154
3	12475088	rs7626560	0,2986	0,2750	0,2749	-0,0001	0,2747	-0,0003	**-0,0005**	**-0,0010**	-0,0858	-0,0004
3	151007310	rs9863983	0,3836	0,3924	0,4303	0,0379	0,4682	0,0758	**0,0880**	**0,1618**	0,0226	0,1247
3	151041513	rs3971191	0,2559	0,2420	0,2493	0,0073	0,2566	0,0146	**0,0293**	**0,0569**	-0,0573	0,0193
3	151053898	rs7644001	0,4659	0,4925	0,5010	0,0085	0,5095	0,0170	**0,0170**	**0,0334**	0,0539	0,0335
3	151090963	rs9859538	0,3152	0,3331	0,3954	0,0623	0,4577	0,1246	**0,1576**	**0,2723**	0,0536	0,1868
3	151112568	rs3732768	0,3610	0,3905	0,3967	0,0063	0,4030	0,0125	**0,0158**	**0,0311**	0,0753	0,0206
3	151128895	rs10935844	0,4858	0,4991	0,4980	-0,0011	0,4970	-0,0021	**-0,0021**	**-0,0042**	0,0266	-0,0042
3	151147968	rs6772196	0,2382	0,2500	0,2530	0,0030	0,2560	0,0060	**0,0119**	**0,0235**	0,0470	0,0080
5	51405600	rs12655411	0,3941	0,4298	0,4289	-0,0010	0,4279	-0,0019	**-0,0022**	**-0,0045**	0,0831	-0,0034
5	51431680	rs10064799	0,3950	0,4375	0,4400	0,0025	0,4425	0,0050	**0,0057**	**0,0113**	0,0972	0,0089
5	51505665	rs6865397	0,3695	0,4155	0,4801	0,0645	0,5446	0,1291	**0,1344**	**0,2370**	0,1109	0,2208
5	51572584	rs4572960	0,4815	0,4803	0,4803	0,0000	NA	NA	**0,0000**	**NaN**	-0,0025	NA
5	74616843	rs10474433	0,4750	0,4653	0,4685	0,0032	0,4717	0,0063	**0,0068**	**0,0134**	-0,0207	0,0118
5	74620912	rs6878576	0,2260	0,2458	0,2452	-0,0005	0,2447	-0,0011	**-0,0022**	**-0,0044**	0,0803	-0,0014
5	74625487	rs7703051	0,3918	0,4228	0,4219	-0,0009	0,4210	-0,0018	**-0,0021**	**-0,0042**	0,0734	-0,0030
5	74648603	rs12654264	0,3851	0,4115	0,4116	0,0002	0,4118	0,0004	**0,0005**	**0,0009**	0,0641	0,0006
5	74651084	rs3846662	0,2818	0,2972	0,4071	0,1099	0,5170	0,2197	**0,2699**	**0,4251**	0,0521	0,3127
5	74655726	rs3846663	0,3673	0,3877	0,3908	0,0031	0,3939	0,0062	**0,0079**	**0,0156**	0,0528	0,0101
5	74656175	rs5909	0,0778	0,0873	0,0888	0,0015	0,0902	0,0029	**0,0164**	**0,0322**	0,1084	0,0032
6	160496055	rs3777406	0,2276	0,2452	0,2455	0,0003	0,2459	0,0006	**0,0013**	**0,0026**	0,0720	0,0009
6	160517481	rs1803989	0,3145	0,2908	0,3053	0,0145	0,3198	0,0290	**0,0476**	**0,0908**	-0,0817	0,0410
6	160528057	rs7753051	0,4023	0,4302	0,4437	0,0135	0,4572	0,0270	**0,0304**	**0,0591**	0,0648	0,0474
6	160572866	rs622342	0,3057	0,3381	0,3376	-0,0005	0,3371	-0,0011	**-0,0016**	**-0,0031**	0,0958	-0,0016
6	160578860	rs1564348	0,2539	0,2443	0,2454	0,0011	0,2464	0,0021	**0,0043**	**0,0085**	-0,0394	0,0028
6	160581374	rs651164	0,3707	0,3767	0,4063	0,0297	0,4360	0,0593	**0,0730**	**0,1360**	0,0158	0,0951
6	160582340	rs9456505	0,2212	0,2075	0,2098	0,0022	0,2120	0,0044	**0,0105**	**0,0208**	-0,0658	0,0056
6	160635886	rs10945656	0,3178	0,3249	0,3377	0,0127	0,3504	0,0255	**0,0377**	**0,0728**	0,0219	0,0378
6	160637239	rs596881	0,3455	0,3643	0,3735	0,0092	0,3828	0,0185	**0,0247**	**0,0483**	0,0517	0,0291
6	160672625	rs316013	0,4598	0,4592	0,4933	0,0341	0,5275	0,0683	**0,0692**	**0,1294**	-0,0012	0,1262
6	160681393	rs3127573	0,2307	0,2458	0,2493	0,0034	0,2527	0,0069	**0,0138**	**0,0273**	0,0615	0,0091
6	160682897	rs7757997	0,3125	0,3166	0,3248	0,0082	0,3330	0,0164	**0,0252**	**0,0491**	0,0131	0,0239
6	160687866	rs316030	0,4096	0,3977	0,3969	-0,0008	0,3961	-0,0016	**-0,0020**	**-0,0041**	-0,0299	-0,0027
6	160699534	rs2619276	0,3077	0,2872	0,2882	0,0010	0,2892	0,0021	**0,0036**	**0,0071**	-0,0715	0,0029
7	87103670	rs2888611	0,4626	0,4510	0,4695	0,0186	0,4881	0,0371	**0,0396**	**0,0761**	-0,0257	0,0677
7	87154646	rs10225473	0,2774	0,2565	0,2562	-0,0003	0,2559	-0,0007	**-0,0013**	**-0,0026**	-0,0813	-0,0009
7	87163016	rs11760837	0,2591	0,2432	0,2440	0,0009	0,2449	0,0018	**0,0036**	**0,0073**	-0,0654	0,0023
7	87179143	rs2235033	0,5048	0,4924	0,4935	0,0010	0,4945	0,0021	**0,0021**	**0,0042**	-0,0251	0,0040
7	87179809	rs2229109	0,0341	0,0403	0,0407	0,0004	0,0411	0,0009	**0,0107**	**0,0213**	0,1541	0,0009
7	87180198	rs10276036	0,4219	0,4016	0,4182	0,0166	0,4349	0,0333	**0,0398**	**0,0765**	-0,0506	0,0556
7	87183354	rs1922240	0,3780	0,3862	0,3956	0,0094	0,4050	0,0188	**0,0238**	**0,0465**	0,0211	0,0307
7	87201482	rs10260862	0,4219	0,3997	0,4035	0,0038	0,4073	0,0076	**0,0094**	**0,0187**	-0,0556	0,0127
7	87278760	rs10267099	0,3564	0,3334	0,3361	0,0027	0,3387	0,0053	**0,0079**	**0,0157**	-0,0688	0,0080
7	99207876	rs7792939	0,0659	0,0765	0,0778	0,0013	0,0791	0,0026	**0,0167**	**0,0329**	0,1390	0,0028
10	96581094	rs10786172	0,1881	0,2030	0,2282	0,0253	0,2535	0,0505	**0,1107**	**0,1993**	0,0734	0,0634
10	114711983	rs7094463	0,4706	0,4776	0,4825	0,0049	0,4875	0,0098	**0,0102**	**0,0202**	0,0147	0,0188
10	114732906	rs7901275	0,4098	0,4266	0,4361	0,0095	0,4455	0,0189	**0,0217**	**0,0425**	0,0393	0,0330
10	114754088	rs7901695	0,4478	0,4768	0,4759	-0,0008	0,4751	-0,0016	**-0,0017**	**-0,0034**	0,0608	-0,0031
10	114756041	rs4506565	0,4239	0,4735	0,4724	-0,0011	0,4713	-0,0022	**-0,0023**	**-0,0046**	0,1047	-0,0041
10	114767771	rs4132670	0,3900	0,4489	0,4888	0,0399	0,5287	0,0798	**0,0816**	**0,1509**	0,1314	0,1448
10	114788815	rs12243326	0,4125	0,4723	0,4722	-0,0001	0,4722	-0,0001	**-0,0001**	**-0,0002**	0,1265	-0,0002
10	114821249	rs11196212	0,3100	0,3432	0,3828	0,0396	0,4224	0,0792	**0,1035**	**0,1876**	0,0966	0,1206
10	114855397	rs11196224	0,4864	0,4681	0,4753	0,0072	0,4824	0,0144	**0,0151**	**0,0298**	-0,0392	0,0270

Supplementary Table 2 next

Fst_TUN_GWD

Chr	Localisation	rs ID	Ho	Hs	Ht	Dst	Htp	Dstp	Fst	Fstp	Fis	Dest
10	114859463	rs7085532	0,3177	0,3494	0,3552	0,0059	0,3611	0,0117	**0,0165**	**0,0325**	0,0906	0,0180
10	114898093	rs3814573	0,2959	0,3195	0,3634	0,0439	0,4073	0,0878	**0,1208**	**0,2156**	0,0740	0,1290
10	114912534	rs1555485	0,0354	0,0416	0,0425	0,0008	0,0433	0,0017	**0,0197**	**0,0387**	0,1489	0,0017
11	2528003	rs11023096	0,2885	0,3279	0,3276	-0,0003	0,3273	-0,0005	**-0,0008**	**-0,0017**	0,1201	-0,0008
11	2528233	rs4929992	0,3104	0,3322	0,3820	0,0498	0,4319	0,0997	**0,1305**	**0,2308**	0,0655	0,1493
11	2550730	rs179429	0,2950	0,2955	0,2960	0,0005	0,2965	0,0010	**0,0017**	**0,0033**	0,0016	0,0014
11	2553703	rs179435	0,4208	0,4543	0,4641	0,0099	0,4740	0,0198	**0,0213**	**0,0417**	0,0737	0,0362
11	2595287	rs2283171	0,4264	0,4350	0,4980	0,0630	0,5610	0,1260	**0,1265**	**0,2247**	0,0198	0,2231
11	2617782	rs1116714	0,4477	0,4585	0,4972	0,0387	0,5359	0,0774	**0,0778**	**0,1444**	0,0236	0,1429
11	2633152	rs10766212	0,4363	0,4689	0,4817	0,0128	0,4946	0,0257	**0,0266**	**0,0519**	0,0696	0,0483
11	2635797	rs2106467	0,4169	0,4266	0,4362	0,0096	0,4458	0,0192	**0,0220**	**0,0431**	0,0228	0,0335
11	2673575	rs6578283	0,4758	0,4892	0,4931	0,0039	0,4970	0,0078	**0,0080**	**0,0158**	0,0274	0,0154
11	2750703	rs170786	0,4937	0,4854	0,4878	0,0024	0,4903	0,0049	**0,0050**	**0,0099**	-0,0170	0,0094
11	2776448	rs11023996	0,1281	0,1484	0,1483	0,0000	0,1483	-0,0001	**-0,0003**	**-0,0006**	0,1368	-0,0001
11	2782648	rs548566	0,2429	0,2485	0,2489	0,0004	0,2493	0,0008	**0,0015**	**0,0031**	0,0224	0,0010
11	2821065	rs163171	0,3446	0,4094	0,4953	0,0859	0,5811	0,1717	**0,1733**	**0,2955**	0,1584	0,2907
11	2837625	rs233446	0,3193	0,3064	0,3058	-0,0006	0,3052	-0,0012	**-0,0020**	**-0,0039**	-0,0422	-0,0017
11	2850782	rs234852	0,4328	0,4865	0,4993	0,0128	0,5121	0,0256	**0,0256**	**0,0500**	0,1104	0,0498
11	2895800	rs3987740	0,3837	0,3944	0,4039	0,0095	0,4135	0,0191	**0,0236**	**0,0461**	0,0272	0,0315
11	17393644	rs12791318	0,3197	0,3544	0,3681	0,0137	0,3817	0,0273	**0,0371**	**0,0716**	0,0979	0,0423
11	17405333	rs10832785	0,4678	0,5017	0,5008	-0,0008	0,5000	-0,0017	**-0,0017**	**-0,0034**	0,0676	-0,0034
11	17408025	rs2285676	0,4733	0,5000	0,5011	0,0011	0,5021	0,0021	**0,0021**	**0,0042**	0,0534	0,0042
11	17408630	rs5215	0,1037	0,1552	0,1613	0,0061	0,1674	0,0122	**0,0377**	**0,0727**	0,3320	0,0144
11	17408831	rs1800467	0,0264	0,0260	0,0261	0,0001	0,0262	0,0002	**0,0037**	**0,0074**	-0,0149	0,0002
11	17438890	rs2074315	0,4210	0,4029	0,4718	0,0689	0,5408	0,1379	**0,1461**	**0,2549**	-0,0449	0,2309
11	17441828	rs4757517	0,4115	0,3988	0,5008	0,1019	0,6027	0,2039	**0,2036**	**0,3383**	-0,0318	0,3392
11	17496516	rs1048099	0,4532	0,4865	0,5000	0,0135	0,5134	0,0269	**0,0269**	**0,0524**	0,0684	0,0524
11	17510419	rs11603988	0,0914	0,0855	0,0874	0,0019	0,0893	0,0039	**0,0222**	**0,0434**	-0,0696	0,0042
11	17510565	rs4757527	0,3628	0,4106	0,4151	0,0045	0,4197	0,0091	**0,0109**	**0,0216**	0,1165	0,0154
11	17530484	rs7104083	0,3986	0,4190	0,4356	0,0166	0,4522	0,0332	**0,0382**	**0,0735**	0,0486	0,0572
11	17532597	rs1076311	0,3748	0,4019	0,4220	0,0201	0,4421	0,0401	**0,0475**	**0,0907**	0,0675	0,0671
11	17542649	rs2041032	0,4141	0,4841	0,4974	0,0133	0,5106	0,0265	**0,0267**	**0,0519**	0,1447	0,0514
11	108097333	rs228591	0,4665	0,4660	0,5009	0,0350	0,5359	0,0700	**0,0698**	**0,1305**	-0,0011	0,1310
11	108268286	rs7931930	0,3797	0,3957	0,4617	0,0660	0,5277	0,1320	**0,1429**	**0,2501**	0,0405	0,2184
11	108283161	rs11212617	0,3509	0,3830	0,4803	0,0973	0,5776	0,1947	**0,2027**	**0,3370**	0,0837	0,3155
16	31102321	rs7294	0,3718	0,4205	0,4466	0,0261	0,4726	0,0521	**0,0584**	**0,1103**	0,1159	0,0900
17	19447016	rs2440155	0,2699	0,2752	0,2775	0,0023	0,2797	0,0045	**0,0082**	**0,0162**	0,0193	0,0063
17	19459537	rs2244280	0,2587	0,3088	0,3090	0,0002	0,3092	0,0005	**0,0008**	**0,0015**	0,1621	0,0007
17	19484951	rs2453594	0,3428	0,3699	0,3718	0,0019	0,3737	0,0038	**0,0051**	**0,0102**	0,0731	0,0060
17	19622643	rs11656096	0,1004	0,1114	0,1158	0,0044	0,1202	0,0088	**0,0381**	**0,0734**	0,0986	0,0099
17	19642952	rs2228100	0,4097	0,4796	0,4872	0,0076	0,4947	0,0151	**0,0155**	**0,0305**	0,1457	0,0290
17	19645938	rs887241	0,3913	0,4720	0,4821	0,0101	0,4922	0,0202	**0,0210**	**0,0411**	0,1710	0,0383
22	42152988	rs17377643	0,3918	0,3931	0,3956	0,0025	0,3980	0,0050	**0,0063**	**0,0125**	0,0032	0,0082
22	42178441	rs126092	0,4066	0,4598	0,4936	0,0338	0,5275	0,0676	**0,0685**	**0,1282**	0,1157	0,1252
22	46235677	rs1023470	0,2156	0,2504	0,2519	0,0015	0,2534	0,0030	**0,0059**	**0,0118**	0,1391	0,0040
22	46238069	rs8141212	0,3919	0,4006	0,4094	0,0088	0,4182	0,0176	**0,0215**	**0,0421**	0,0217	0,0293
22	46525794	rs6007919	0,3094	0,3472	0,4814	0,1342	0,6156	0,2685	**0,2788**	**0,4361**	0,1088	0,4112
22	46629479	rs4253776	0,4024	0,3840	0,4155	0,0314	0,4469	0,0629	**0,0757**	**0,1407**	-0,0479	0,1021
22	46637254	rs9626814	0,3582	0,3601	0,3770	0,0169	0,3938	0,0338	**0,0448**	**0,0857**	0,0051	0,0528
22	46643774	rs16995069	0,2794	0,3020	0,3315	0,0294	0,3609	0,0589	**0,0888**	**0,1632**	0,0750	0,0844
22	46670394	rs6007761	0,3508	0,3799	0,4641	0,0843	0,5484	0,1685	**0,1815**	**0,3073**	0,0765	0,2718

$overall	Ho	Hs	Ht	Dst	Htp	Dstp	Fst	Fstp	Fis	Dest
	0,3378	0,3536	0,3753	0,0217	0,3963	0,0438	**0,0578**	**0,1105**	0,0445	0,0678

$FST 0,1092

$FIS 0,0467

Fst Comparison among Tunisian and Mende populations

Chr	Localisation	rs ID	Ho	Hs	Ht	Dst	Htp	Dstp	Fst	Fstp	Fis	Dest
1	65381861	rs12563017	0,1672	0,2076	0,2076	0,0000	0,2077	0,0001	**0,0002**	**0,0004**	0,1946	0,0001
1	65389835	rs10889503	0,2478		0,4801	0,1989	0,6790	0,3978	**0,4143**	**0,5859**	0,1188	0,5534
1	65421058	rs4916014	0,1878	0,2626	0,4649	0,2022	0,6671	0,4045	**0,4350**	**0,6063**	0,2848	0,5485
1	65427476	rs4915675		0,2935	0,4937	0,2002	0,6939	0,4003	**0,4054**	**0,5770**	0,0981	0,5667
1	65516055	rs6588109	0,4911	0,4574	0,4800	0,0225	0,5025	0,0451	**0,0469**	**0,0897**	-0,0735	0,0830
1	65557876	rs6699671	0,3637	0,3405	0,3469	0,0064	0,3533	0,0127	**0,0184**	**0,0361**	-0,0680	0,0193
1	65583858	rs11208591	0,2996	0,2728	0,4042	0,1314	0,5356	0,2628	**0,3251**	**0,4907**	-0,0986	0,3614
1	65619880	rs10789171	0,3645	0,4134	0,4362	0,0229	0,4591	0,0457	**0,0524**	**0,0996**	0,1183	0,0779
1	65658412	rs6677316	0,4573	0,4451	0,4602	0,0151	0,4753	0,0303	**0,0329**	**0,0637**	-0,0274	0,0545
3	12286720	rs9850825	0,4764	0,4822	0,4944	0,0122	0,5066	0,0244	**0,0247**	**0,0482**	0,0119	0,0472
3	12302462	rs9878908	0,1725	0,1802	0,1798	-0,0004	0,1794	-0,0007	**-0,0021**	**-0,0041**	0,0426	-0,0009
3	12393125	rs1801282	0,0597	0,0630	0,0651	0,0021	0,0672	0,0042	**0,0322**	**0,0624**	0,0519	0,0045
3	12402474	rs1373641	0,2911	0,2973	0,3066	0,0093	0,3159	0,0186	**0,0303**	**0,0589**	0,0210	0,0265
3	12475088	rs7626560	0,2658	0,2601	0,2606	0,0006	0,2612	0,0011	**0,0021**	**0,0043**	-0,0221	0,0015
3	151007310	rs9863983	0,3563	0,3884	0,4173	0,0289	0,4462	0,0578	**0,0692**	**0,1295**	0,0825	0,0945
3	151041513	rs3971191	0,2480	0,2445	0,2521	0,0077	0,2598	0,0153	**0,0304**	**0,0590**	-0,0146	0,0203
3	151053898	rs7644001	0,4137	0,4953	0,4945	-0,0008	0,4937	-0,0016	**-0,0016**	**-0,0032**	0,1647	-0,0032
3	151090963	rs9859538	0,3518	0,3574	0,4091	0,0517	0,4608	0,1035	**0,1265**	**0,2246**	0,0157	0,1610
3	151112568	rs3732768	0,3972	0,3787	0,3817	0,0029	0,3846	0,0059	**0,0077**	**0,0153**	-0,0487	0,0095
3	151128895	rs10935844	0,5675	0,4893	0,4906	0,0013	0,4919	0,0026	**0,0026**	**0,0053**	-0,1597	0,0051
3	151147968	rs6772196	0,2480	0,2413	0,2432	0,0019	0,2451	0,0038	**0,0079**	**0,0156**	-0,0280	0,0050
5	51405600	rs12655411	0,3996	0,3758	0,3811	0,0053	0,3864	0,0105	**0,0138**	**0,0273**	-0,0631	0,0169
5	51431680	rs10064799	0,4577	0,4196	0,4190	-0,0005	0,4185	-0,0011	**-0,0013**	**-0,0026**	-0,0908	-0,0018
5	51505665	rs6865397	0,4146	0,4290	0,4849	0,0559	0,5408	0,1118	**0,1153**	**0,2067**	0,0335	0,1957
5	51572584	rs4572960	0,4815	0,4803	0,4803	0,0000	NA	NA	**0,0000**	**NaN**	-0,0025	NA
5	74616843	rs10474433	0,5008	0,4738	0,4748	0,0010	0,4759	0,0021	**0,0022**	**0,0044**	-0,0570	0,0039
5	74620912	rs6878576	0,2124	0,2425	0,2419	-0,0007	0,2412	-0,0014	**-0,0028**	**-0,0056**	0,1241	-0,0018
5	74625487	rs7703051	0,4074	0,4375	0,4368	-0,0007	0,4361	-0,0014	**-0,0016**	**-0,0032**	0,0688	-0,0025
5	74648603	rs12654264	0,3992	0,4337	0,4326	-0,0011	0,4314	-0,0022	**-0,0026**	**-0,0052**	0,0794	-0,0039
5	74651084	rs3846662	0,2684	0,2852	0,4013	0,1162	0,5175	0,2323	**0,2895**	**0,4490**	0,0588	0,3250
5	74655726	rs3846663	0,3491	0,4107	0,4106	-0,0001	0,4104	-0,0002	**-0,0003**	**-0,0006**	0,1499	-0,0004
5	74656175	rs5909	0,1013	0,1096	0,1099	0,0003	0,1102	0,0006	**0,0027**	**0,0053**	0,0753	0,0007
6	160496055	rs3777406	0,2404	0,2460	0,2463	0,0003	0,2466	0,0006	**0,0012**	**0,0024**	0,0228	0,0008
6	160517481	rs1803989	0,2874	0,2866	0,2994	0,0128	0,3121	0,0255	**0,0426**	**0,0817**	-0,0028	0,0358
6	160528057	rs7753051	0,3545	0,4379	0,4704	0,0326	0,5030	0,0651	**0,0692**	**0,1294**	0,1903	0,1158
6	160572866	rs622342	0,2759	0,3097	0,3111	0,0014	0,3125	0,0027	**0,0044**	**0,0088**	0,1093	0,0040
6	160578860	rs1564348	0,2389	0,2511	0,2515	0,0005	0,2520	0,0009	**0,0018**	**0,0037**	0,0486	0,0012
6	160581374	rs651164	0,4380	0,4322	0,4429	0,0107	0,4536	0,0214	**0,0241**	**0,0471**	-0,0134	0,0376
6	160582340	rs9456505	0,1752	0,1666	0,1664	-0,0002	0,1662	-0,0005	**-0,0014**	**-0,0028**	-0,0514	-0,0006
6	160635886	rs10945656	0,2687	0,2933	0,2972	0,0039	0,3012	0,0078	**0,0132**	**0,0260**	0,0840	0,0111
6	160637239	rs596881	0,3271	0,3761	0,3905	0,0145	0,4050	0,0289	**0,0370**	**0,0714**	0,1301	0,0463
6	160672625	rs316013	0,4694	0,4611	0,4919	0,0309	0,5228	0,0618	**0,0628**	**0,1181**	-0,0180	0,1146
6	160681393	rs3127573	0,1964	0,1999	0,1997	-0,0002	0,1994	-0,0005	**-0,0012**	**-0,0025**	0,0174	-0,0006
6	160682897	rs7757997	0,3340	0,2995	0,3037	0,0042	0,3078	0,0084	**0,0138**	**0,0272**	-0,1153	0,0120
6	160687866	rs316030	0,3957	0,3839	0,3835	-0,0004	0,3831	-0,0008	**-0,0010**	**-0,0020**	-0,0309	-0,0012
6	160699534	rs2619276	0,3206	0,3003	0,3003	0,0000	0,3004	0,0001	**0,0001**	**0,0002**	-0,0675	0,0001
7	87103670	rs2888611	0,4662	0,4507	0,4675	0,0167	0,4842	0,0335	**0,0358**	**0,0691**	-0,0343	0,0609
7	87154646	rs10225473	0,2181	0,2311	0,2307	-0,0004	0,2302	-0,0009	**-0,0019**	**-0,0038**	0,0563	-0,0011
7	87163016	rs11760837	0,1909	0,2206	0,2201	-0,0004	0,2197	-0,0009	**-0,0020**	**-0,0040**	0,1344	-0,0011
7	87179143	rs2235033	0,5187	0,4986	0,5009	0,0023	0,5031	0,0045	**0,0045**	**0,0090**	-0,0404	0,0090
7	87179809	rs2229109	0,0355	0,0417	0,0421	0,0004	0,0425	0,0007	**0,0088**	**0,0174**	0,1493	0,0008
7	87180198	rs10276036	0,4244	0,4230	0,4331	0,0101	0,4431	0,0202	**0,0233**	**0,0455**	-0,0033	0,0349
7	87183354	rs1922240	0,3378	0,3762	0,3879	0,0118	0,3997	0,0235	**0,0303**	**0,0589**	0,1020	0,0377
7	87201482	rs10260862	0,3594	0,4067	0,4123	0,0056	0,4178	0,0111	**0,0135**	**0,0266**	0,1164	0,0187
7	87278760	rs10267099	0,2662	0,2711	0,2842	0,0130	0,2972	0,0261	**0,0459**	**0,0877**	0,0182	0,0358
7	99207876	rs7792939	0,0526	0,0634	0,0656	0,0021	0,0677	0,0042	**0,0322**	**0,0624**	0,1705	0,0045
10	96581094	rs10786172	0,1998	0,2143	0,2369	0,0226	0,2595	0,0451	**0,0953**	**0,1740**	0,0679	0,0575
10	114711983	rs7094463	0,4479	0,4773	0,4823	0,0050	0,4873	0,0099	**0,0103**	**0,0204**	0,0616	0,0190
10	114732906	rs7901275	0,4064	0,4235	0,4338	0,0103	0,4441	0,0206	**0,0237**	**0,0463**	0,0405	0,0357
10	114754088	rs7901695	0,4471	0,4775	0,4765	-0,0010	0,4756	-0,0019	**-0,0020**	**-0,0041**	0,0637	-0,0037
10	114756041	rs4506565	0,4350	0,4793	0,4783	-0,0009	0,4774	-0,0019	**-0,0020**	**-0,0039**	0,0924	-0,0036
10	114767771	rs4132670	0,3791	0,4342	0,4835	0,0493	0,5328	0,0986	**0,1020**	**0,1851**	0,1269	0,1743
10	114788815	rs12243326	0,4103	0,4772	0,4785	0,0013	0,4798	0,0026	**0,0027**	**0,0054**	0,1401	0,0050
10	114821249	rs11196212	0,3068	0,3363	0,3784	0,0422	0,4206	0,0843	**0,1114**	**0,2005**	0,0877	0,1270
10	114855397	rs11196224	0,4754	0,4522	0,4653	0,0131	0,4784	0,0262	**0,0281**	**0,0547**	-0,0513	0,0477

Supplementary Table 2 next

Fst_TUN_MSL

Chr	Localisation	rs ID	Ho	Hs	Ht	Dst	Htp	Dstp	Fst	Fstp	Fis	Dest
10	114859463	rs7085532	0,3129	0,3321	0,3412	0,0091	0,3503	0,0181	**0,0266**	**0,0518**	0,0578	0,0271
10	114898093	rs3814573	0,2515	0,2729	0,3341	0,0612	0,3953	0,1224	**0,1832**	**0,3097**	0,0784	0,1684
10	114912534	rs1555485	0,0354	0,0417	0,0425	0,0008	0,0433	0,0016	**0,0193**	**0,0379**	0,1496	0,0017
11	2528003	rs11023096	0,2910	0,3227	0,3221	-0,0006	0,3215	-0,0012	**-0,0019**	**-0,0039**	0,0983	-0,0018
11	2528233	rs4929992	0,3661	0,3701	0,4052	0,0351	0,4403	0,0702	**0,0866**	**0,1594**	0,0109	0,1114
11	2550730	rs179429	0,2857	0,2900	0,2900	0,0000	0,2900	0,0000	**0,0000**	**-0,0001**	0,0149	0,0000
11	2553703	rs179435	0,4245	0,4584	0,4805	0,0220	0,5025	0,0440	**0,0458**	**0,0876**	0,0741	0,0813
11	2595287	rs2283171	0,3375	0,3616	0,4796	0,1180	0,5977	0,2361	**0,2461**	**0,3950**	0,0667	0,3698
11	2617782	rs1116714	0,3661	0,4235	0,5008	0,0772	0,5780	0,1545	**0,1542**	**0,2673**	0,1357	0,2680
11	2633152	rs10766212	0,4180	0,4410	0,4668	0,0258	0,4926	0,0517	**0,0553**	**0,1049**	0,0521	0,0924
11	2635797	rs2106467	0,4281	0,4295	0,4382	0,0087	0,4469	0,0174	**0,0199**	**0,0390**	0,0032	0,0306
11	2673575	rs6578283	0,4721	0,4819	0,5009	0,0190	0,5199	0,0380	**0,0380**	**0,0732**	0,0202	0,0734
11	2750703	rs170786	0,4488	0,4848	0,4859	0,0012	0,4871	0,0023	**0,0024**	**0,0047**	0,0742	0,0045
11	2776448	rs11023996	0,1440	0,1542	0,1543	0,0001	0,1544	0,0002	**0,0007**	**0,0014**	0,0661	0,0003
11	2782648	rs548566	0,1661	0,1847	0,1914	0,0067	0,1980	0,0133	**0,0348**	**0,0672**	0,1008	0,0163
11	2821065	rs163171	0,3587	0,4084	0,4958	0,0874	0,5832	0,1748	**0,1762**	**0,2997**	0,1217	0,2954
11	2837625	rs233446	0,2865	0,2968	0,2963	-0,0006	0,2957	-0,0011	**-0,0019**	**-0,0038**	0,0350	-0,0016
11	2850782	rs234852	0,4306	0,4862	0,4998	0,0136	0,5134	0,0271	**0,0271**	**0,0528**	0,1145	0,0528
11	2895800	rs3987740	0,3817	0,4069	0,4134	0,0065	0,4198	0,0129	**0,0156**	**0,0308**	0,0619	0,0218
11	17393644	rs12791318	0,3309	0,3443	0,3536	0,0093	0,3629	0,0186	**0,0263**	**0,0513**	0,0390	0,0284
11	17405333	rs10832785	0,4479	0,5013	0,5013	0,0000	0,5014	0,0001	**0,0001**	**0,0002**	0,1064	0,0002
11	17408025	rs2285676	0,4859	0,4957	0,5001	0,0044	0,5046	0,0089	**0,0089**	**0,0176**	0,0198	0,0176
11	17408630	rs5215	0,1080	0,1594	0,1650	0,0055	0,1705	0,0110	**0,0334**	**0,0647**	0,3225	0,0131
11	17408831	rs1800467	0,0219	0,0216	0,0217	0,0002	0,0219	0,0004	**0,0086**	**0,0170**	-0,0173	0,0004
11	17438890	rs2074315	0,3554	0,4074	0,4609	0,0535	0,5145	0,1071	**0,1161**	**0,2081**	0,1275	0,1806
11	17441828	rs4757517	0,3844	0,3948	0,5009	0,1062	0,6071	0,2123	**0,2119**	**0,3497**	0,0262	0,3508
11	17496516	rs1048099	0,5247	0,4765	0,5010	0,0245	0,5255	0,0491	**0,0490**	**0,0934**	-0,1012	0,0937
11	17510419	rs11603988	0,0899	0,0840	0,0860	0,0020	0,0881	0,0040	**0,0234**	**0,0457**	-0,0697	0,0044
11	17510565	rs4757527	0,3268	0,3707	0,3698	-0,0010	0,3688	-0,0020	**-0,0026**	**-0,0053**	0,1184	-0,0031
11	17530484	rs7104083	0,3907	0,4148	0,4328	0,0180	0,4508	0,0360	**0,0416**	**0,0798**	0,0582	0,0615
11	17532597	rs1076311	0,3288	0,3639	0,3974	0,0335	0,4309	0,0670	**0,0843**	**0,1555**	0,0965	0,1053
11	17542649	rs2041032	0,4517	0,4870	0,4983	0,0114	0,5097	0,0228	**0,0228**	**0,0447**	0,0724	0,0444
11	108097333	rs228591	0,4613	0,4799	0,4987	0,0188	0,5175	0,0376	**0,0377**	**0,0727**	0,0387	0,0723
11	108268286	rs7931930	0,4204	0,4336	0,4779	0,0443	0,5222	0,0886	**0,0927**	**0,1697**	0,0302	0,1565
11	108283161	rs11212617	0,3917	0,4208	0,4915	0,0707	0,5622	0,1414	**0,1439**	**0,2516**	0,0692	0,2442
16	31102321	rs7294	0,3858	0,4030	0,4118	0,0088	0,4206	0,0176	**0,0213**	**0,0418**	0,0429	0,0294
17	19447016	rs2440155	0,2828	0,2891	0,2900	0,0009	0,2908	0,0018	**0,0031**	**0,0062**	0,0217	0,0025
17	19459537	rs2244280	0,2613	0,3107	0,3107	0,0000	0,3107	0,0000	**0,0000**	**0,0000**	0,1589	0,0000
17	19484951	rs2453594	0,3187	0,3750	0,3775	0,0025	0,3801	0,0051	**0,0067**	**0,0134**	0,1501	0,0081
17	19622643	rs11656096	0,1165	0,1270	0,1299	0,0029	0,1328	0,0058	**0,0223**	**0,0437**	0,0824	0,0066
17	19642952	rs2228100	0,4282	0,4802	0,4910	0,0108	0,5018	0,0216	**0,0220**	**0,0430**	0,1083	0,0415
17	19645938	rs887241	0,4261	0,4642	0,4777	0,0135	0,4912	0,0270	**0,0283**	**0,0550**	0,0822	0,0504
22	42152988	rs17377643	0,3266	0,3577	0,3666	0,0089	0,3756	0,0179	**0,0244**	**0,0476**	0,0869	0,0278
22	42178441	rs126092	0,3809	0,4541	0,4976	0,0435	0,5411	0,0870	**0,0874**	**0,1607**	0,1612	0,1593
22	46235677	rs1023470	0,1858	0,2193	0,2240	0,0047	0,2287	0,0094	**0,0210**	**0,0412**	0,1524	0,0121
22	46238069	rs8141212	0,4547	0,4076	0,4147	0,0071	0,4218	0,0142	**0,0171**	**0,0336**	-0,1154	0,0239
22	46525794	rs6007919	0,2972	0,3514	0,4826	0,1312	0,6139	0,2624	**0,2719**	**0,4275**	0,1542	0,4046
22	46629479	rs4253776	0,3913	0,3885	0,4599	0,0714	0,5314	0,1428	**0,1553**	**0,2688**	-0,0071	0,2336
22	46637254	rs9626814	0,3633	0,3842	0,4482	0,0639	0,5121	0,1279	**0,1427**	**0,2497**	0,0544	0,2077
22	46643774	rs16995069	0,2950	0,2927	0,3161	0,0234	0,3395	0,0467	**0,0739**	**0,1377**	-0,0077	0,0661
22	46670394	rs6007761	0,3074	0,3804	0,4644	0,0839	0,5483	0,1678	**0,1807**	**0,3061**	0,1920	0,2709

$overall	Ho	Hs	Ht	Dst	Htp	Dstp	Fst	Fstp	Fis	Dest
	0,3304	0,3477	0,3717	0,0239	0,3949	0,0483	**0,0644**	**0,1223**	0,0497	0,0740

**$FST**	0,1199
**$FIS**	0,0571

The table hilight the Fst comparison between Tunisian and different studied populations.
rs ID: Single Nucleotide polymorphism identifier, HI: Heterozygosity of an individual in its subpopulation, HS: theoretical heterozygosity of an individual, HT: Theoretical heterozygosity of an individual in the total population.
The coefficient of Genetic structure FIS inbreeding is calculated within the subpopulation by FIS = (HS – HI) / HT. The FST fixation index expresses the decrease in heterozygosity linked to the divergence between the subpopulation and the total population. FST = (HT – HS) / HT.

Supplementary Table 2 next

Supplementary table 3: Allele Frequency Distribution Among Studied Population

	rs622342		rs7294		rs5215		rs3846662	
	A	C*		C*	T	C*	G	A*
**TUN**	77,48	22,51	21,87	78,12	85,45	14,54	48,12	51,88
**SARD**	61,70	38,29	30,85	69,14	64,89	35,10	32,97	67,02
**N_ITA**	66,15	33,84	36,64	63,35	62,21	37,78	44,27	55,72
**C_ITA**	66,25	33,75	32,71	67,28	67,61	32,38	45,90	54,09
**S_ITA**	66,12	33,87	29,48	70,51	67,30	32,69	47,11	52,88
**TSI**	66,35	33,64	33,64	66,35	71,49	28,50	42,99	57,00
**IBS**	55,60	44,39	35,51	64,48	61,68	38,31	40,65	59,34
**CEU**	61,61	38,38	31,31	68,68	61,61	38,38	42,42	57,57
**GBR**	64,28	35,71	41,75	58,24	73,62	26,37	45,05	54,94
**FIN**	61,61	38,38	58,58	41,41	55,55	44,44	45,45	54,54
**YRI**	83,33	16,66	51,38	48,61	98,61	1,38	95,83	4,16
**LWK**	79,79	20,20	42,92	57,07	97,47	2,52	96,97	3,03
**ESN**	83,83	16,16	47,98	52,02	96,97	3,03	97,47	2,52
**GWD**	79,64	20,35	45,13	54,86	96,90	3,09	95,13	4,86
**MSL**	84,11	15,88	35,88	64,11	96,47	3,52	96,47	3,52

(*) Minor Allele
Frequencies are expressed in (%)

The table represents the allelic frequencies comparison of clinically relevant variant among studied populations

Supplementary table 4: Genotypic Frequency Distribution Among Studied Population

	rs622342			rs7294			rs5215			rs3846662		
	A/A	C/A	C/C	C/C	T/C	T/T	T/T	C/T	C/C	G/G	A/G	A/A
**TUN**	61,06	32,82	6,10	64,844	26,563	8,59	78,18	14,54	7,27	24,81	46,61	28,57
**SARD**	40,42	42,55	17,02	51,064	36,170	12,76	40,42	48,93	10,63	12,76	40,42	46,80
**N_ITA**	48,46	35,38	16,15	40,458	45,802	13,74	38,93	46,56	14,50	19,84	48,85	31,29
**C_ITA**	44,75	43,00	12,25	44,030	46,517	9,45	46,40	42,43	11,16	23,07	45,65	31,26
**S_ITA**	42,58	47,09	10,32	52,564	35,897	11,53	46,15	42,30	11,53	21,79	50,64	27,56
**TSI**	45,79	41,12	13,08	42,991	46,729	10,28	51,40	40,18	8,41	17,75	50,46	31,77
**IBS**	27,10	57,00	15,88	42,991	42,991	14,01	37,38	48,59	14,01	19,62	42,05	38,31
**CEU**	39,39	44,44	16,16	46,465	44,444	9,09	33,33	56,56	10,10	18,18	48,48	33,33
**GBR**	40,65	47,25	12,08	30,769	54,945	14,28	51,64	43,95	4,39	26,37	57,14	16,48
**FIN**	37,37	48,48	14,14	37,374	42,424	20,20	26,26	58,58	15,15	22,22	46,46	31,31
**YRI**	70,37	25,92	3,70	25,000	47,222	27,77	97,22	2,77	0,00	91,66	8,33	0,00
**LWK**	63,63	32,32	4,04	30,303	53,535	16,16	94,94	5,05	0,00	93,93	6,06	0,00
**ESN**	70,70	26,26	3,03	25,253	53,535	21,21	93,93	6,06	0,00	94,94	5,05	0,00
**GWD**	65,48	28,31	6,19	30,973	47,788	21,23	93,80	6,19	0,00	90,26	9,73	0,00
**MSL**	72,94	22,35	4,70	38,824	50,588	10,58	92,94	7,05	0,00	92,94	7,05	0,00

The table represents the genotypic frequencies comparison of clinically relevant variant among studied populations.
Frequencies are expressed in (%)

Supplementary Figure 1: Manhattan Plot Fst results among studied populations

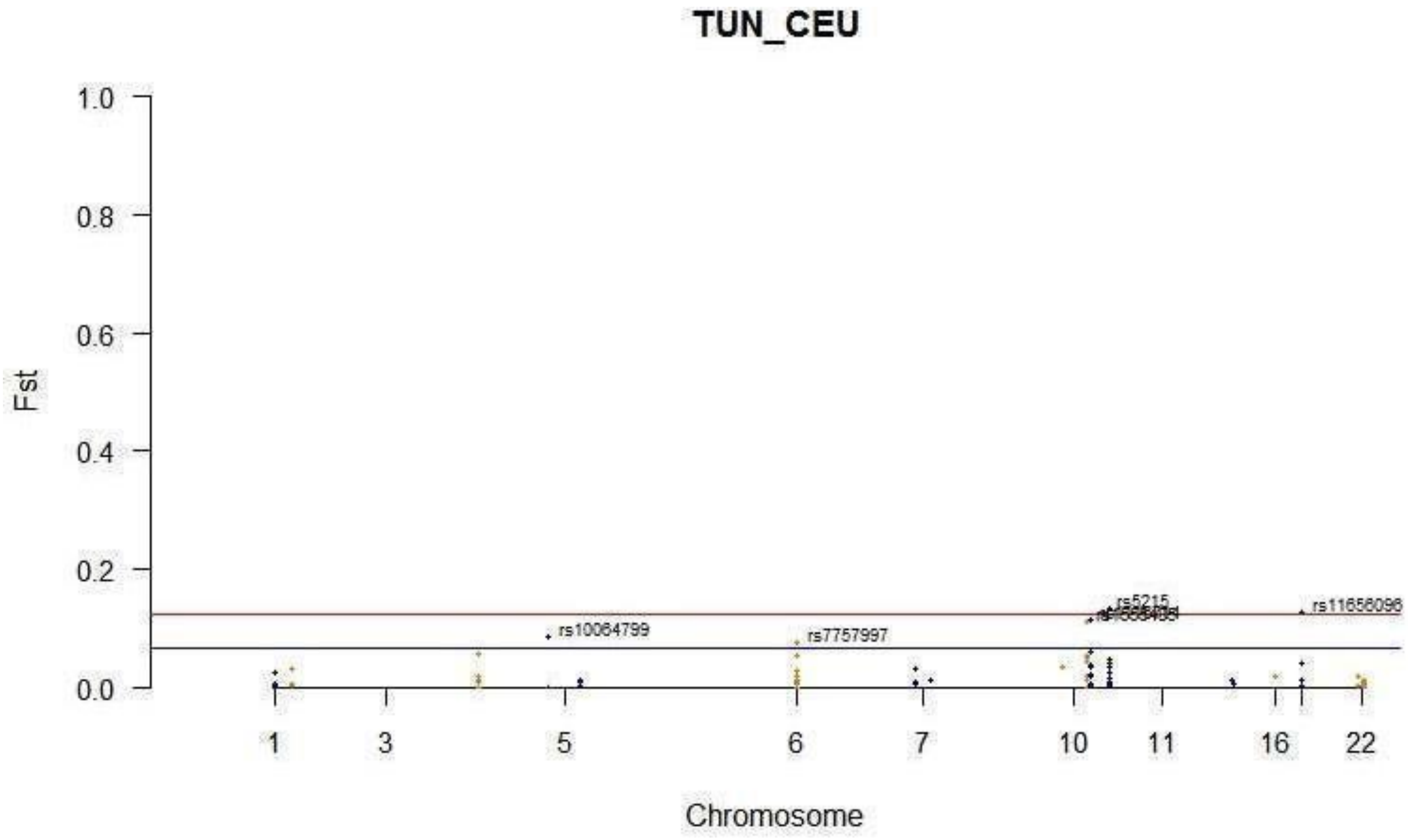

Manhattan plot of Fst comparison results. The X-axis represents the chromosomes; the Y-axis represents Fixation Index (Fst); a measure of population differentiation due to genetic structure; the horizontal blue line represents non differentiated variants; the horizontal red line points represent significant differentiated variants among Tunisian and Utah residents with Northern and Western European ancestry (CEU) populations.

Supplementary Figure 1 next

Next

Supplementary Figure 1: Manhattan Plot Fst result among studied populations

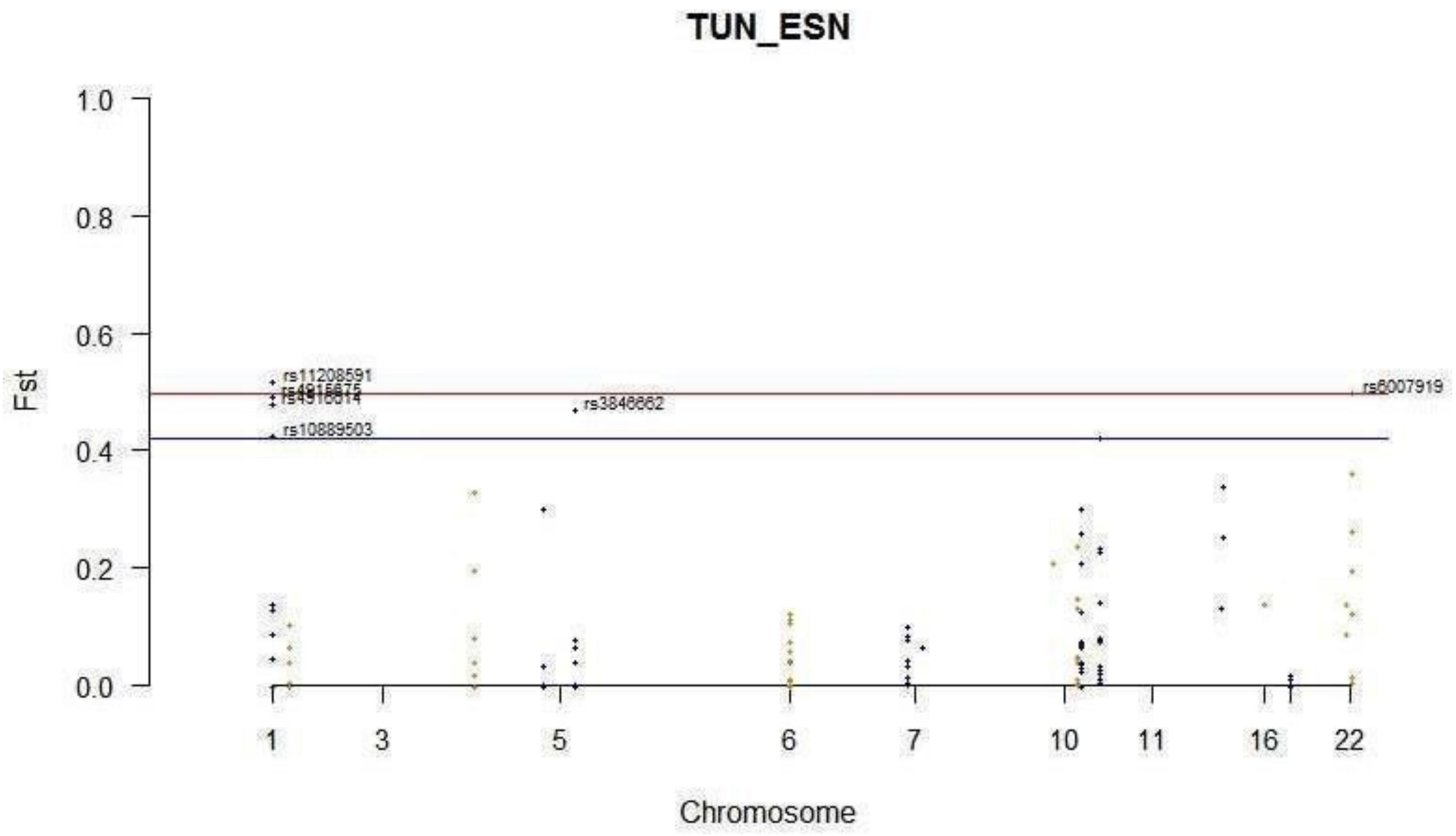

Manhattan plot of Fst comparison results. The X-axis represents the chromosomes; the Y-axis represents Fixation Index (Fst); a measure of population differentiation due to genetic structure; the horizontal blue line represents non differentiated variants; the horizontal red line points represent significant differentiated variants among Tunisian and Utah residents (CEPH) with Esan (ESN) populations.

Supplementary Figure 1 next

Supplementary Figure 1: Manhattan Plot Fst result among studied populations

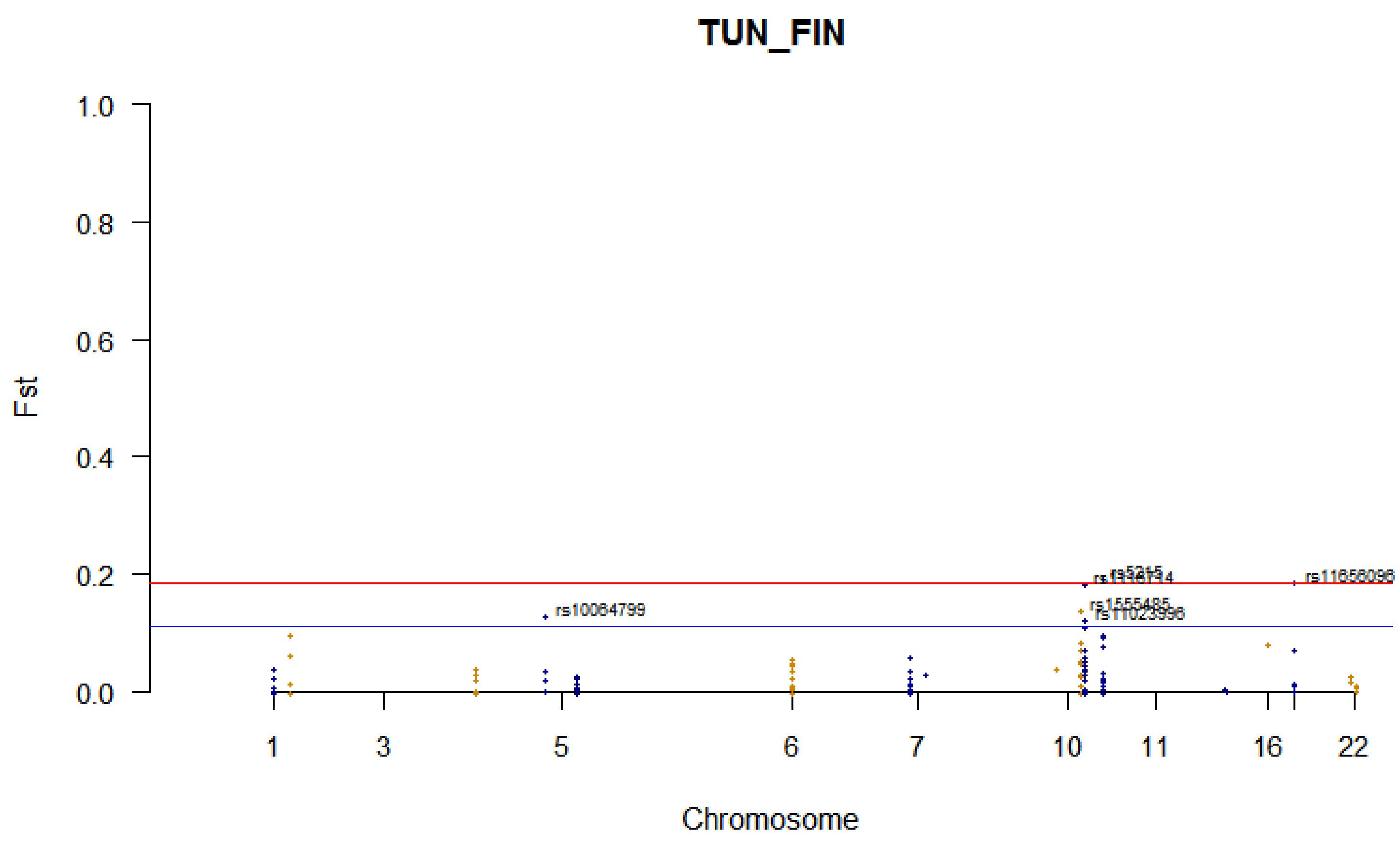

Manhattan plot of Fst comparison results. The X-axis represents the chromosomes; the Y-axis represents Fixation Index (Fst); a measure of population differentiation due to genetic structure; the horizontal blue line represents non differentiated variants; the horizontal red line points represent significant differentiated variants among Tunisian (TUN) and Finish (FIN) populations.

Supplementary Figure 1 next

Supplementary Figure 1: Manhattan Plot Fst result among studied populations

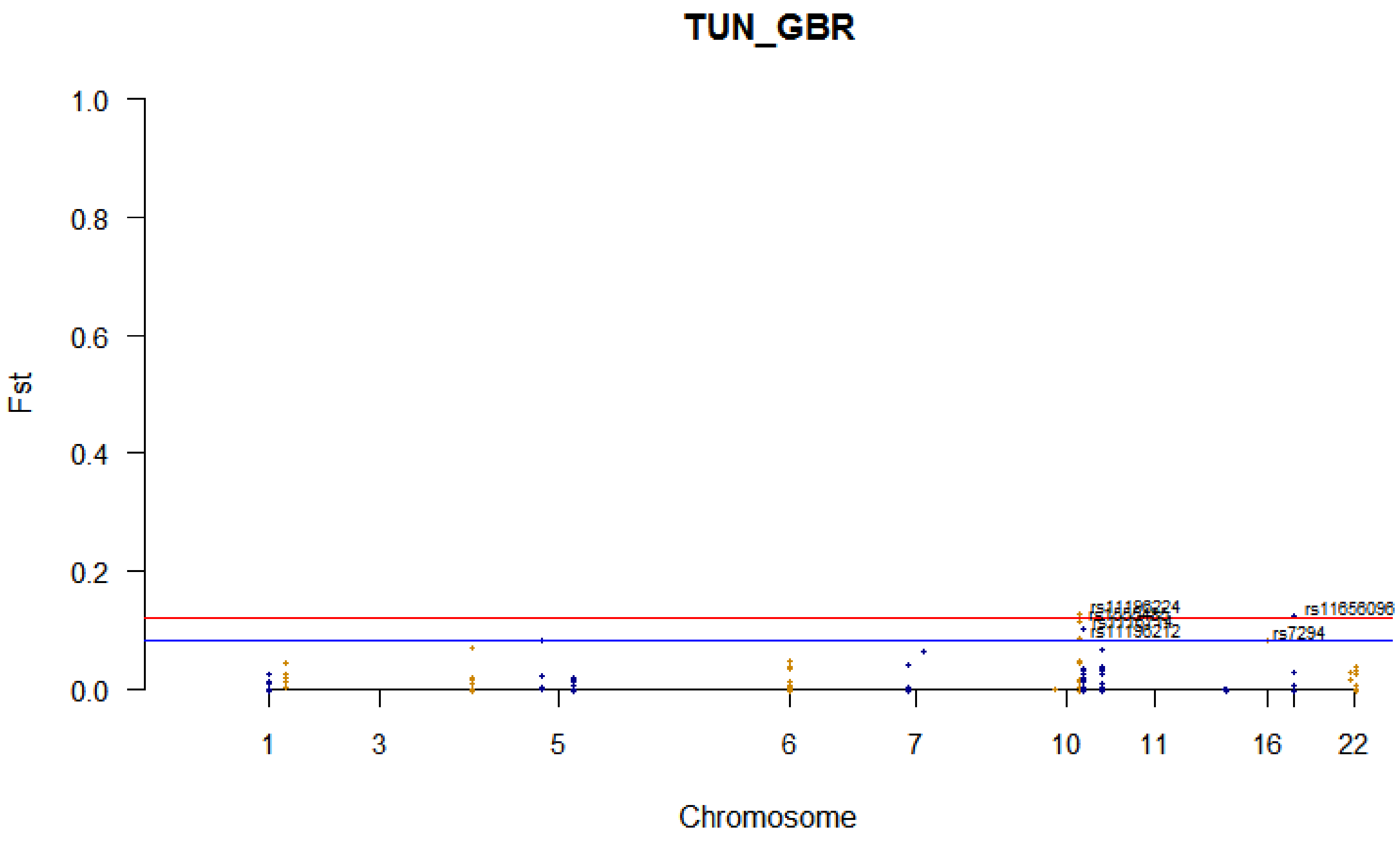

Manhattan plot of Fst comparison results. The X-axis represents the chromosomes; the Y-axis represents Fixation Index (Fst); a measure of population differentiation due to genetic structure; the horizontal blue line represents non differentiated variants; the horizontal red line points represent significant differentiated variants among Tunisian (TUN) and British (GBR) populations.

Supplementary Figure 1 next

Supplementary Figure 1: Manhattan Plot Fst result among studied populations

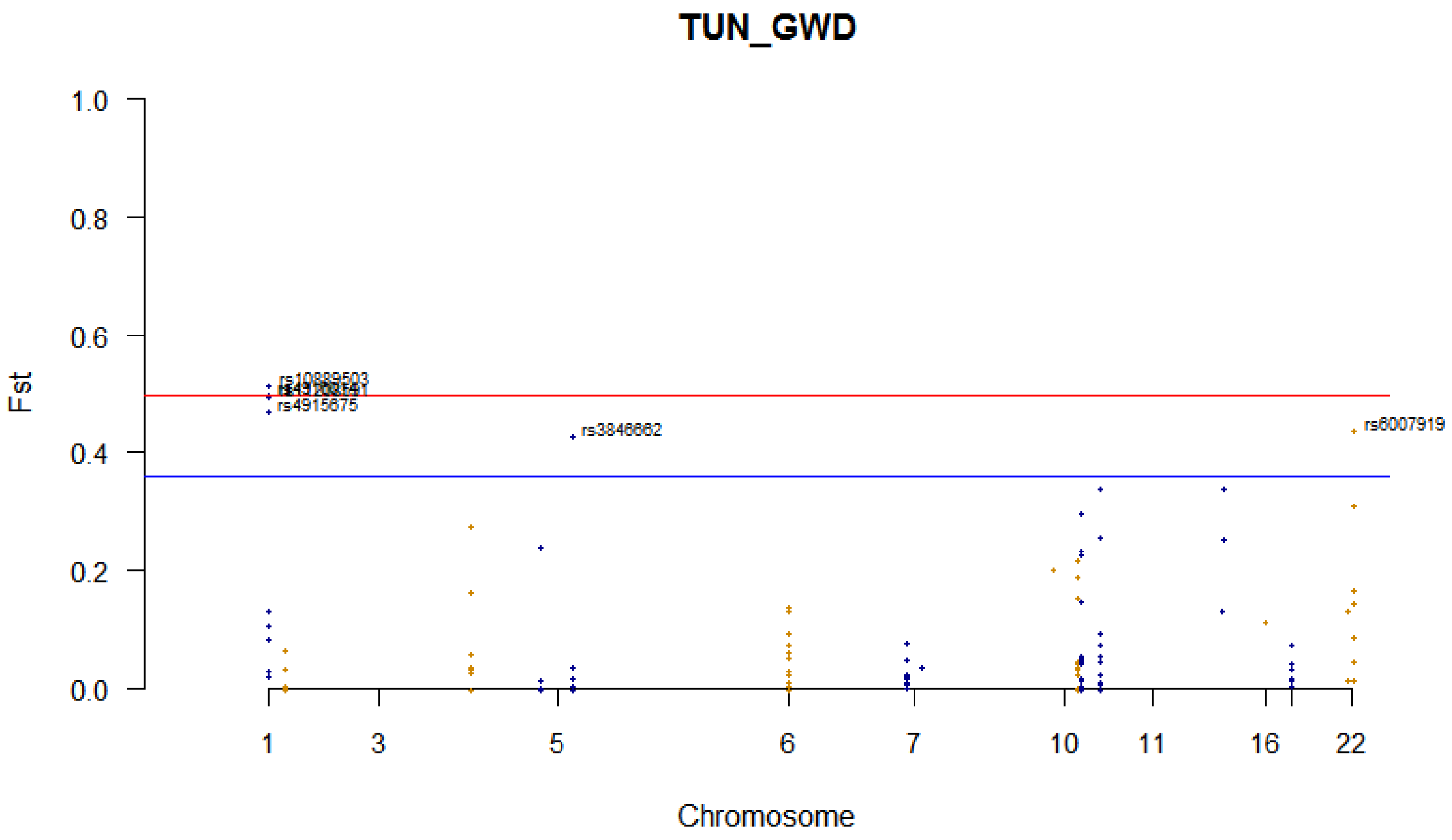

Manhattan plot of Fst comparison results. The X-axis represents the chromosomes; the Y-axis represents Fixation Index (Fst): a measure of population differentiation due to genetic structure; the horizontal blue line represents non differentiated variants; the horizontal red line points represent significant differentiated variants among Tunisian (TUN) and Gambian Mandinka (GWD) populations.

Supplementary Figure 1 next

Supplementary Figure 1: Manhattan Plot Fst result among studied populations

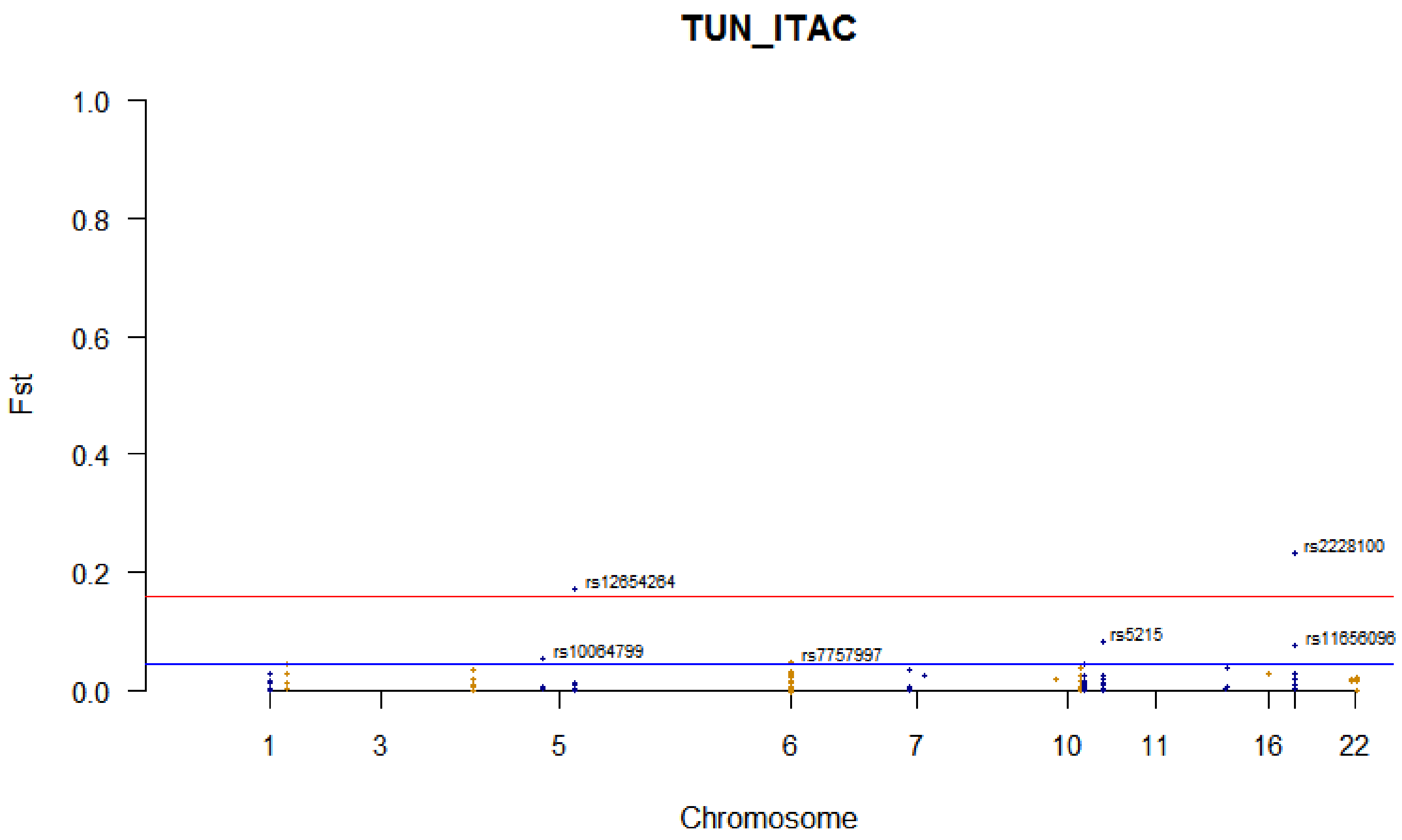

Manhattan plot of Fst comparison results. The X-axis represents the chromosomes; the Y-axis represents Fixation Index (Fst); a measure of population differentiation due to genetic structure; the horizontal blue line represents non differentiated variants; the horizontal red line points represent significant differentiated variants among Tunisian (TUN) and Central Italian (ITA-C) populations.

Supplementary Figure 1 next

Supplementary Figure 1: Manhattan Plot Fst result among studied populations

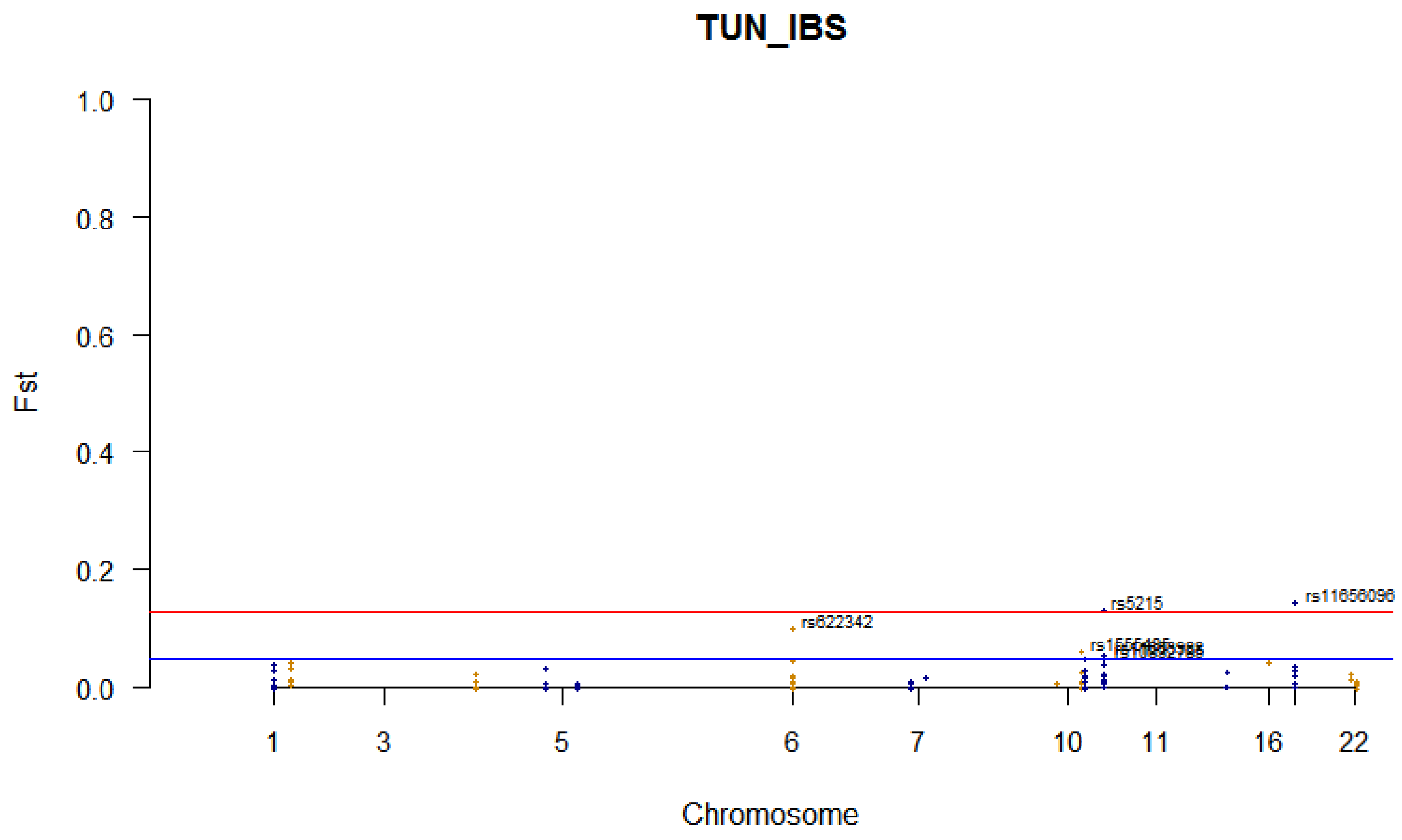

Manhattan plot of Fst comparison results. The X-axis represents the chromosomes; the Y-axis represents Fixation Index (Fst); a measure of population differentiation due to genetic structure; the horizontal blue line represents non differentiated variants; the horizontal red line points represent significant differentiated variants among Tunisian (TUN) and Iberian (IBS) populations.

Supplementary Figure 1 next

Supplementary Figure 1: Manhattan Plot Fst result among studied populations

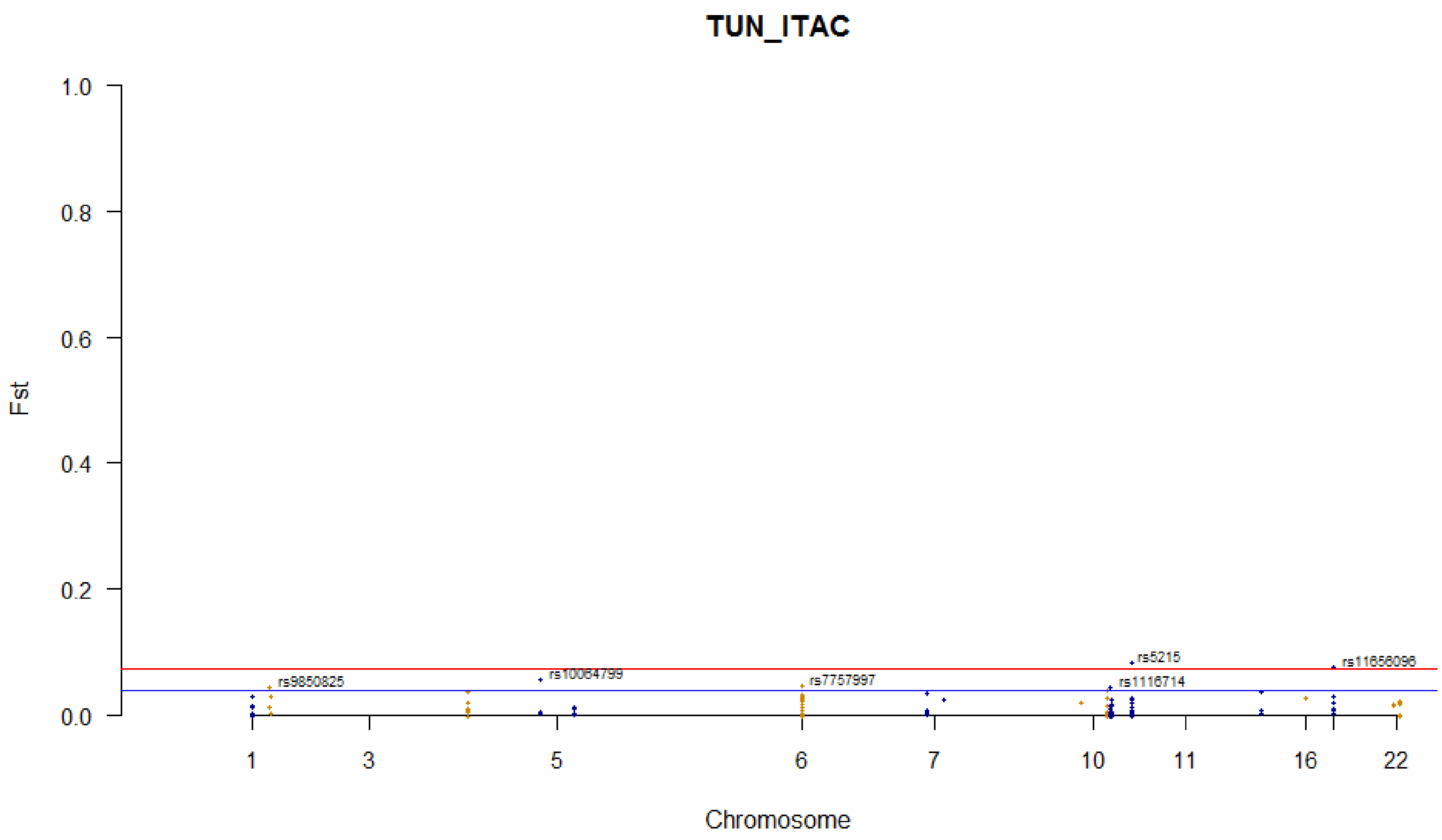

Manhattan plot of Fst comparison results. The X-axis represents the chromosomes; the Y-axis represents Fixation Index (Fst); a measure of population differentiation due to genetic structure; the horizontal blue line represents non differentiated variants; the horizontal red line points represent significant differentiated variants among Tunisian (TUN) and Iberian (IBS) populations.

Supplementary Figure 1 next

Supplementary Figure 1: Manhattan Plot Fst result among studied populations

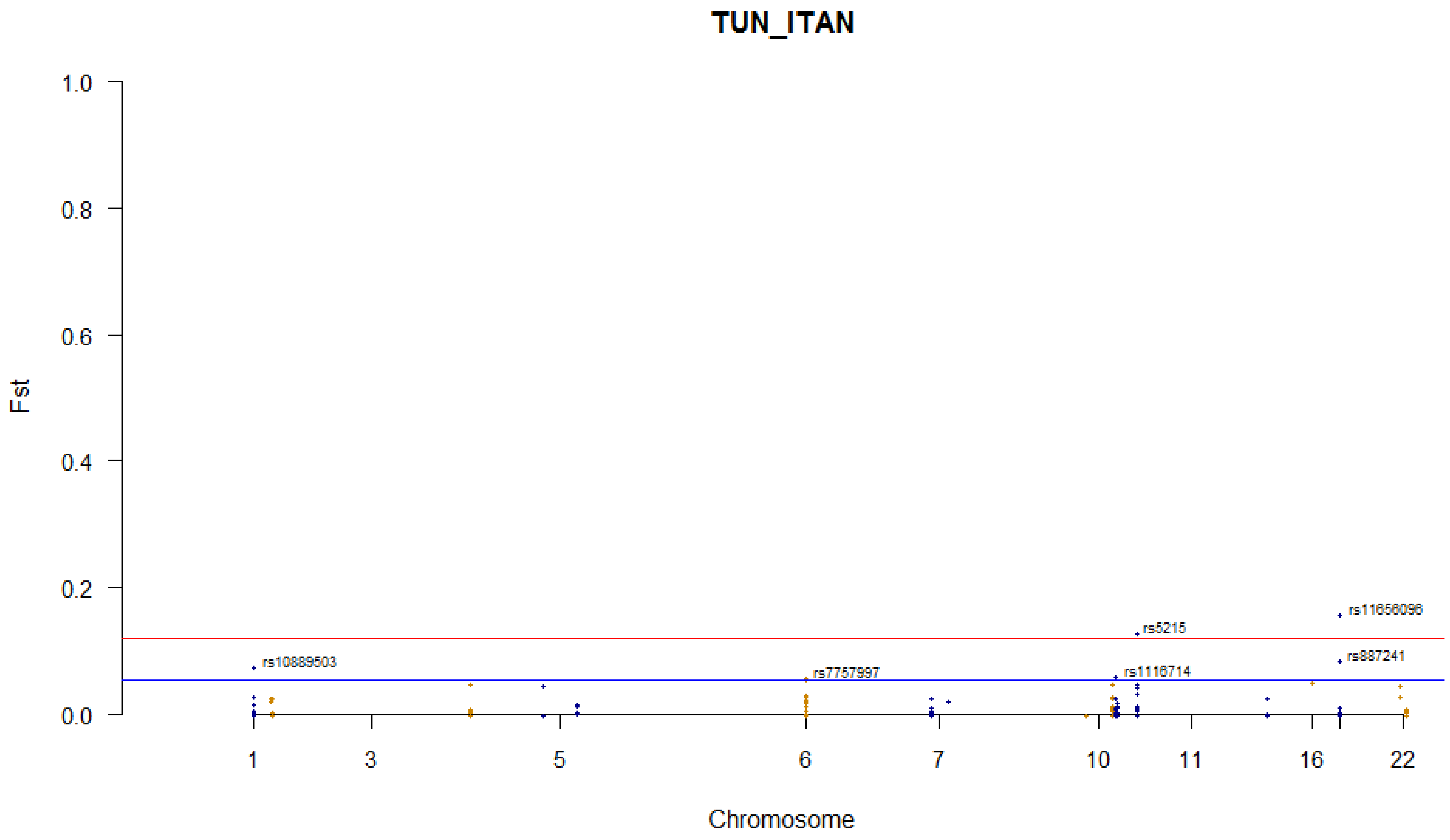

Manhattan plot of Fst comparison results. The X-axis represents the chromosomes; the Y-axis represents Fixation Index (Fst); a measure of population differentiation due to genetic structure; the horizontal blue line represents non differentiated variants; the horizontal red line points represent significant differentiated variants among Tunisian (TUN) and North Italian (N-ITA) populations.

Supplementary Figure 1 next

Supplementary Figure 1: Manhattan Plot Fst result among studied populations

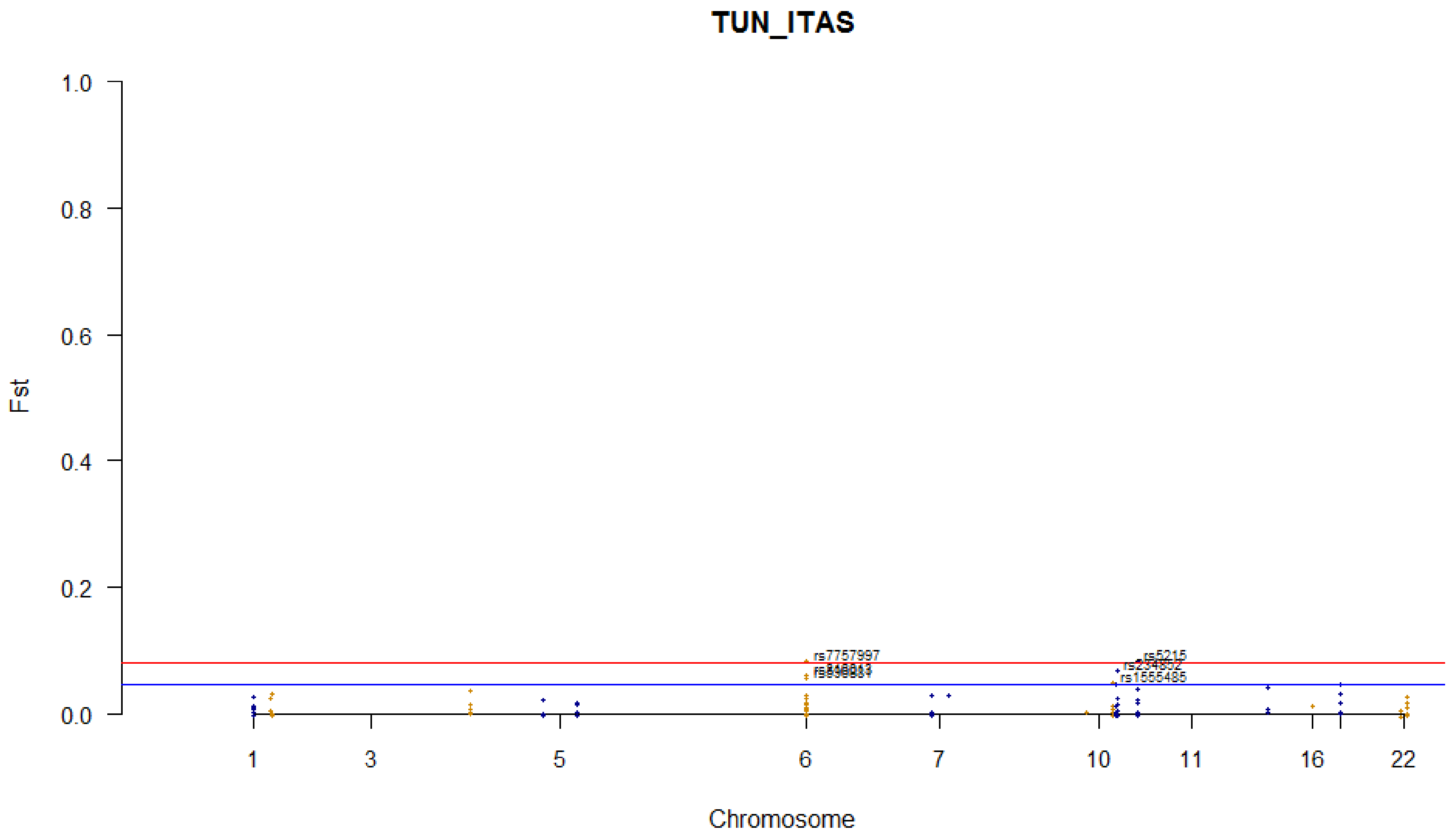

Manhattan plot of Fst comparison results. The X-axis represents the chromosomes; the Y-axis represents Fixation Index (Fst); a measure of population differentiation due to genetic structure; the horizontal blue line represents non differentiated variants; the horizontal red line points represent significant differentiated variants among Tunisian (TUN) and Sorth Italian (S-ITA) populations.

Supplementary Figure 1 next

Supplementary Figure 1: Manhattan Plot Fst result among studied populations

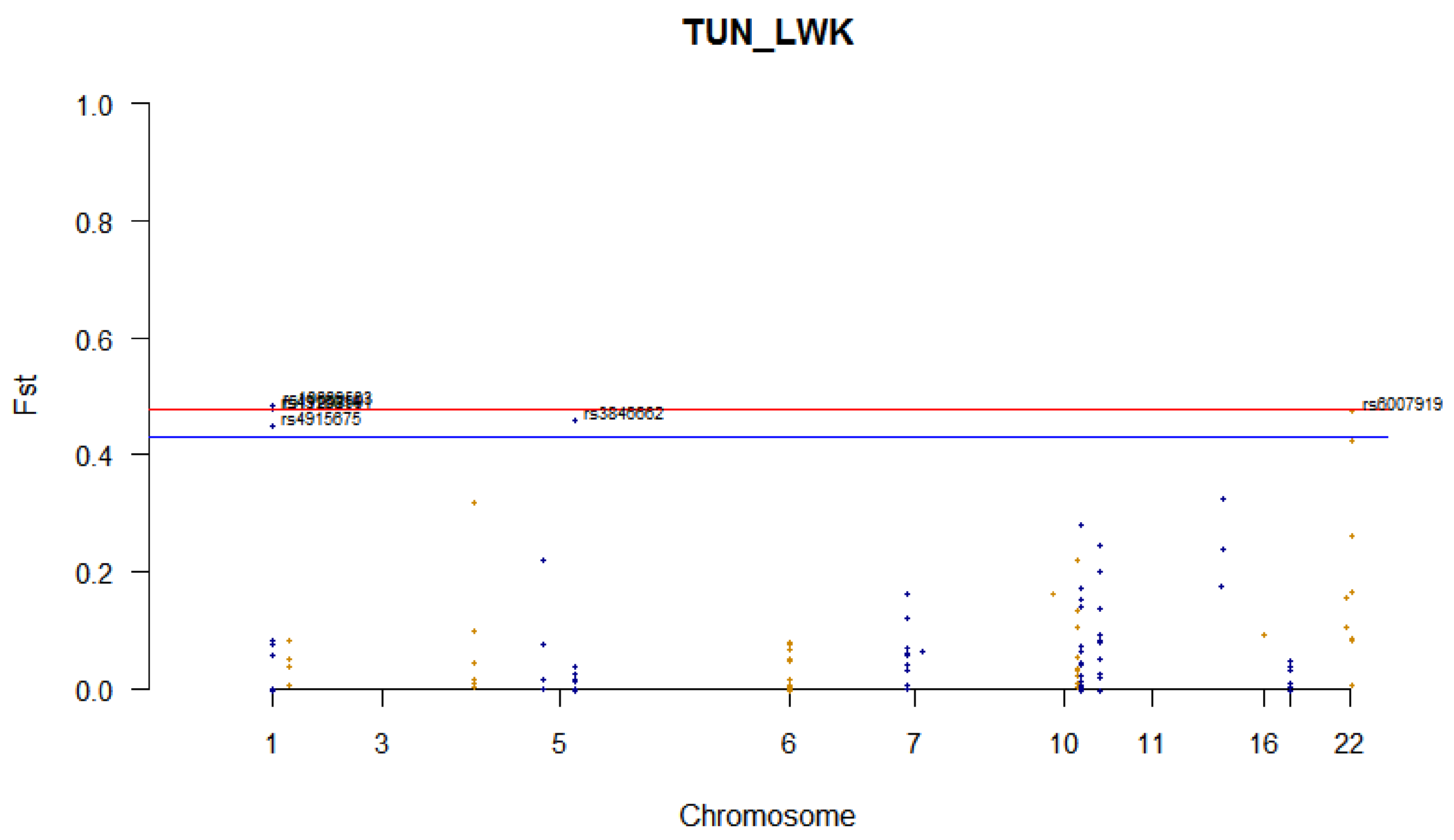

Manhattan plot of Fst comparison results. The X-axis represents the chromosomes; the Y-axis represents Fixation Index (Fst); a measure of population differentiation due to genetic structure; the horizontal blue line represents non differentiated variants; the horizontal red line points represent significant differentiated variants among Tunisian (TUN) and Luhya (LWK) populations.

Supplementary Figure 1 next

Supplementary Figure 1: Manhattan Plot Fst result among studied populations

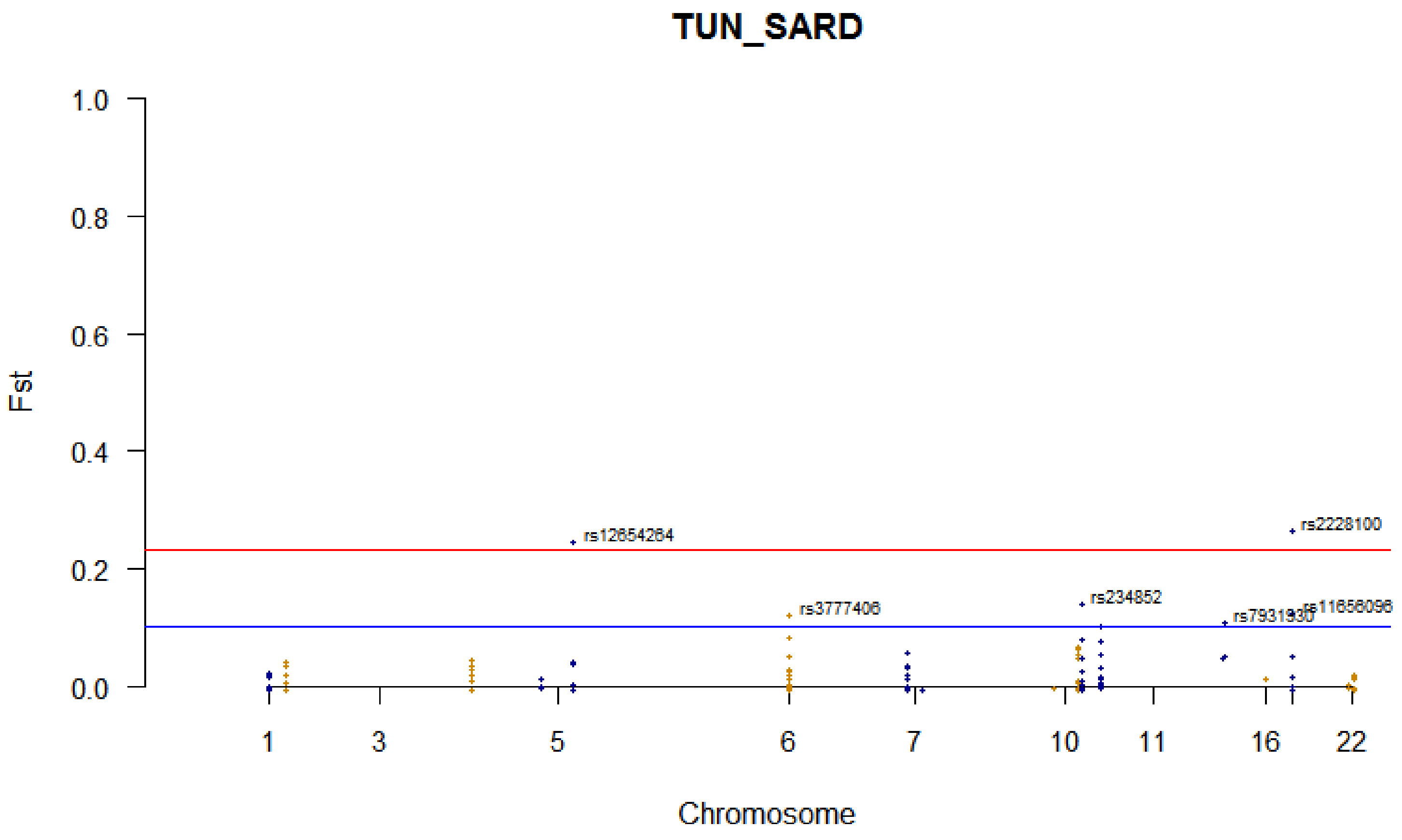

Manhattan plot of Fst comparison results. The X-axis represents the chromosomes; the Y-axis represents Fixation Index (Fst); a measure of population differentiation due to genetic structure; the horizontal blue line represents non differentiated variants; the horizontal red line points represent significant differentiated variants among Tunisian (TUN) and Sardinian (SARD) populations.

Supplementary Figure 1 next

Supplementary Figure 1: Manhattan Plot Fst result among studied populations

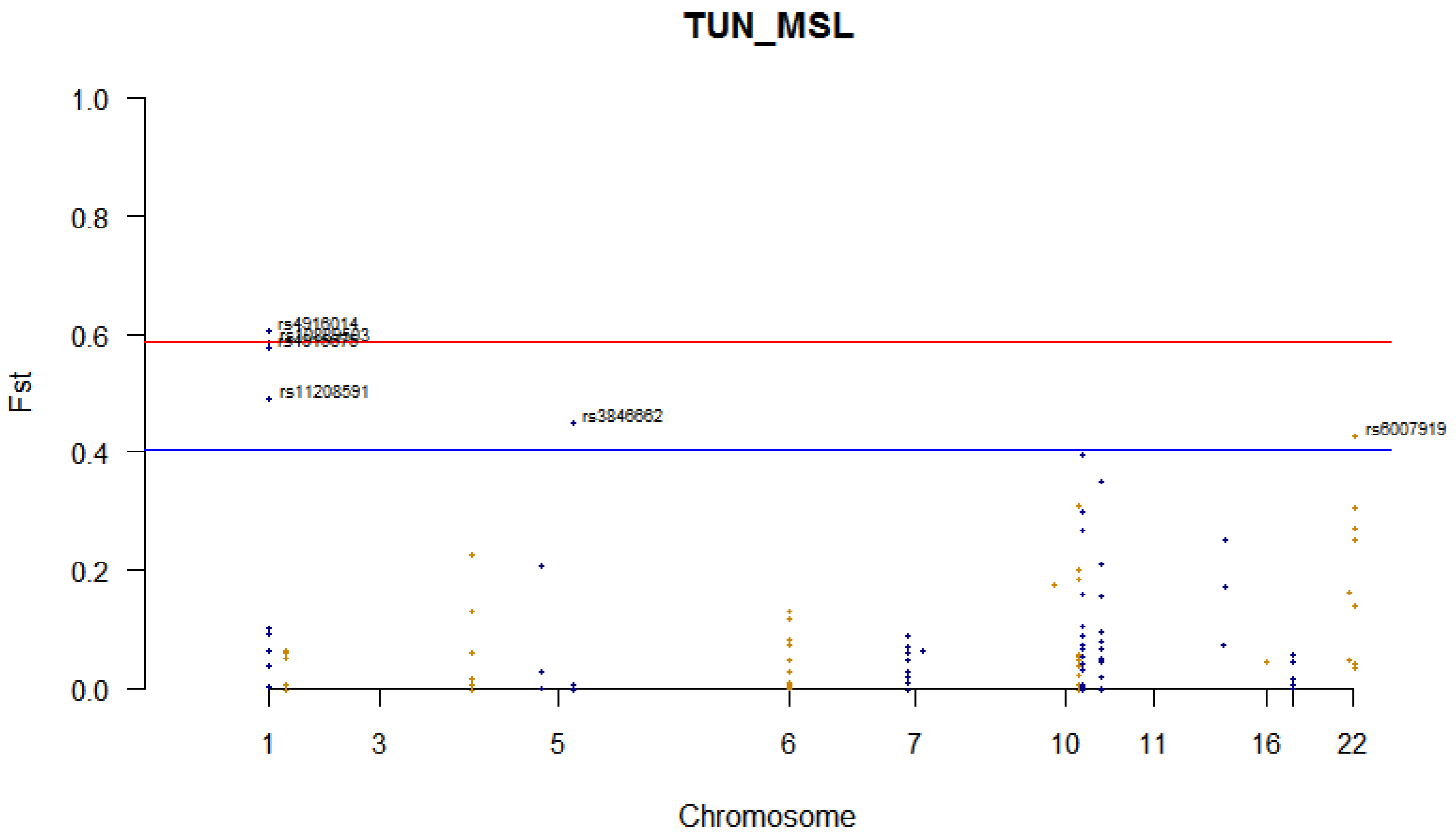

Manhattan plot of Fst comparison results. The X-axis represents the chromosomes; the Y-axis represents Fixation Index (Fst); a measure of population differentiation due to genetic structure; the horizontal blue line represents non differentiated variants; the horizontal red line points represent significant differentiated variants among Tunisian (TUN) and Mende (MSL) populations.

Supplementary Figure 1 next

Supplementary Figure 1: Manhattan Plot Fst result among studied populations

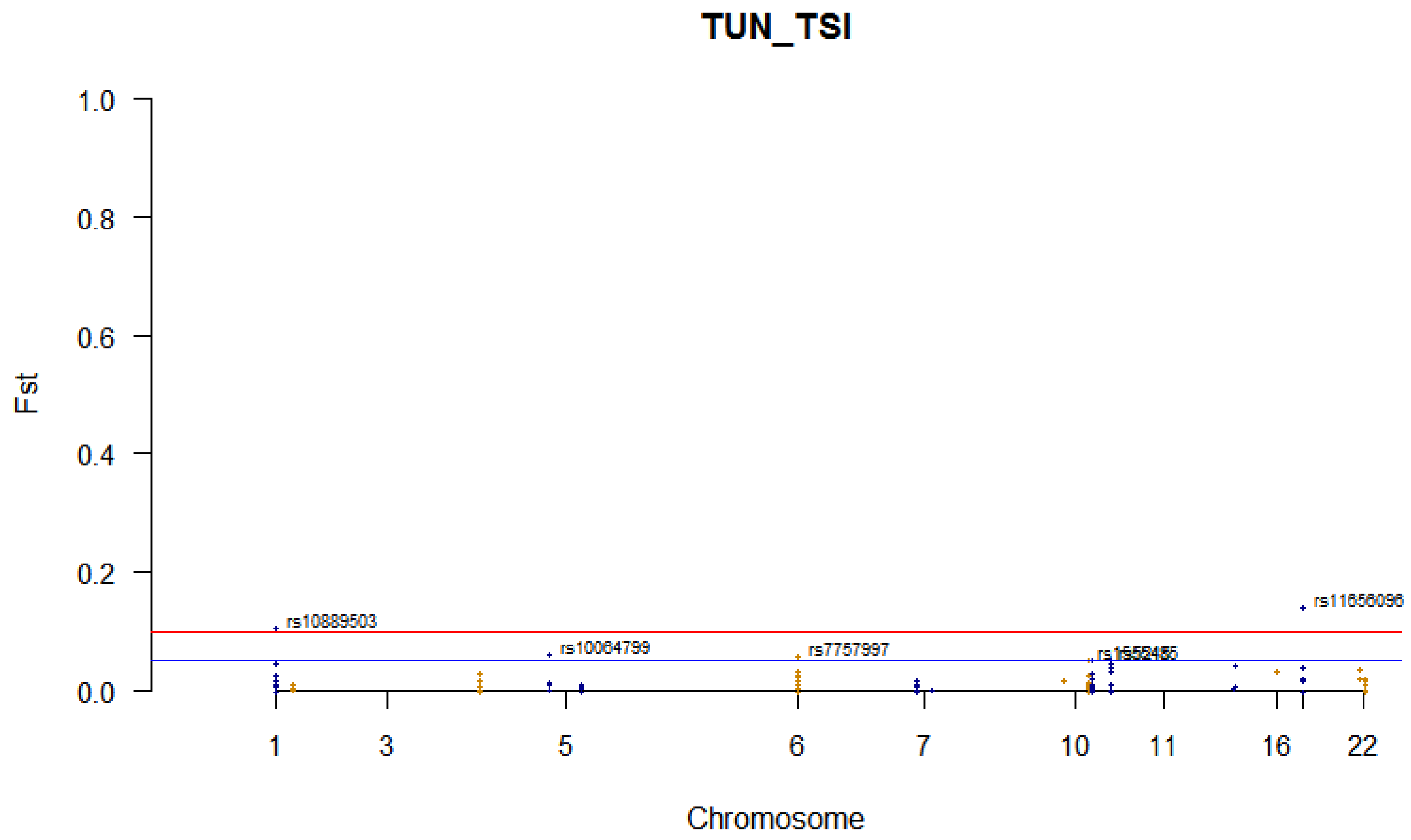

Manhattan plot of Fst comparison results. The X-axis represents the chromosomes; the Y-axis represents Fixation Index (Fst); a measure of population differentiation due to genetic structure; the horizontal blue line represents non differentiated variants; the horizontal red line points represent significant differentiated variants among Tunisian (TUN) and Toscan (TSI) populations.

Supplementary Figure 1 next

Supplementary Figure 1: Manhattan Plot Fst result among studied populations

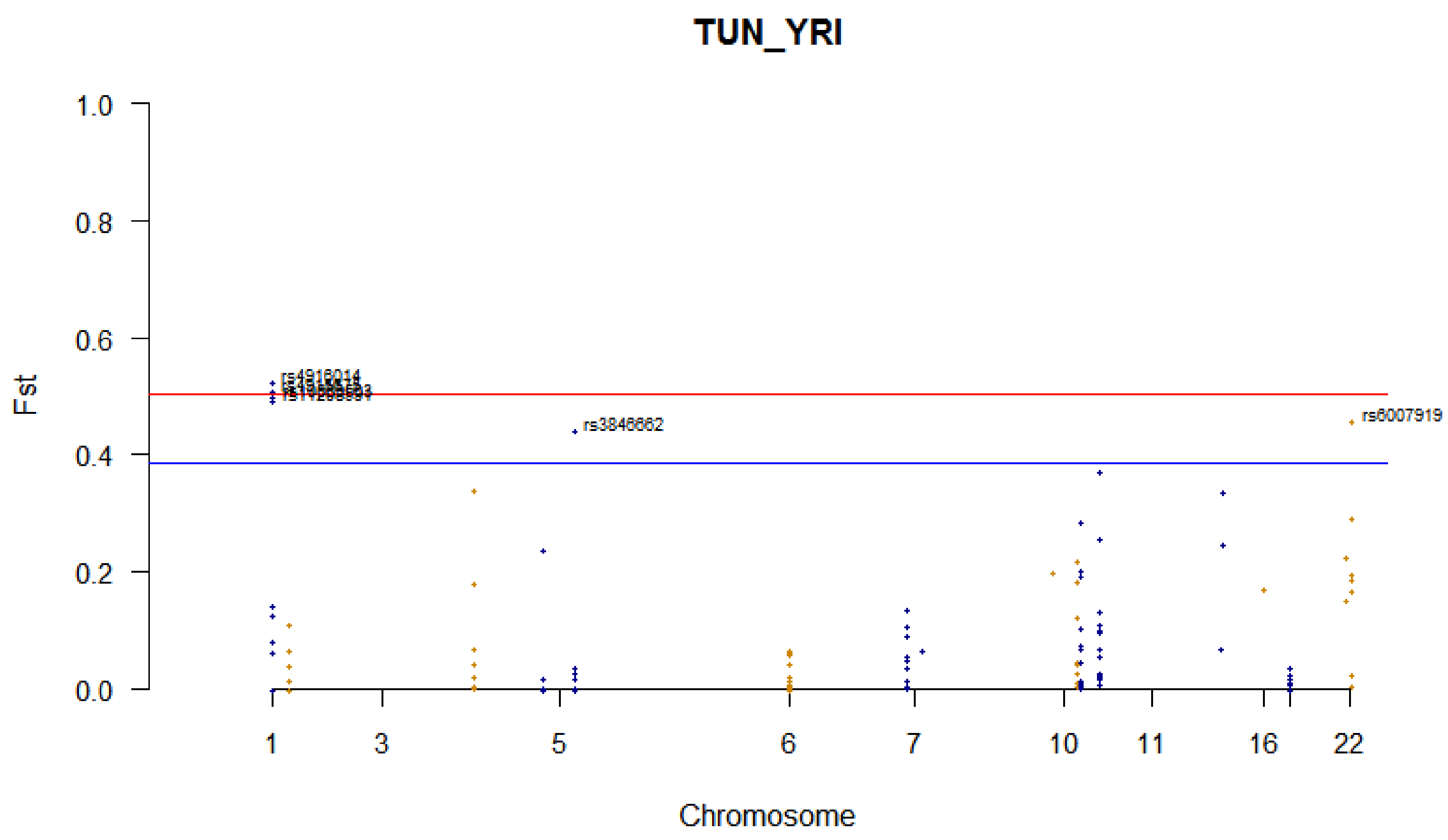

Manhattan plot of Fst comparison results. The X-axis represents the chromosomes; the Y-axis represents Fixation Index (Fst); a measure of population differentiation due to genetic structure; the horizontal blue line represents non differentiated variants; the horizontal red line points represent significant differentiated variants among Tunisian (TUN) and Yoruba (YRI) populations.

Supplementary Figure 1 next
